# Interactome Profiling of a Lysine Deacetylase Trapping Probe Library Uncovers Crosstalk Between HDAC6 and NF‐κB Signaling

**DOI:** 10.1002/anie.202510967

**Published:** 2025-08-18

**Authors:** Julian Seidel, Caroline Schönfeld, Julia Sindlinger, Jürgen Eirich, Sören Kirchgäßner, Nina Kreienkamp, Klaus Schulze‐Osthoff, Iris Finkemeier, Stephan Hailfinger, Dirk Schwarzer

**Affiliations:** ^1^ Interfaculty Institute of Biochemistry (IFIB) University of Tübingen Auf der Morgenstelle 34 72076 Tübingen Germany; ^2^ Institute of Plant Biology and Biotechnology University of Münster Schlossplatz 7 48149 Münster Germany; ^3^ Department of Medicine A, Hematology, Oncology, and Pneumology University Hospital Münster Albert‐Schweitzer‐Campus 1 48149 Münster Germany

**Keywords:** HDAC inhibitor, HDAC6, Lysine acetylation, NF‐κB, Peptide probes

## Abstract

Lysine or histone deacetylases (HDACs) remove acetyl groups from lysine residues of numerous proteins, thereby regulating their function and activity. HDAC6 is involved in multiple cellular processes, yet its protein interaction network remains poorly understood. To uncover novel HDAC6 substrates, we performed an acetylome analysis of HDAC6 knockdown cells, which served as a basis for the design of an HDAC6‐trapping peptide library containing hydroxamic acids. Most probes enriched HDAC6 from cell lysates stronger than HDAC1. Proteomic profiling of the trapping probes revealed a preferential enrichment of HDAC6 and resulted in the identification of novel putative HDAC6 interaction partners. Among those were several components of the pro‐inflammatory transcription factor NF‐κB that were independently confirmed as HDAC6 binders. Mechanistically, HDAC6 counteracted NF‐κB activity induced upon p300‐catalyzed acetylation of NF‐κB p50. These findings indicate a potential anti‐inflammatory function of HDAC6 in NF‐κB signaling.

## Graphical abstract

We designed a peptide probe library containing hydroxamic acids based on putative substrate sites of lysine deacetylase HDAC6. Analyzing substrate selectivity and turnover combined with proteomics profiling resulted in identification of novel HDAC6 interaction partners, such as components of the transcription factor NF‐κB. Importantly, HDAC6 counteracted the activity of acetylated NF‐κB p50, indicating a potential role in NF‐κB signaling.

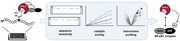

## Introduction

**Figure 1:**
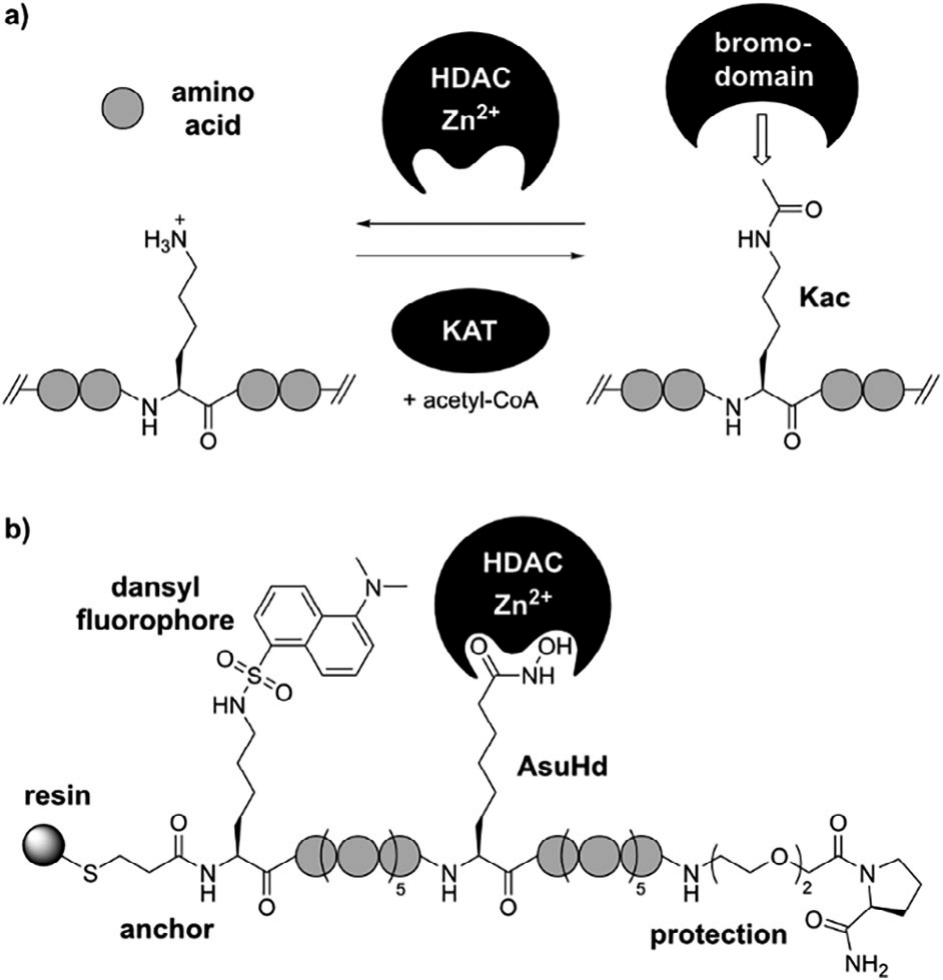
Lysine acetylation and HDAC probes: a) Lysine acetylation is installed by lysine acetyltransferases (KATs) using acetyl‐coenzyme A (acetyl‐CoA) as a co‐substrate and removed by lysine or histone deacetylases (KDACs / HDACs). Acetyllysine (Kac) serves as binding site for bromodomains. b) General design of peptide probes for HDAC6 interactome profiling. Probe peptides were derived from known acetylation sites serving as potential substrates of HDAC6. The Kac residue was replaced by AsuHd, and seven flanking residues were introduced N‐ and C‐terminally to the modification site. The C‐terminus was protected against carboxypeptidases by a *D*‐Pro‐amide and polyethyleneglycol cap. The N‐terminus was extended by a Lys(Dns)‐3‐mecaptopropionic acid moiety introduced as a single building block. Full‐length peptides were immobilized from the crude product on agarose resin by thiol‐capture after adjusting peptide concentrations based on the dansyl (Dns) fluorescence.

Lysine acetylation constitutes a highly abundant posttranslational modification (PTMs) of proteins in all kingdoms of life.^[^ ^1^, ^2^, ^3^, ^4^ ^]^ Following its initial discovery on histones, thousands of acetylated proteins have been uncovered, but the functions of the majority of these modifications remain elusive.^[^ ^5^ ^]^ Lysine acetylation is known to regulate protein function, activity and cellular localization.^[^ ^6^, ^7^, ^8^ ^]^ These regulatory functions are mediated by two effects: On the one hand, acetylated lysine residues serve as recruitment sites for acetyllysine (Kac)‐binding domains, referred to as bromodomains, which recognize this PTM in a site‐specific manner (Figure 1a).^[^ ^9^, ^10^ ^]^ On the other hand, formation of the *N*ε‐amide in Kac neutralizes the positive charge of the lysine's side chain.^[^ ^11^ ^]^ Chemical probes have been instrumental for investigating lysine acetylation, bromodomains and the enzymes involved in adding or removing this PTM.^[^ ^12^, ^13^, ^14^, ^15^, ^16^, ^17^, ^18^, ^19^, ^20^ ^]^ Acetylation of proteins is controlled by the opposed actions of lysine or histone acetyltransferases (KATs/HATs) and deacetylases (KDACs/HDACs), respectively (Figure 1a).^[^ ^21^, ^22^, ^23^ ^]^ HDACs, classified into class I, IIa, IIb, and IV deacetylases, require Zn^2+^ for catalytic activity and share homology with yeast deacetylases RPD3 and Hda1, the founding members of the HDAC family.^[^ ^24^, ^25^ ^]^ In addition, class III lysine deacetylases, referred to as sirtuins, are known to deacetylate Kac by a distinct reaction mechanism depending on NAD^+^ as a co‐factor.^[^ ^6^ ^]^

The Zn^2+^‐dependent HDACs, hereafter referred to as HDACs, are frequently overexpressed in various types of cancer, making them promising drug targets.^[^ ^26^ ^]^ Several natural products and synthetic small molecules with inhibitory activity towards HDACs were uncovered, some of which are even approved for clinical use in cancer therapy.^[^ ^27^ ^]^ The activity of most inhibitors is based on their metal‐chelating properties, like hydroxamic acids, that bind tightly to the active site Zn^2+^ ion. HDACs interact with various proteins that potentially impact their catalytic activity and specificity.^[^ ^28^ ^]^ Investigations on these HDAC‐imbedding multiprotein complexes is hampered by their highly challenging in vitro reconstitution. As an alternative, research approaches have focused on probes for proteome‐wide profiling of endogenous HDACs in cellular lysates.^[^ ^29^, ^30^ ^]^ In order to profile HDAC complexes in a substrate site‐dependent manner, HDAC‐trapping amino acids have been used as substitutes of the Kac moiety in peptides derived from known HDAC substrate sites. These HDAC‐trapping amino acids bind tightly to the active sites of HDACs and are not processed by the enzymes, thereby competing with endogenous substrates for the HDACs' active sites.^[^ ^12^ ^]^ Several HDAC‐trapping residues have been developed by grafting binding groups of HDAC inhibitors onto amino acid scaffolds.^[^ ^12^, ^16^, ^31^, ^32^, ^33^ ^]^ Peptide probes containing these residues have been used to enrich endogenous HDACs from native cell lysates or for profiling the substrate specificity of recombinant deacetylases.^[^ ^12^, ^16^, ^34^ ^]^ HDAC‐trapping 2‐amino‐8‐(hydroxyamino)‐8‐oxooctanoic acid (AsuHd) was shown to interact strongly with the majority of HDACs, making it the most commonly used amino acid for this approach.^[^ ^12^, ^16^ ^]^ In general, synthetic peptides have been proven to be very useful tools for studying PTM‐dependent protein‐protein interactions, despite the fact that they only represent short fragments of a folded full‐length protein. This is particularly true for intrinsically disordered protein regions where PTMs accumulate and which can be effectively mimicked by synthetic peptides.^[^ ^35^ ^]^

Among the eleven mammalian HDACs, HDAC6 stands out because it possesses a tandem‐deacetylase domain.^[^ ^36^ ^]^ In addition, HDAC6 contains a putative ubiquitin‐binding domain and localizes to the cytoplasm and the nucleus, where it plays key roles in cytoskeleton organization, protein folding and transcriptional regulation.^[^ ^37^ ^]^ The multiple roles of HDAC6 are still incompletely understood. It appears likely that a multitude of HDAC6‐binding proteins are required to modulate its activity. While a set of HDAC6‐interacting proteins has already been identified, the complex cellular functions of HDAC6 indicate that additional interaction partners might exist.^[^ ^38^, ^39^, ^40^, ^41^, ^42^, ^43^ ^]^

In this study, we developed a strategy for profiling endogenous HDACs and HDAC complexes in a microarray format. Screening such arrays with endogenous HDACs of cellular lysates should provide new insights into native substrates and binding partners without confounding effects of HDAC overexpression which could deregulate gene expression on a global cellular level. We adapted the established technologies of probe synthesis and pulldown assays to 96‐well microtiter formats enabling efficient throughput. Focusing on HDAC6, we first identified potential substrate sites by acetylome profiling of HDAC6 knockdown cells and derived 96 peptides covering high‐affinity, low‐affinity and control probes for enriching endogenous HDAC6 from cell lysates. Four probes showing preferred HDAC6 recruitment were further characterized by interactome profiling and biochemical assays, uncovering a broad set of known and new HDAC6 interaction partners. One of the novel HDAC6 binders – the transcription factor NF‐κB component p100 – was further investigated.^[^ ^44^ ^]^ The precursor protein NF‐κB p100 and its homologue p105 as well as their proteolytically matured forms p50 and p52 were confirmed as HDAC6‐binding proteins. We further showed that NF‐κB p50 was indeed deacetylated by HDAC6. When investigating potential roles of HDAC6, we observed that p300/CBP‐mediated acetylation of NF‐κB p50 enhanced NF‐κB activity, which was attenuated by HDAC6. Hence, our data indicates a potential anti‐inflammatory activity of HDAC6 in the NF‐κB signaling pathway.

## Results and Discussion

### Discovering HDAC6 Substrate Sites by Acetylome Profiling and Peptide Library Design

In a first step, we investigated putative HDAC6 substrate sites that could provide the peptide‐context of hydroxamic acid‐containing probes targeting HDAC6. To this end, we compared the acetylomes of HeLa cells subjected to RNAi knockdown of HDAC6 and cells transfected with non‐targeted RNAi. Acetylomes were analyzed by high‐resolution LC‐MS/MS after isotopic labeling of tryptic peptides with formaldehyde by reductive amination (dimethyl labeling). Prior to LC‐MS/MS analysis, acetylated peptides were enriched on an anti‐acetyllysine antibody‐conjugated resin, and acetylation sites were identified and quantified using the MaxQuant software package.^[^ ^1^, ^2^, ^6^ ^]^ The experiments were performed in four biological replicates implementing label swaps between HDAC6 knockdown and control acetylomes. Successful HDAC6 knockdown was confirmed by log2‐transformed depletion ratios ranging from −1.69 to −2.59 in HDAC6 knockdown cells compared to the non‐targeted controls (Table S1). In total, we identified 2015 lysine acetylation sites in the HDAC6 knockdown acetylome. Potential HDAC6 substrate sites are indicated by a higher abundance in the HDAC6 knockdown acetylome compared to the control. The cut‐off was set to an upregulation of >1.5‐fold (≥ log_2_ 0.6‐fold). We identified 810 acetylation sites that fulfilled these criteria (Table S2). Despite the original assignment as histone deacetylase, up‐regulated acetylation of histones was not among the most pronounced modifications upon HDAC6 knockdown. This observation is in agreement with the notion that HDACs 1–3 serve as the main deacetylases of histones.^[^ ^45^ ^]^ Consequently, a set of 16 identified putative non‐histone HDAC6 substrate sites was selected for the probe design. These sites were derived from proteins involved in central cell functions where HDAC6 has been implicated as a putative regulator. The probe sequences were completed by nine HDAC6 substrate sites uncovered previously by biochemical assays (αTub‐probe),^[^ ^39^ ^]^ or in cells treated with HDAC6‐specific inhibitor tubacin,^[^ ^46^ ^]^ and two peptide motifs previously used for HDAC trapping probes (mini‐probe and p53‐probe).^[^ ^12^ ^]^ These sequences were selected to cover a broad range of biological functions with a potential regulatory role of HDAC6 (Table S3). Finally, five peptides derived from acetylation sites that were not upregulated upon HDAC6 knockdown served as negative controls. In total, 32 peptide sequences were selected for the design of the HDAC probe library.

### Synthesis of Building Blocks and HDAC6‐Targeting Peptide Probes

The key step for adapting the HDAC‐trapping peptide probes to high‐throughput formats was avoiding lengthy purification processes of the probe peptides. The need to purify synthetic peptides containing HDAC‐trapping hydroxamic acid building blocks like AsuHd arises mainly from two types of side products: 1.) peptides with incompletely removed *tert*‐butyl groups from the AsuHd(O*t*Bu) building block,^[^ ^12^ ^]^ and 2.) truncation products resulting from incomplete coupling reactions.

We addressed the first problem by replacing the *tert*‐butyl group with the more acid‐labile trityl (Trt) protectant. The highly acid labile trityl group was installed by altering our initial synthesis route of the AsuHd building block (Scheme S1).^[^ ^31^, ^47^ ^]^ We synthesized two trityl protected building blocks: 2‐amino‐8‐(hydroxyamino)‐8‐oxooctanoic acid, AsuHd (Fmoc‐AsuHd(OTrt)‐OH), which binds tightly to HDACs of class I and II, and 2‐amino‐7‐(hydroxyamino)‐7‐oxoheptanoic acid, ApmHd (Fmoc‐ApmHd(OTrt)‐OH), which binds HDACs with lower affinity.^[^ ^12^ ^]^ Both building blocks were synthesized as racemate. We have previously observed that peptide probes containing racemic AsuHd enrich endogenous HDACs sufficiently in pulldowns for subsequent western blot analysis.^[^ ^31^ ^]^

A resin‐capture strategy was used to solve the second problem.^[^ ^48^ ^]^ In this case, an N‐terminal 3‐mercaptopropionic acid (Mpa) moiety allowed immobilization of the probe peptides on iodoacetyl‐conjugated agarose (Figure 1b). During synthesis, each coupling step was followed by a capping reaction with acetic anhydride, ensuring that only full‐length peptides were equipped with the N‐terminal thiol. The anchoring thiol was installed as a single Trt‐Mpa‐Lys(Dns)‐OH building block (Scheme S2), which included a dansyl (Dns) fluorophore for quantifying the amounts of full‐length peptide in the crude product.

**Figure 2:**
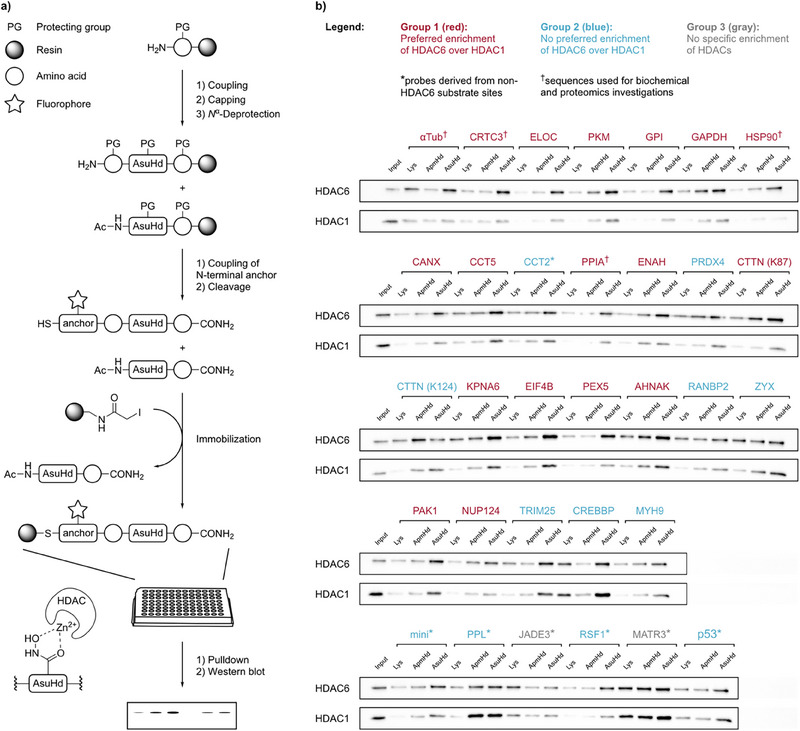
Assembly of HDAC‐trapping peptide probe library and HDAC6 profiling: a) Probe peptides were assembled by solid‐phase peptide synthesis implementing a capping step after each coupling reaction. The anchoring building block including a thiol for covalent capture and a fluorophore for quantification was incorporated in the last step into full‐length peptides. After immobilization on iodoacetyl‐conjugated agarose, the resins were transferred into filter‐bottom 96‐well plates. Subsequent pulldowns from native HeLa cell lysates were analyzed by western blotting and probed with antibodies against HDAC6 and HDAC1. The experiments were reproducible and performed in three replicates. b) Western blot analysis of HDAC6 and HDAC1. HeLa lysate (200 µg of total protein) was treated with high‐affinity (AsuHd), low‐affinity (ApmHd) and non‐binding control (Lys) probes derived from 32 acetylation sites of the indicated proteins. The binding patterns were assigned to three groups based on the western blot signals. Group 1 (red) showed preferred enrichment of HDAC6 over HDAC1. Group 2 (blue) showed preferred enrichment of HDAC1 over HDAC6 or comparable enrichment of both enzymes. Group 3 (gray) showed no specific recruitment of HDACs to any of the trapping probes. Four probes of group 1 (marked with a dagger †) were further analyzed as HDAC6 substrates and used for proteomics‐based interactome profiling. Input samples: 16 µg. Uncropped illustrations of all blots are shown in the Supporting Information.

Based on this strategy, we generated the peptide library for HDAC6 profiling. The acetylation sites of the 32 peptide probe sequences were centered in synthetic 15‐mer peptides and replaced by either AsuHd as high‐affinity probes, by ApmHd as low‐affinity probes, or by lysine serving as non‐binding controls (Figure S1). All 96 peptides were synthesized in parallel by optimized automated SPPS and subsequently cleaved off the solid support in a microtiter plate. Crude peptides were then immobilized on iodoacetyl‐conjugated agarose resin after adjusting their concentrations based on the dansyl fluorescence intensity. Finally, the immobilized peptide probes were transferred into filter‐bottom 96‐well plates forming the HDAC‐trapping library (Figure 2a).

### High‐Throughput Pulldowns with the HDAC6‐Targeting Peptide Probe Library

In the next step, we subjected the library to pulldown experiments with HeLa cell lysates. The lysates were transferred to the library plate, incubated with the probes, and after removal of the supernatant, bound proteins were eluted. The eluted samples were then resolved by SDS‐PAGE, followed by western blot analysis with antibodies against HDAC6, while antibodies against HDAC1 served as a reference for the specificity of HDAC6 enrichment. The analysis showed that both HDAC6 and HDAC1 bound to most probes containing AsuHd (Figure 2b). However, the interaction profiles varied between probes and could be assigned to three groups: Probes of group 1 showed a preferred enrichment of HDAC6 over HDAC1 when containing AsuHd. In some cases, HDAC6 was even enriched on the low‐affinity ApmHd probes. In total 18 probes could be assigned to group 1, all of which were derived from putative HDAC6 substrate sites (Figure 2b, highlighted in red). Probes of group 2 (highlighted in blue in Figure 2b) showed a similar enrichment of HDAC6 and HDAC1 or even a preferred enrichment of the latter when containing AsuHd. Group 3 probes (highlighted in grey in Figure 2b), derived from MATR3 and JADE3, enriched HDAC1 and HDAC6 on the lysine controls which indicated unspecific HDAC binding (Figure 2b). Only probe sequences of group 1 were considered as potential HDAC6 substrates and four of these probes were selected for further investigation. These included the αTub‐probe derived from the K40 site of α‐tubulin, the CRTC3‐probe containing the sequence flanking K113 of CREB‐regulated transcription coactivator 3, the PPIA‐probe derived from the K28 site of PPIA, and the HSP90‐probe covering the acetylation site K191 of heat shock protein HSP90. Acetylated HSP90 and α‐tubulin are known substrates of HDAC6,^[^ ^39^, ^49^ ^]^ whereas little information exists regarding a potential crosstalk of HDAC6 and CRTC3 or PPIA, also referred to as cyclophilin A. The latter was reported to regulate HDAC6 expression by modulating the assembly of transcription factor complexes.^[^ ^50^ ^]^ A closer inspection of the interaction network of the PPIA‐probe appeared promising for the investigation of new biological functions of HDAC6.

### Validation Assays and Interactome Profiling of HDAC6‐Targeting Probes

To validate that the acetylation sites of the αTub‐, CRTC3‐, HSP90‐, and PPIA‐probes are substrates of HDAC6, we re‐synthesized the peptides with Kac instead of AsuHd at the modification sites (Table S4 and Figure S2) and resorted to an established deacetylase assay based on MALDI‐TOF MS as readout.^[^ ^51^ ^]^ The deacetylation assays were performed with recombinant HDAC6 and showed that the recombinant enzyme was able to deacetylate all peptide substrates, indicating that these acetylation sites could serve as *bona fide* HDAC6 substrates (Figure S3).

To uncover new HDAC6 substrates and binding partners, we next studied the interactome of the AsuHd‐containing αTub‐, CRTC3‐, HSP90‐, and PPIA‐probes. The probe peptides were re‐synthesized, however, the probe design was changed by replacing the previously used racemic AsuHd with the enantiopure l‐residue to enhance HDAC enrichment efficiency. The re‐synthesized peptide probes were further immobilized via a C‐terminal Cys residue to ensure that the immobilization site did not perturb HDAC recruitment (Table S5 and Figure S4). Western blot analysis of pulldowns from HeLa lysates confirmed a strong recruitment of HDAC6 to the AsuHd‐containing re‐synthesized probes when compared to HDAC1. By contrast, only the broadly specific mini‐AsuHd probe enriched HDAC1 efficiently (Figure S5). In the case of the CRTC3‐probes, we observed a significant recruitment of HDAC6 to the CRTC3‐Lys control, indicating that the C‐terminal immobilization might enhance binding of HDAC6 to the deacetylated product.

**Figure 3:**
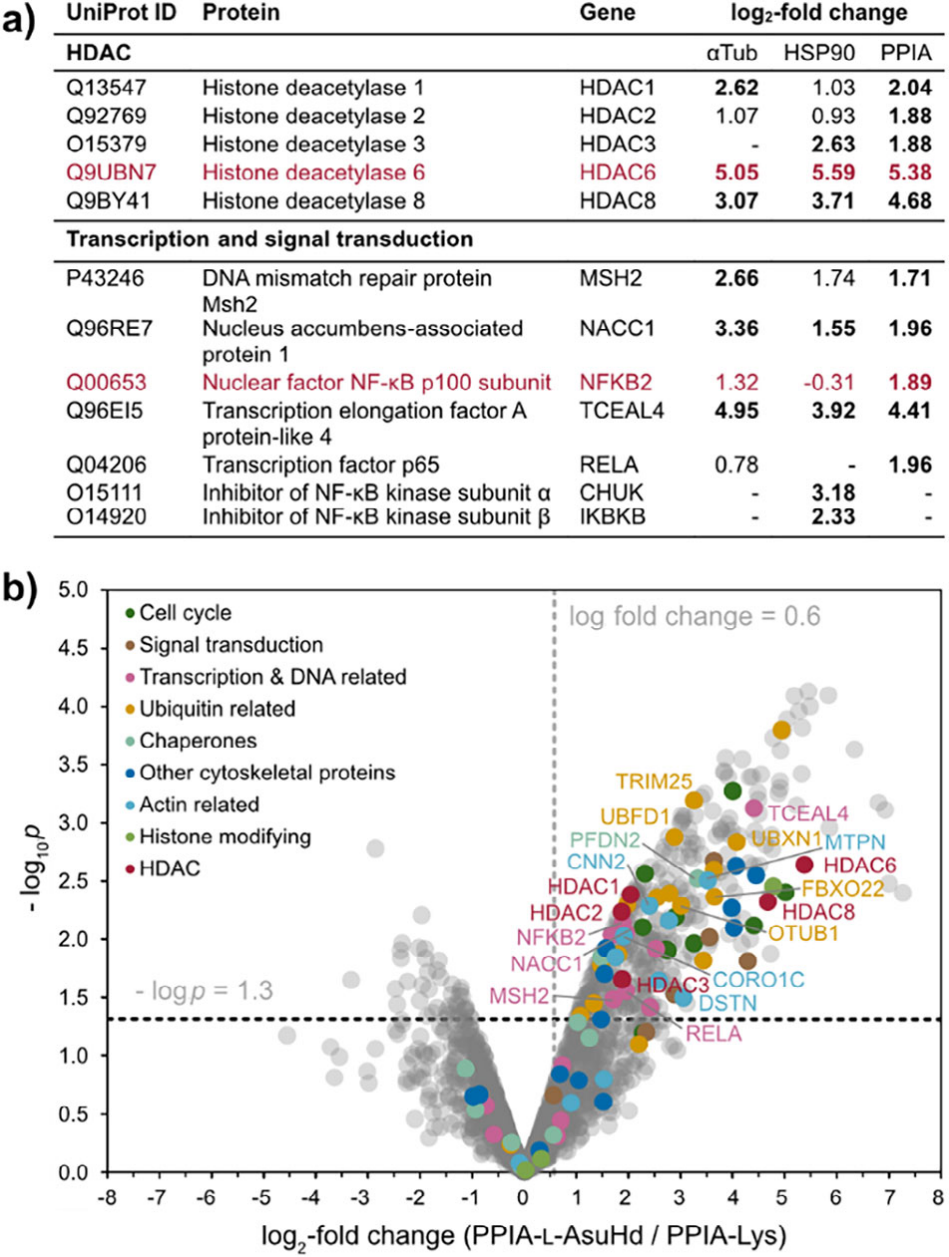
Interactome profiling of selected HDAC6‐trapping probes: a) HDACs and transcription and signal transduction regulators identified and quantified in the αTub‐AsuHd, HSP90‐AsuHd and PPIA‐AsuHd pulldowns. The enrichment on AsuHd versus Lys probes is indicated as a log_2_‐fold change. Enrichment with statistical significance (*p* ≤ 0.05 according to LIMMA analysis, three biological replicates) is marked by bold script. HDAC6 and the NF‐κB p100 protein are marked in red. b) Volcano plot illustrating protein enrichment on PPIA‐AsuHd over the lysine control. Log_2_‐fold changes are plotted against the negative logarithmic p‐value obtained from three biological replicates. Cut‐offs were set to −log_10_ *p* > 1.3 (*p* ≤ 0.05) and log_2_‐fold enrichment ≥ 0.6. Proteins significantly enriched on AsuHd‐probes over Lys‐controls locate to the upper right quadrant of the volcano plot. Enriched proteins were assigned by function to: HDACs, histone‐modifying enzymes, actin and cytoskeletal proteins, chaperones, proteins related to ubiquitin, and transcription, signal transduction, and cell cycle regulators.

In the following, we profiled the interactomes of these probes by high‐resolution mass spectrometry (Tables S6–S10). Proteins recovered from the resins after pulldowns were alkylated, digested, and subsequently analyzed by LC‐MS/MS. Label‐free quantification by MaxLFQ was used to determine the enrichments of proteins on the AsuHd‐containing probes in comparison to the Lys controls. Protein enrichments of three replicates were plotted as log_2_‐fold changes against the negative logarithmic p‐value of the statistical analysis. The resulting volcano plots show proteins enriched on AsuHd probes in the upper right quadrant, while proteins in the upper left quadrant were enriched on the lysine controls (Figures 3 and S6). Proteins located in the center of the plots bound both probes with similar efficiency. Cut‐offs were set to −log_10_ *p* > 1.3 and log_2_‐fold enrichment ≥0.6. The most strongly enriched deacetylase on all AsuHd‐containing probes was HDAC6 with exception of the CRTC3 probe (Figures 3a and S6). The latter finding is consistent with unspecific binding of HDAC6 to the CRTC3‐Lys control, resulting in a non‐significant difference of the enrichment ratios (Figure S6c). The log_2_‐fold enrichment of HDAC6 varied between 5.05 (αTub‐probe) and 5.59 (HSP90‐probe) (Figure 3a). Focusing on the αTub‐, HSP90‐, and PPIA‐probes, we analyzed the volcano plots of the interactome profiles in more detail. Notably, the second most enriched HDAC on the AsuHd‐containing probes was HDAC8, with log_2_‐fold enrichment values between 3.07‐ and 4.68‐fold (Figure 3a). This finding could be explained by a model where HDAC8 binds AsuHd‐probes directly and HDAC6 is co‐enriched as an HDAC8 binding partner, or vice versa.

To test this hypothesis, we conducted pulldown experiments with the PPIA‐AsuHd probe and the PPIA‐Lys control and purified recombinant HDACs 1, 6, and 8. The concentrations of the recombinant enzymes were titrated over a range of 0.5–10 nM and probe‐retained proteins were analyzed by western blotting. HDAC6 was efficiently recruited to PPIA‐AsuHd at an input concentration of 0.5 nM, while HDAC8 required a 10‐fold higher enzyme concentration in order to be retained on PPIA‐AsuHd (Figure S7). This observation agrees with the notion that HDAC8 is not the primary binder of the PPIA‐probe.

HDAC8 shares the highest structural homology with the second catalytic domain of HDAC6, which is considered the main deacetylase domain of this enzyme.^[^ ^52^ ^]^ Furthermore, HDAC8 is overexpressed in several cancer cells, including HeLa cells used in this investigation. In HeLa cells, overexpressed HDAC8 was reported to assume redundant functions with HDAC6 by contributing to the removal of the acetyl group of α‐tubulin‐K40, a characteristic substrate of HDAC6.^[^ ^53^ ^]^ This finding encouraged us to analyze if HDAC8 similarly plays a role in regulating acetylation sites used for the design of the HDAC6 probe library. We therefore determined the acetylome of HeLa cells subjected to RNAi knockdown of HDAC8, following a similar approach as described above for the HDAC6 acetylome (Tables S11–S13). We observed 348 acetylation sites in the HDAC8 acetylome, 289 of which overlapped with the acetylation sites of the HDAC6 acetylome (Figure S8). However, none of the acetylation sites used for the design of the HDAC6‐trapping library, including the sequences of the HSP90‐, CRTC3‐, and PPIA‐probes, were enriched upon HDAC8 knockdown. Furthermore, acetylome data of cells treated with HDAC8‐specific inhibitor PCI34051 also identified the corresponding acetylation sites of HSP90 and PPIA, which were not regulated upon inhibitor treatment.^[^ ^46^ ^]^ Collectively, these findings indicate that HDAC8 is not the major deacetylase of the Kac sites used for the probe design in HeLa cells.

After confirming that HDAC6 is a primary binding protein of the AsuHd‐containing probes, we examined the putative secondary binding partners of the αTub‐, HSP90‐, and PPIA‐AsuHd probes, which should also locate to the upper right quadrants of the volcano plots (Figures 3b and S6a,b). Many proteins with acetylation sites upregulated upon HDAC6 knockdown were observed co‐enriched on the αTub‐, HSP90‐, and PPIA‐AsuHd, including proteins that served as templates for the design of the HDAC6‐trapping probes. These proteins included TRIM25, CTTN, and PPIA itself, indicating that HDAC6 associates tightly with these substrates or their deacetylated products. This observation could imply that the interactome analysis might even underestimate HDAC6 interaction with these binding partners due to a competition between endogenous protein substrates and the probe peptides. In general, the co‐enriched proteins could be assigned to seven distinct groups based on their cellular function: Histone‐modifying enzymes, actin‐ and cytoskeleton‐related proteins, chaperones, ubiquitin‐related proteins, transcription regulators, signaling proteins, or cell cycle regulators. As HDAC6 has been implicated as a potential regulator of these cellular functions, Table S14 provides an overview about the roles these proteins play in physiological and pathological processes. Actin and other cytoskeleton‐related proteins were mostly enriched on αTub‐AsuHd and PPIA‐AsuHd, while chaperones were mainly recovered from HSP90‐AsuHd, which might reflect distinct putative roles of HDAC6 in cytoskeletal rearrangement and chaperone regulation, respectively. Cell cycle regulators were mostly enriched on αTub‐AsuHd and PPIA‐AsuHd, while several signaling proteins were eluted from HSP90‐AsuHd. Ubiquitin‐related proteins enriched with αTub‐AsuHd and PPIA‐AsuHd, and to a lesser extent with HSP90‐AsuHd, which could be explained by the ubiquitin‐binding domain of HDAC6. Transcription factors were found to interact with all three AsuHd‐containing probes (Figure 3). We noted that several components of the NF‐κΒ signaling network enriched on the set of AsuHd‐containing probes and continued exploring a potential role of HDAC6 in this pathway.

### Role of HDAC6 in NF‐κΒ Signaling

**Figure 4:**
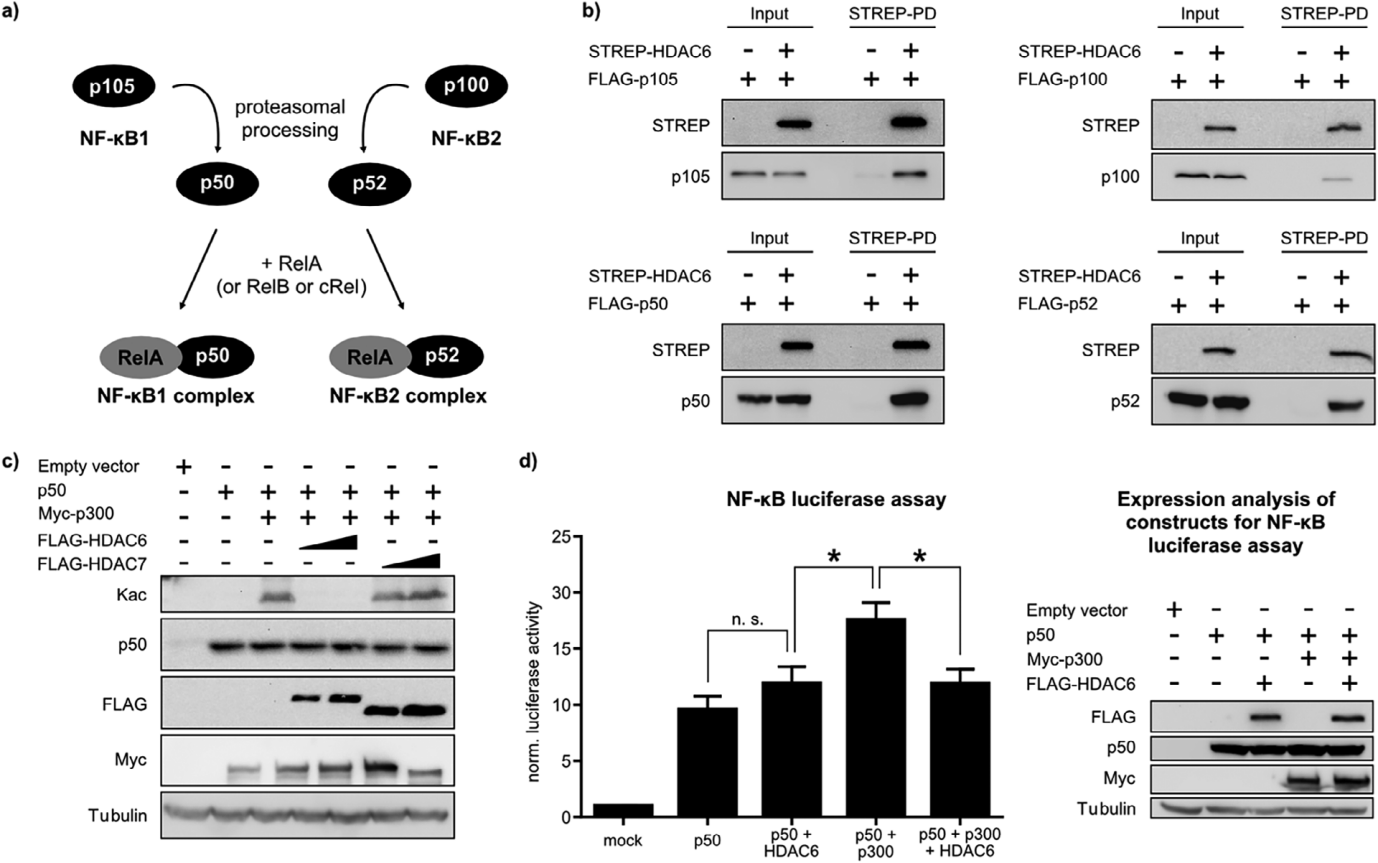
Biochemical investigation of the crosstalk between HDAC6 and NF‐κB signaling: a) Active NF‐κB transcription factors consist of dimers of p50 or p52 in complex with RelA (p65). RelA can be replaced by RelB or cRel. p52 is generated by proteasomal processing of precursor protein p100, both of which are also referred to as NF‐κB2. Likewise, p50 results from partial proteolysis of p105. b) Pulldown (PD) experiments with FLAG‐tagged NF‐κB1 and NF‐κB2 proteins p105, p50, p100, and p52. STREP‐tagged HDAC6 co‐precipitated with all four NF‐κB proteins indicating physical interactions. c) Deacetylation of p50 by HDAC6. Co‐expression of p50 and Myc‐tagged KAT p300 induces p50 acetylation. Expression of HDAC6, but not HDAC7 blocks p50 acetylation indicating that acetyl‐p50 is a substrate of HDAC6. Tubulin servered as loading control d) Luciferase assay for NF‐κB target gene activation. A luciferase reporter construct driven by NF‐κB DNA‐binding sites was co‐expressed with p50, revealing enhanced luciferase activity when compared to the mock control. Co‐expression of p300 and p50 enhanced luciferase activity which was impaired by HDAC6. n. s.: not significant (*p* > 0.05); * significant (*p* ≤ 0.05; p50 versus p50 + p300: *p* = 0.024; p50 + p300 versus p50 + p300 + HDAC6: *p* = 0.018). Error bars indicate standard deviation (± S.D., *n* = 3). Uncropped illustrations of all blots are shown in the Supporting Information. All experiments were performed three times with similar results.

The NF‐κB proteins p100 and RelA (p65) enriched most strongly on PPIA‐AsuHd, but also on αTub‐AsuHd. Furthermore, two inhibitor of NF‐κB kinases (IKKs) were enriched by the HSP90‐AsuHd probe, establishing a potential link between HDAC6 and NF‐κB. Transcription factors of the NF‐κB family play important roles in innate and adaptive immune responses and are frequently dysregulated in autoimmune diseases and cancer.^[^ ^54^, ^55^ ^]^ The NF‐κB family comprises five members: NF‐κB1 and NF‐κB2 which are expressed as 105 kDa (p105) and 100 kDa (p100) precursors that undergo proteasomal processing to the active NF‐κB complex components p50 and p52, respectively. Both proteins possess DNA‐binding domains and form dimers with NF‐κB family members RelA (p65), RelB or c‐Rel that additionally contain a transactivation domain (Figure 4a).^[^ ^56^ ^]^ Of note, RelA was significantly enriched on PPIA‐AsuHd as well. Upon cell stimulation, NF‐κB dimers translocate to the nucleus and induce transcription of NF‐κB target genes. Acetylation of NF‐κB proteins has been observed and analyzed.^[^ ^57^ ^]^ In RelA, acetylated lysine residues were shown to modulate NF‐κB signaling in a site‐specific manner, with HDAC3 uncovered as the main RelA deacetylase.^[^ ^58^ ^]^ The roles of p50 and p52 acetylation are only poorly understood. Three lysine residues (K431, K440, and K441) in p105/p50 are acetylated by KATs p300/CBP, and acetylated p50 showed enhanced DNA‐binding properties in vitro.^[^ ^59^ ^]^ Enhanced p50 binding to NF‐κB‐responsive promotors upon acetylation was further observed in vivo.^[^ ^60^, ^61^ ^]^ Furthermore, a potential role of HDAC6 in modulating NF‐κB‐controlled expression of the H^+^‐K^+^‐ATPase α2 (HKα2), an inducible gene product implicated in neuroinflammation, was proposed.^[^ ^59^, ^62^ ^]^

As our data indicated an interaction of HDAC6 with NF‐κB p100, supporting its potential role as a regulator of NF‐κB, we further investigated this finding in more detail. When analyzing the sequence coverage of p100 enriched on PPIA‐AsuHd, we observed peptides covering the full‐length protein, suggesting that HDAC6 binds either exclusively to full‐length p100, or to both p100 and its processed form p52 (Figure S9). To validate this interaction, we first confirmed the physical interaction of HDAC6 with p100 and p52 and also included p105 and p50 into our analysis. All four proteins were expressed as FLAG‐tag fusions in HEK cells in presence or absence of STREP‐tagged HDAC6. When HDAC6 was pulled‐down on Strep‐Tactin beads, we observed co‐precipitation of all four NF‐κB proteins, indicating a physical interaction of HDAC6 with the full‐length proteins p105 and p100 as well as with their processed active forms p50 and p52 (Figure 4b).

Next, we investigated potential functions of this physical interaction and explored the possibility that NF‐κB proteins might be HDAC6 substrates. To this end, we focused on p50 which was expressed in presence of KAT p300, a known acetyltransferase of the NF‐κB complex. As expected, co‐expression with p300 resulted in p50 acetylation as indicated by p50 staining with anti‐Kac antibodies (Figure 4c). When HDAC6 was co‐expressed with p50 and p300, p50 acetylation was no longer detectable. This effect was observed at high and low HDAC6 expression levels, while HDAC7, serving as control, did not catalyze p50 deacetylation (Figure 4c). These findings indicated that acetylated p50 is a specific substrate of HDAC6. Considering a potential redundant activity of HDAC8 in p50 deacetylation, we performed a side‐by‐side comparison of HDAC8 and HDAC6 (Figure S10). As before, co‐expression of HDAC6 strongly reduced p300‐induced acetylation of p50, whereas overexpression of HDAC8 resulted in only a minor reduction of the acetyl‐p50 signal. This finding indicates that HDAC8 does not play a major role in p50 deacetylation when active HDAC6 is present.

Finally, we explored a functional effect of p300/HDAC6‐controlled p50 acetylation in NF‐κB‐driven target gene expression using a luciferase reporter gene assay. A reporter construct containing the firefly luciferase gene under control of an NF‐κB‐responsive promoter was expressed in presence or absence of p50. As expected, expression of p50 strongly enhanced luciferase activity (Figure 4d). Additional expression of p300 resulted in an even stronger luciferase signal, indicating that p50 acetylation enhanced NF‐κB target gene activation. On the contrary, HDAC6 co‐expression with p50 showed no significant effect on the luciferase activity. However, when HDAC6 was co‐expressed with p300 and p50, the p300‐induced increase of luciferase activity was abolished, suggesting that HDAC6 counteracts the transcriptional activation mediated by p50 acetylation by maintaining p50 in a deacetylated state.

Collectively, these findings indicate that p300‐catalyzed acetylation of p50 enhances the transcriptional activity of NF‐κB. HDAC6, in contrast, attenuates this effect through deacetylation of p50, thereby serving as anti‐inflammatory regulator of NF‐κB signaling. The physiological relevance of p50 and p52 acetylation in NF‐κB signaling needs to be further investigated in the future. However, our results align with the observation that HDAC6 overexpression inhibits NF‐κB‐controlled activation of the HKα2 gene promoter and that p50 acetylation promotes NF‐κB‐controlled transcription of the HIV LTR, thereby facilitating virus replication.^[^ ^59^, ^62^ ^]^

## Conclusion

We developed a versatile strategy for HDAC pulldowns with substrate‐derived HDAC‐trapping peptides in a 96‐well plate format. This approach was enabled by optimizing the building block synthesis of HDAC‐trapping AsuHd, adapting it according to a high‐throughput format and by establishing protocols for parallel synthesis and immobilization of full‐length probe peptides. Pulldown samples of the resulting HDAC‐trapping library were analyzed by western blotting, as demonstrated for 32 acetylation sites translated into high affinity, low affinity and non‐binding control probes. Focusing on HDAC6, we explored four of the preferred probes in more detail and confirmed HDAC6‐catalyzed deacetylation of these sites. Interactome profiling of the selected HDAC‐trapping probes by label‐free quantitative LC‐MS/MS analysis uncovered several known and new HDAC6‐binding proteins. Among those, transcription factor NF‐κB p100 was of particular interest and was further investigated with respect to function and physical interaction with HDAC6. Interaction studies showed HDAC6 binding of NF‐κB p100, its processed form p52 as well as of the homologous proteins p105 and p50. Acetylation of p50 by KAT p300 was shown to enhance NF‐κB target gene transcription, which was reversed by HDAC6. These findings demonstrate that interactome profiling with HDAC‐trapping peptide probes allows uncovering of novel HDAC interaction partners and their functional effects. Importantly, the developed strategy is not limited to HDAC6 or particular probe peptides, but can be adapted for parallel profiling of other HDACs and HDAC complexes. In addition, HDAC‐trapping probes based on full‐length proteins might identify further HDAC binders that require larger surface areas for efficient probe recruitment.

## Supporting Information

The authors have cited additional references within the Supporting Information.^[^ ^63^, ^64^, ^65^, ^66^, ^67^, ^68^, ^69^, ^70^, ^71^, ^72^, ^73^, ^74^, ^75^, ^76^, ^77^, ^78^, ^79^, ^80^, ^81^, ^82^, ^83^, ^84^, ^85^ ^]^

## Conflict of Interests

The authors declare no conflict of interest.

## Supporting information



Supporting Information

Supporting Information

Supporting Information

Supporting Information

## Data Availability

The data that support the findings of this study are available in the Supporting Information of this article.
