# Supplementary material for: Interactome Profiling of a Lysine Deacetylase Trapping Probe Library Uncovers Crosstalk Between HDAC6 and NF‐κB Signaling
Source: Angew Chem Int Ed Engl. 2025 Aug 18;64(40):e202510967. doi: 10.1002/anie.202510967 (PMC12462753; doi:10.1002/anie.202510967)
Supplement: Supplementary file 1 — Supporting Information [file ANIE-64-e202510967-s003.docx]

**Table of contents**

Supporting tables…………......……………………………….. 3

Supporting Table S1-S2, S6-S10, S11-S13………… 3

Supporting Table S3…………………………………… 4

Supporting Table S4…………………………………… 5

Supporting Table S5…….…………………………….. 6

Supporting Table S14…….…………………………….. 7

Supporting schemes...……….………………………………... 9

Supporting Scheme S1….…………………………….. 9

Supporting Scheme S2…………………….………….. 10

Supporting figures…………….………………………...……... 11

Supporting Figure S1………………………………..… 11

Supporting Figure S2………………………………….. 12

Supporting Figure S3………………………………….. 13

Supporting Figure S4………………………………….. 14

Supporting Figure S5………………………………….. 15

Supporting Figure S6………………………………….. 16

Supporting Figure S7………………………………….. 17

Supporting Figure S8………………………………….. 18

Supporting Figure S9………………………………….. 19

Supporting Figure S10………………………………… 20

Experimental procedures………………………………..….... 21

Materials and general methods…………………….... 21

Building block synthesis………………………………. 22

Solid-phase peptide synthesis (SPPS)…………….... 27

Peptide immobilization………………………………... 28

96-Well peptide immobilization………………………. 28

Cell culture……………………………………………… 29

siRNA transfection…………………………………….. 29

Preparation of cell extracts for acetylome analysis… 29

Preparation of cell extracts for pulldown assays….... 29

Pulldown assays……………………………………….. 29

96-Well pulldown assays……………………………… 30

Western blotting………………………………………... 31

Reporter assay of NF-κB target gene activation…… 31

MALDI-MS-based deacetylation assay……………... 31

Filter aided sample preparation (FASP)…………….. 32

In-solution digestion of proteins.................................. 32

On-column dimethyl labeling...................................... 32

Enrichment on acetyllysine antibody agarose............ 33

Nano-LC-MS/MS………………………………………. 33

Data processing and quantification………………….. 34

Data availability………………………………….. ……. 35

Supporting references……………………………………….... 36

NMR spectra and LC-MS data.………………………...……... 38

Supporting Figure S11………………………………… 38

Supporting Figure S12………………………………… 39

Supporting Figure S13………………………………… 40

Supporting Figure S14………………………………… 41

Supporting Figure S15………………………………… 42

Supporting Figure S16………………………………… 43

Supporting Figure S17………………………………… 44

Supporting Figure S18………………………………… 45

Supporting Figure S19………………………………… 46

Supporting Figure S20………………………………… 47

Supporting Figure S21………………………………… 48

Supporting Figure S22………………………………… 49

Supporting Figure S23………………………………… 50

Supporting Figure S24………………………………… 51

Supporting Figure S25………………………………… 52

Supporting Figure S26………………………………… 53

Supporting Figure S27………………………………… 54

Supporting Figure S28………………………………… 55

Supporting Figure S29………………………………… 56

Full-size western blot data.………………………...……........ 57

Supporting Figure S30………………………………… 57

Supporting Figure S31………………………………… 58

Supporting Figure S32………………………………… 59

Supporting Figure S33………………………………… 60

Supporting Figure S34………………………………… 61

**Supporting tables**

Supporting Tables S1-S2, S6-S10 and S11-S13 are provided as three separate spreadsheets.

S1: Proteomics analysis of proteins in HDAC6 knockdown HeLa cells (input)

S2: Acetylome analysis of HDAC6 knockdown HeLa cells vs non-targeted controls

S6: Interactome profiling of mini-l-AsuHd vs mini-Lys

S7: Interactome profiling of αTub-l-AsuHd vs αTub-Lys

S8: Interactome profiling of CRTC-l-AsuHd vs CRTC-Lys

S9: Interactome profiling of HSP90-l-AsuHd vs HSP90-Lys

S10: Interactome profiling of PPIA-l-AsuHd vs PPIA-Lys

S11: Proteomics analysis of proteins in HDAC8 knockdown HeLa cells (input)

S12: Acetylome analysis of HDAC8 knockdown HeLa cells vs non-targeted controls

S13: DIA analysis of proteins in HDAC8 knockdown HeLa cells (input)

**Supporting Table S3:** Amino acid sequences of library probe peptides and parental proteins. Putative substrate sites of HDAC6 are marked in gray. Lit: Sequences derived from existing HDAC-trapping probes or confirmed substrate site,^[1,2]^ RNA_i_: acetylation sites identified in the HDAC6 knockdown acetylome, Tub: acetylation sites identified in the acetylome of cells treated with HDAC6 specific inhibitor tubacin,^[46]^ Cnt: control probes derived from acetylation sites that were not upregulated upon HDAC6 knockdown. CS: cell signaling, CO: cell shape and cytoskeleton organization, TR: transcription and translational regulation, MR: metabolic regulation, PQ: protein folding and quality control, PD: protein degradation, PT: protein transport.

| **Probe name** | **Protein** | **UniProt ID** | **Kac**  **position** | **Sequence** | **Source** | **Protein function** |
| --- | --- | --- | --- | --- | --- | --- |
| mini | - | - | - | GKacG | Lit [12] | - |
| p53 | Cellular tumor antigen p53 | P04637 | 382 | QSTSRHKKacLMFKTEG | Lit [12] | CS |
| αTub | Tubulin alpha-1A chain | Q71U36 | 40 | DGQMPSDKacTIGGGDD | Lit [39] | CO |
| CRTC3 | CREB-regulated transcription coactivator 3 | Q6UUV7-1 | 113 | LHRRSGDKacPGRQFDG | RNA_i_ | TR |
| ELOC | Elongin-C | Q15369 | 32 | DGHEFIVKacREHALTS | RNA_i_ | TR |
| PKM | Pyruvate kinase PKM | P14618-1 | 66 | ETLKEMIKacSGMNVAR | RNA_i_ | MR |
| GPI | Glucose-6-phosphate isomerase | P06744 | 89 | ERMFNGEKacINYTEGR | RNA_i_ | MR |
| GAPDH | Glyceraldehyde-3-phosphate dehydrogenase | P04406-1 | 117 | AHLQGGAKacRVIISAP | RNA_i_ | MR |
| HSP90 | Heat shock protein HSP 90-alpha | P07900-1 | 191 | TKVILHLKacEDQTEYL | RNA_i_ | PQ |
| CANX | Calnexin | P27824-1 | 217 | KTGIYEEKacHAKRPDA | RNA_i_ | PQ |
| CCT5 | T-complex protein 1 subunit epsilon | P48643 | 35 | LMGLEALKacSHIMAAK | RNA_i_ | PQ |
| RANBP2 | E3 SUMO-protein ligase RanBP2 | P49792 | 1851 | NFSEKASKacFGNTEQG | RNA_i_ | PD |
| PPIA | Peptidyl-prolyl cis-trans isomerase A | P62937 | 28 | SFELFADKacVPKTAEN | RNA_i_ | PQ |
| ENAH | Protein enabled homolog | Q8N8S7-1 | 461 | RRRRIAEKacGSTIETE | RNA_i_ | CO |
| PRDX4 | Peroxiredoxin-4 | Q13162 | 78 | DHSLHLSKacAKISKPA | RNA_i_ | CS |
| CTTN (K87) | Src substrate cortactin | Q14247 | 87 | ASHGYGGKacFGVEQDR | RNA_i_ | CO |
| CTTN (K124) | Src substrate cortactin | Q14247 | 124 | SVRGFGGKacFGVQMDR | RNA_i_ | CO |
| PEX5 | Peroxisomal targeting signal 1 receptor | P50542 | 28 | AGHFTQDKacALRQEGL | RNA_i_ | PT |
| MYH9 | Myosin-9 | P35579 | 555 | QEQGTHPKacFQKPKQL | RNA_i_ | CO |
| KPNA6 | Importin subunit alpha-7 | O60684 | 9 | ETMASPGKacDNYRMKS | Tub | PT |
| EIF4B | Eukaryotic translation initiation factor 4B | P23588 | 586 | PEENPASKacFSSASKY | Tub | TR |
| AHNAK | Neuroblast differentiation-associated protein AHNAK | Q09666 | 1177 | SLEGPEGKacLKGPKFK | Tub | CO |
| ZYX | Zyxin | Q15942 | 279 | KFTPVASKacFSPGAPG | Tub | CO |
| PAK1 | Serine/threonine-protein kinase PAK 1 | Q13153-1 | 63 | RSILPGDKacTNKKKEK | Tub | CS |
| NUP124 | Nuclear pore complex protein Nup214 | P35658-1 | 691 | LQPAVAEKacQGHQWKD | Tub | PT |
| TRIM25 | E3 ubiquitin/ISG15 ligase TRIM25 | Q14258 | 402 | PVPALPSKacLPTFGAP | Tub | PD |
| CREBBP | CREB-binding protein | Q92793 | 1595 | KNNKKTNKacNKSSISR | Tub | TR |
| PPL | Periplakin | O60437 | 12 | FRKRNKGKacYSPTVQT | Cnt | CO |
| JADE3 | Protein Jade-3 | Q92613 | 38 | SKIPNEHKacKPAEVFR | Cnt | TR |
| RSF1 | Remodeling and spacing factor 1 | Q96T23-1 | 1061 | GGGVGRGKacDISTITG | Cnt | TR |
| MATR3 | Matrin-3 | P43243 | 473 | VRVHLSQKacYKRIKKP | Cnt | TR |
| CCT2 | T-complex protein 1 subunit beta | P78371 | 50 | LGPKGMDKacILLSSGR | Cnt | PQ |

**Supporting Table S4:** Amino acid sequences of peptide substrates and isotopic standards for MALDI-MS-based deacetylation assay.

| **Substrate name** | **Protein** | **UniProt ID** | **Kac position** | **Sequence** | **Lys(Ac)** | **Lys8** |
| --- | --- | --- | --- | --- | --- | --- |
| mini | - | - | - | GKacG | **P1** | **P6** |
| αTub | Tubulin alpha-1A chain | Q71U36 | 40 | DGQMPSDKacTIGGGDD | **P2** | **P7** |
| CRTC3 | CREB-regulated transcription coactivator 3 | Q6UUV7-1 | 113 | LHRRSGDKacPGRQFDG | **P3** | **P8** |
| HSP90 | Heat shock protein HSP 90-alpha | P07900-1 | 191 | TKVILHLKacEDQTEYL | **P4** | **P9** |
| PPIA | Peptidyl-prolyl cis-trans isomerase A | P62937 | 28 | SFELFADKacVPKTAEN | **P5** | **P10** |

**Supporting Table S5:** Amino acid sequences of probe peptides for interactome analysis.

| **Probe name** | **Protein** | **UniProt ID** | **Kac position** | **Sequence** | **Lys** | **l-AsuHd** |
| --- | --- | --- | --- | --- | --- | --- |
| mini | Mini-probe | - | - | GKacG | **P11** | **P16** |
| αTub | Tubulin alpha-1A chain | Q71U36 | 40 | DGQMPSDKacTIGGGDD | **P12** | **P17** |
| CRTC3 | CREB-regulated transcription coactivator 3 | Q6UUV7-1 | 113 | LHRRSGDKacPGRQFDG | **P13** | **P18** |
| HSP90 | Heat shock protein HSP 90-alpha | P07900-1 | 191 | TKVILHLKacEDQTEYL | **P14** | **P19** |
| PPIA | Peptidyl-prolyl cis-trans isomerase A | P62937 | 28 | SFELFADKacVPKTAEN | **P15** | **P20** |

**Supporting Table S14:** Selected binding proteins detected and quantified for the αTub-AsuHd, HSP90-AsuHd and PPIA-AsuHd probes and their function. Enrichment with statistical significance (p ≤ 0.05 according to LIMMA analysis, three biological replicates) is marked by bold script. Previously identified interactions with HDAC6 are indicated in red in the reference (Ref) column.

| **UniProt ID** | **Protein** | **Gene** | **log_2_-fold change** | | | **Ref** | **Function** |
| --- | --- | --- | --- | --- | --- | --- | --- |
| **Transcription and signal transduction** | |  | αTub | HSP90 | PPIA |  |  |
| Q96EI5 | Transcription elongation factor A protein-like 4 | TCEAL4 | **4.95** | **3.92** | **4.41** | [63] | Transcriptional regulator |
| Q96RE7 | Nucleus accumbens-associated protein 1 | NACC1 | **3.36** | **1.55** | **1.96** | [63] | Transcriptional repressor |
| O15111 | Inhibitor of nuclear factor kappa-B kinase subunit alpha | CHUK | - | **3.18** | - | [63] | Phosphorylates inhibitors of NF-κB, leading to their degradation and NF-κB activation |
| P43246 | DNA mismatch repair protein Msh2 | MSH2 | **2.66** | 1.74 | **1.71** | [64,65] | Component of the post-replicative DNA mismatch repair system |
| O14920 | Inhibitor of nuclear factor kappa-B kinase subunit beta | IKBKB | - | **2.33** | - | [63] | Phosphorylates inhibitors of NF-κB, leading to their degradation and NF-κB activation |
| Q04206 | Transcription factor p65 | RELA | 0.78 | - | **1.96** | [66] | Component of transcription factor NF-κB with roles in innate and adaptive immune response |
| Q00653 | Nuclear factor NF-kappa-B p100 subunit | NFKB2 | 1.32 | -0.31 | **1.89** | [63] | Component of transcription factor NF-κB with roles in innate and adaptive immune response |
| **Actin related and cytoskeleton** | |  |  |  |  |  |  |
| Q9P258 | Protein RCC2 | RCC2 | **5.34** | **2.64** | **4.45** | [67] | Required for assembly of mitotic spindle |
| Q99439 | Calponin-2 | CNN2 | **4.20** | **2.32** | **2.40** | [63] | Binds to actin, calmodulin and tropomyosin and regulates smooth muscle contraction |
| Q16881 | Thioredoxin reductase 1, cytoplasmic | TXNRD1 | 1.74 | 1.07 | **4.07** | [63] | Induces actin and tubulin polymerization |
| Q14247 | Src substrate cortactin | CTTN | **1.11** | -0.02 | **4.03** | [68] | HDAC6 substrate, regulates F-actin polymerization |
| P67775 | Serine/threonine-protein phosphatase 2A catalytic subunit alpha isoform | PPP2CA | 1.99 | **1.49** | **3.98** | [63] | Major phosphatase for microtubule-associated proteins |
| Q9ULV4 | Coronin-1C | CORO1C | **3.85** | 1.81 | **1.91** | [63] | Regulation of actin and microtubule cytoskeleton |
| P58546 | Myotrophin | MTPN | **3.52** | 1.76 | **3.52** | [63] | Inhibits F-actin-capping protein complex formed by CAPZA1 and CAPZB |
| Q5VZK9 | F-actin-uncapping protein LRRC16A | LRRC16A | **3.32** | 0.10 | 1.53 | [63] | Prevents F-actin capping protein activity, enhances actin polymerization |
| P60981 | Destrin | DSTN | **2.24** | -0.69 | **3.06** | [63] | Actin-depolymerizing protein |
| P47755 | F-actin-capping protein subunit alpha-2 | CAPZA2 | 1.88 | 0.67 | **2.78** | [63] | F-actin-capping protein |
| P47756 | F-actin-capping protein subunit beta | CAPZB | **1.84** | 0.15 | 0.91 | [63] | F-actin-capping protein |

| **UniProt ID** | **Protein** | **Gene** | **log_2_-fold change** | | | **Ref** | **Function** |
| --- | --- | --- | --- | --- | --- | --- | --- |
| **Ubiquitin related** | |  | αTub | HSP90 | PPIA |  |  |
| Q93008 | Probable ubiquitin carboxyl-terminal hydrolase FAF-X | USP9X | 2.07 | 0.18 | **4.94** | [63] | Deubiquitinase involved in processing of ubiquitin precursors and ubiquitinylated proteins |
| O14562 | Ubiquitin domain-containing protein UBFD1 | UBFD1 | **4.83** | - | **2.88** | [63] | Binds polyubiquitin, contains ubiquitin-like domain |
| Q04323 | UBX domain-containing protein 1 | UBXN1 | **2.41** | - | **4.08** | [63] | Ubiquitin-binding protein, modulates innate immune response, blocks NF-κB pathway |
| Q14694 | Ubiquitin carboxyl-terminal hydrolase 10 | USP10 | **4.04** | 1.22 | **3.44** | [63] | Hydrolase removing conjugated ubiquitin from target proteins, regulates p53 stability |
| Q8NEZ5 | F-box only protein 22 | FBXO22 | **3.88** | - | **3.65** | [63] | E3 ubiquitin ligase substrate recognition component, promotes degradation of sarcomeric proteins |
| Q14258 | E3 ubiquitin/ISG15 ligase TRIM25 | TRIM25 | **2.78** | 0.50 | **3.25** | [63] | Involved in innate immune defense |
| Q96FW1 | Ubiquitin thioesterase OTUB1 | OTUB1 | 1.63 | 0.49 | **3.02** | [63] | Hydrolase specifically removing Lys-48-linked conjugated ubiquitin from proteins |
| Q06587 | E3 ubiquitin-protein ligase RING1 | RING1 | - | **2.10** | 2.21 | [63] | Mediates monoubiquitinylation of Lys-119 of histone H2A, thereby involved in gene regulation |
| **Chaperones** | |  |  |  |  |  |  |
| Q9HCN8 | Stromal cell-derived factor 2-like protein 1 | SDF2L1 | - | **6.40** | - | [63] | Part of large chaperone multiprotein complex together with DNAJB11 |
| Q9UBS4 | DnaJ homolog subfamily B member 11 | DNAJB11 | - | **5.67** | - | [63] | Part of large chaperone multiprotein complex together with SDF2L1 |
| O43765 | Small glutamine-rich tetratricopeptide repeat-containing protein alpha | SGTA | **-2.22** | **3.46** | -0.24 | [63] | Co-chaperone binding and sorting misfolded proteins |
| Q9UHV9 | Prefoldin subunit 2 | PFDN2 | 2.35 | **1.64** | **3.33** | [63] | Binds to nascent polypeptide chain and promotes folding |
| Q99614 | Tetratricopeptide repeat protein 1 | TTC1 | -0.10 | **2.71** | 1.26 | [63] | Interacts with chaperone HSP90AA |
| Q15813 | Tubulin-specific chaperone E | TBCE | 0.98 | **2.35** | 0.56 | [63] | Tubulin-folding protein, required for correct organization of microtubule cytoskeleton and mitotic splindle |
| **Other proteins** | |  |  |  |  |  |  |
| P09382 | Galectin-1 | LGALS1 | **3.59** | **2.06** | **7.27** | [67] | Lectin binding beta-galactoside with roles in apoptosis, cell proliferation and cell differentiation |
| O43592 | Exportin-T | XPOT | **4.44** | **2.16** | **6.34** | [69] | Mediates the nuclear export of aminoacylated tRNAs |
| Q9BRX2 | Protein pelota homolog | PELO | **4.82** | **3.57** | **4.64** | [64] | Component of the Pelota-HBS1L complex recognizing stalled ribosomes |

**Supporting schemes**

**Supporting Scheme S1:** Synthesis of Fmoc-AsuHd(OTrt)-OH (**5**) and Fmoc-ApmHd(OTrt)-OH (**10**) building blocks. 9-BBN: 9-borabicyclo[3.3.1]nonane, DCM: dichloromethane, THF: tetrahydrofuran, DIPEA: *N,N*-diisopropylethylamine, Fmoc-OSu: Fmoc *N*-hydroxysuccinimide ester.

**Supporting Scheme S2:** Synthesis of the Trt-Mpa-Lys(Dns)-OH (**16**) building block. 9-BBN: 9-borabicyclo[3.3.1]nonane, Dns-Cl: Dansyl chloride, DIPEA: *N,N*-diisopropylethylamine, THF: tetrahydrofuran, TFA: trifluoroacetic acid, DCM: dichloromethane, Trt-Mpa-OSu: *S*-trityl-3-mercaptopropionic acid, OSu: *N*-Hydroxysuccinimide ester.

**Supporting figures**

**Supporting Figure S1:** Probe peptides for 96-well assay. AA: amino acid, mini probes: n = 1, all other probes: n = 7, PEG_2_: 8-amino-3,6-dioxaoctanoic acid, p: d-proline.

**Supporting Figure S2:** Peptide substrates and isotopic standards for MALDI-MS-based deacetylation assay. AA: amino acid, **P1** and **P6**: n = 1, **P2**–**P5** and **P7**–**P10**: n = 7, PEG_2_: 8-amino-3,6-dioxaoctanoic acid, p: d-proline.


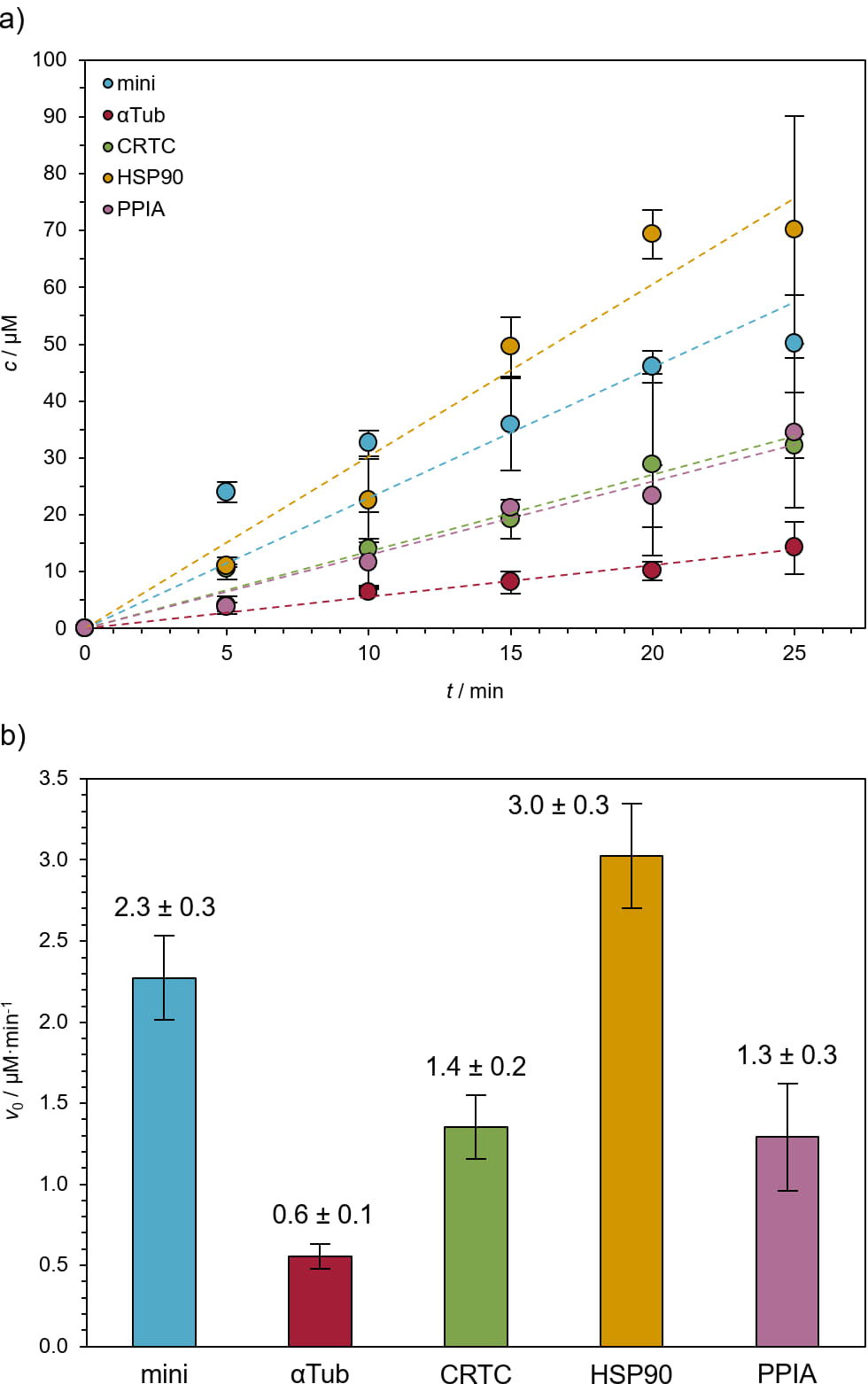


**Supporting Figure S3:** MALDI-MS-based deacetylation assays of HDAC6 were performed with probe sequences containing Kac at the modification sites. Lysine deacetylation was detected by a 42 Da mass shift in the product signal. Isotope-labeled reference peptides were added to the assay mixtures prior to the MS readout and used as reference for quantification. a) Progress curves of HDAC6 deacetylation assays with 100 µM of acetylated substrate peptide and 100 nM recombinant HDAC6. b) Initial velocities determined from progress curves. All of the acetylated peptides were deacetylated by recombinant HDAC6. Deacetylation of mini-Kac proceeded at a rate of 2.3 ± 0.3 µM/min. The HSP90-Kac substrate was deacetylated at an accelerated rate of 3.0 ± 0.3 µM/min, which likely reflected an enhanced interaction between HDAC6 and the HSP90-peptide. The remaining three probes were deacetylated with slower rates. All error bars indicate standard deviation (± S.D., n = 3).

These observations are in line with the notion that the amino acid sequences of the αTub, CRTC3 and PPIA hydroxamate probes do not enhance HDAC6 binding, but repel HDAC1 more strongly than HDAC6. Inspection of all four probes showed that HSP90-AsuHd was the only probe with a hydrophobic leucine residue N-terminal to the acetylation site, while the AsuHd probes of αTub, CRTC3 and PPIA contained an aspartic acid at this position. Furthermore, a reported in-depth profiling showed that acidic residues N-terminal to the acetylation site are absent in HDAC1 substrates.^[16]^ These findings suggest that a negatively charged residue N-terminal to the acetylation site imposes a repulsive effect on the substrate binding of HDAC1, which is well tolerated by HDAC6.

**Supporting Figure S4:** Probe peptides for interactome analysis. AA: amino acid, PEG_3_: 12-amino-4,7,10-trioxadodecanoic acid, Ahx: 6-aminohexanoic acid.


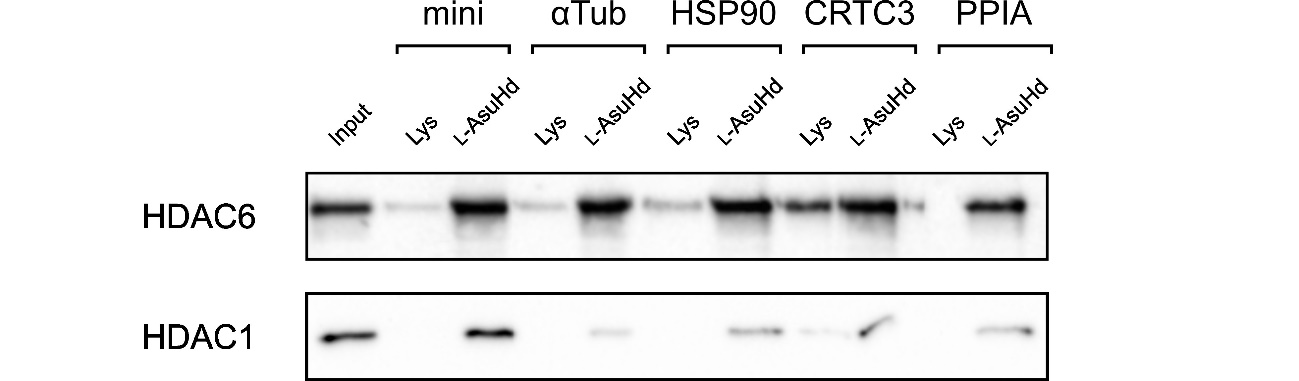


**Supporting Figure S5:** Western blot analysis of pulldown assays with selected probe peptides **P11**–**P20** and HeLa lysate (0.5 mg / mL, 100 µg total protein). Input samples: 16 µg. Uncropped illustrations of all blots in Supporting Figure S5 are shown in the Supporting Figure S33.


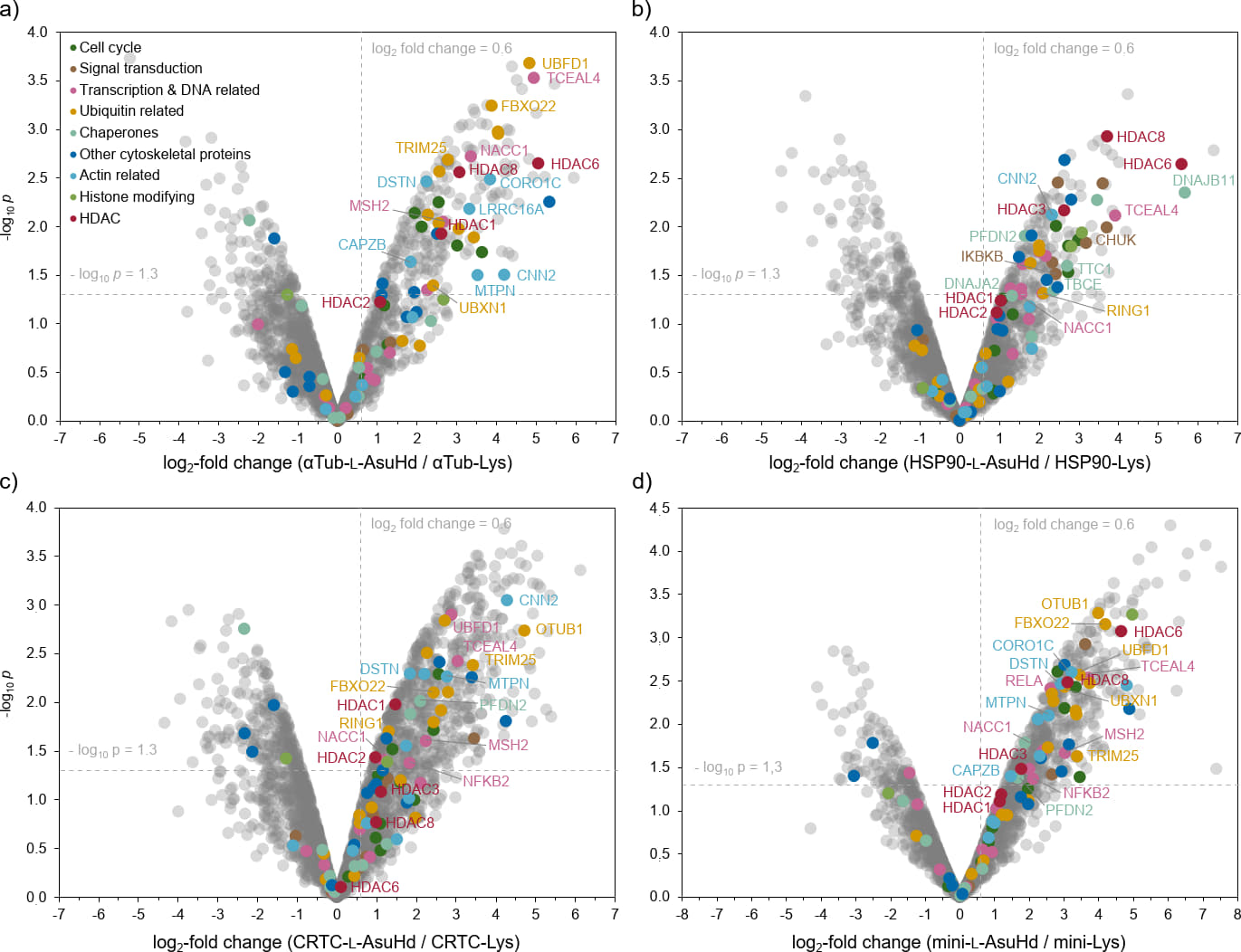


**Supporting Figure S6:** Interactome profiling of the αTub, HSP90, CRTC3 and mini probes. a) Volcano plot of proteins enriched on αTub-l-AsuHd vs αTub-Lys. b) Volcano plot of proteins enriched on HSP90-l-AsuHd vs HSP90-Lys. c) Volcano plot of proteins enriched on CRTC-l-AsuHd vs CRTC-Lys. Under the conditions of proteomic pulldowns with higher protein input HDAC6 shows strong unspecific binding to the CRTC3 sequence indicated by a weak enrichment of HDAC6 on CRTC-AsuHd of only log_2_-fold 0.10. d) Volcano plot of proteins enriched on mini-l-AsuHd vs mini-Lys. HDAC6 is significantly enriched on mini-l-AsuHd with a log_2_-fold change of 4.65.


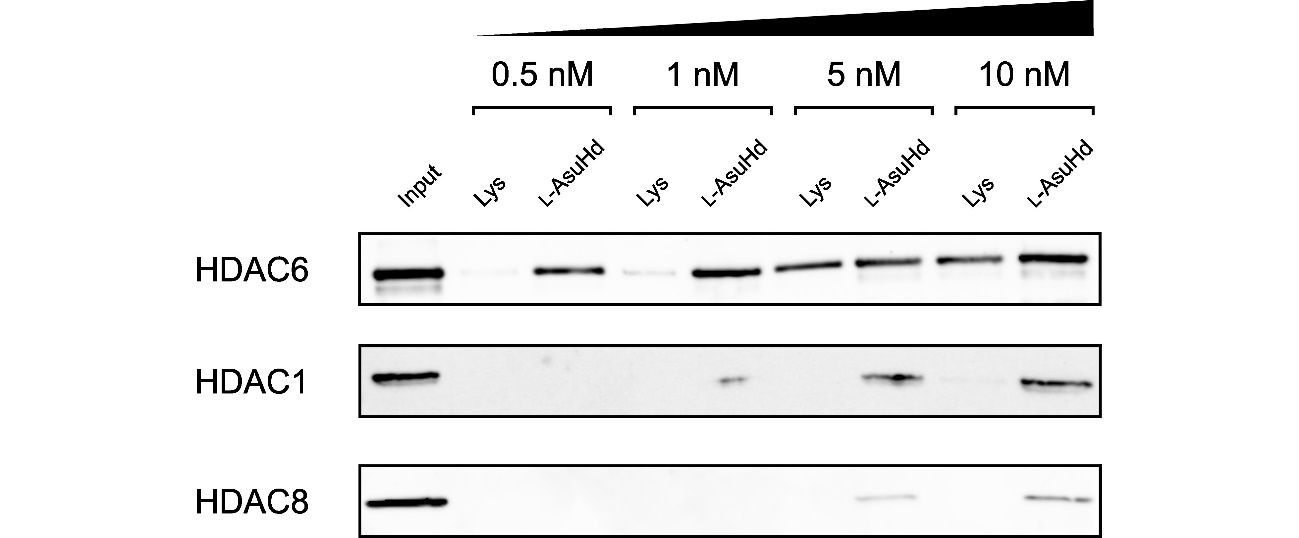


**Supporting Figure S7:** Western blot analysis of pulldown assays with PPIA probe peptides (**P15**, **P20**) and increasing concentrations of recombinant HDAC6, HDAC1 and HDAC8. Input samples: 20 ng. Uncropped illustrations of all blots in Supporting Figure S7 are shown in the Supporting Figure S33.


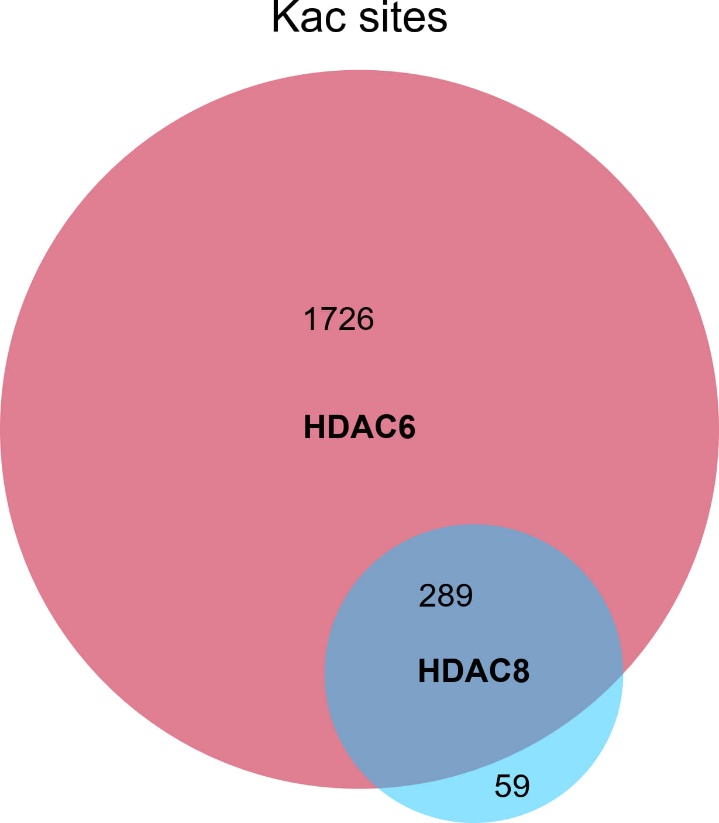


**Supporting Figure S8:** Overlap of lysine acetylation sites (Kac) identified and quantified in Hela cell extracts after knockdown of HDAC6 (red) or HDAC8 (blue). In total, 2015 Kac sites were identified for HDAC6 and 348 Kac sites for HDAC8. Figure created using the BioVenn web application.^[70]^


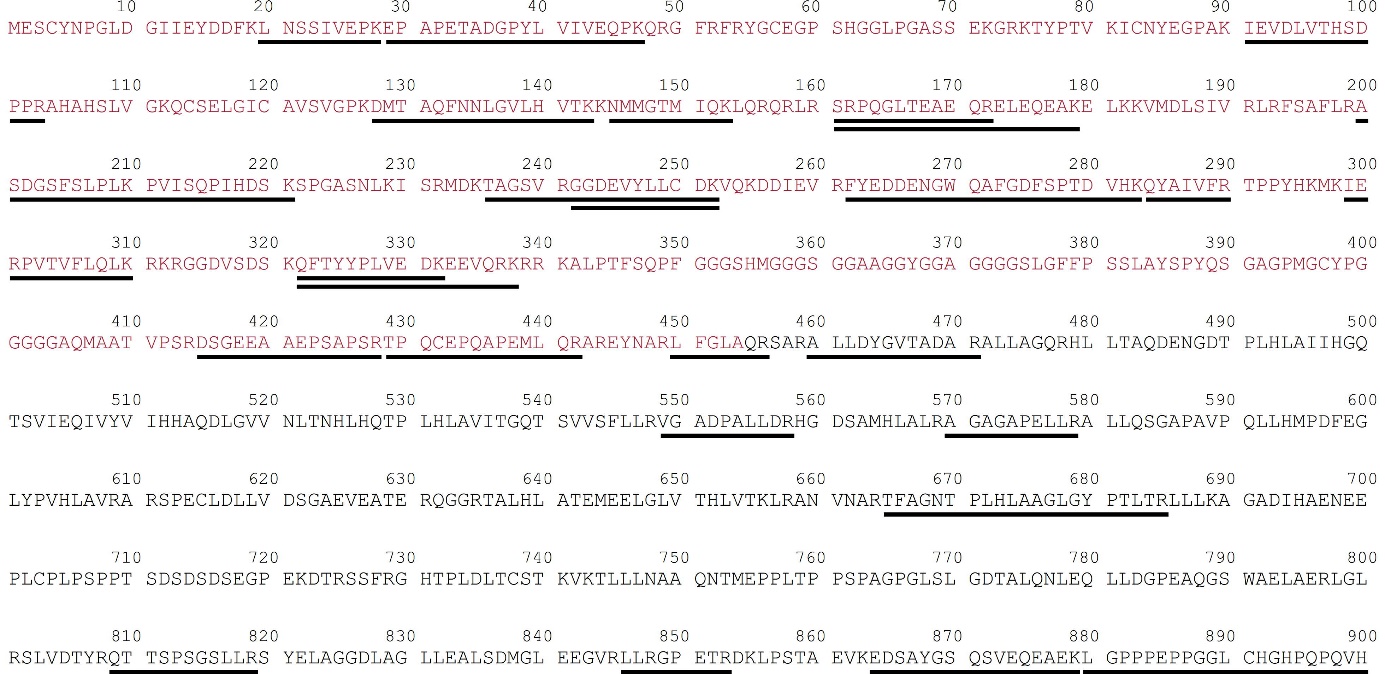


**Supporting Figure S9:** Sequence coverage of tryptic peptides (black bars) identified for p100 (UniProt ID: Q00653). Red characters mark the region of p52 resulting from partial proteolysis of p100.


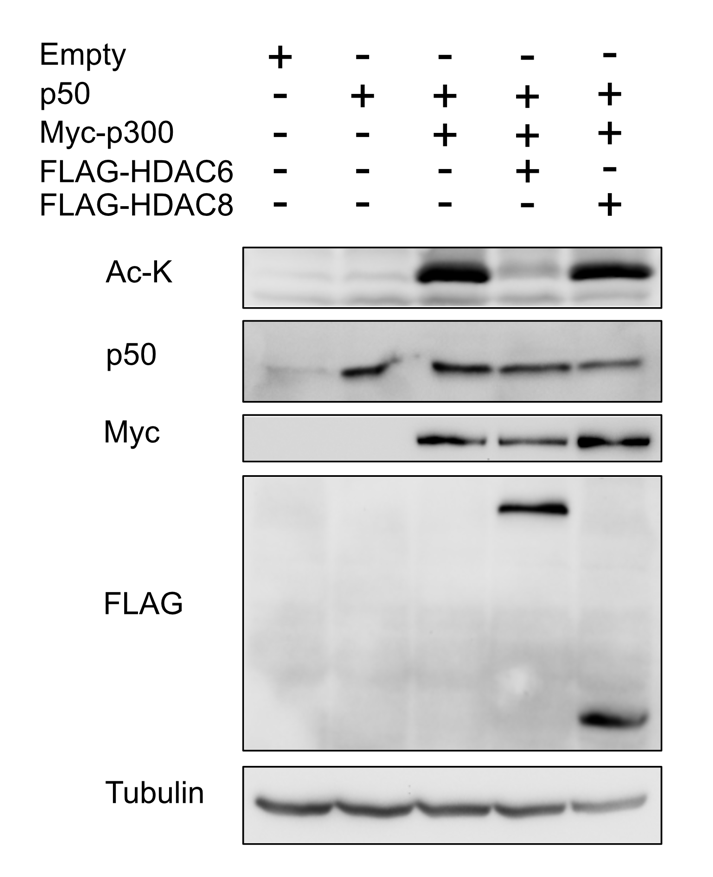


**Supporting Figure S10:** Deacetylation of p50 by HDAC6. Co-expression of p50 and KAT p300 induces p50 acetylation. Expression of HDAC6, but not HDAC8 blocks p50 acetylation indicating that acetyl-p50 is a substrate of HDAC6. Tubulin served as loading control. Uncropped illustrations of all blots in Supporting Figure S10 are shown in the Supporting Figure S34.

**Experimental procedures**

**Materials and general methods**

Amino acid derivatives for solid-phase peptide synthesis and HBTU were purchased from GL Biochem (Shanghai, China), except Fmoc-d-Pro-OH and Fmoc-PEG_2_-OH, which were bought from Fluorochem (Hadfield, UK), and Fmoc-PEG_3_-OH, which was bought from IRIS Biotech (Marktredwitz, Germany). TentaGel HL RAM resin was obtained from Rapp Polymere (Tübingen, Germany). Other chemicals were purchased from Sigma-Aldrich (Steinheim, Germany), Merck (Darmstadt, Germany), Carl Roth (Karlsruhe, Germany), TCI (Eschborn, Germany), Bachem (Bubendorf, Switzerland) or Carbolution (St. Ingbert, Germany). Organic solvents were obtained from J. T. Baker (Deventer, Netherlands), VWR (Leuven, Belgium), Fisher Scientific (Loughborough, UK), Biosolve (Valkenswaard, Netherlands) and Th. Geyer (Renningen, Germany).

Fmoc-Lys8(Boc)-OH (Lys8: ^13^C_6_,^15^N_2_-l-Lysine) was synthesized as reported.^[51]^ Fmoc-l-AsuHd(OTrt)-OH was synthesized as described for Fmoc-AsuHd(OTrt)-OH (**5**) starting from l-2-aminosuberic acid.

Flash chromatography was performed using silica 60 M (0.04–0.063 mm, Macharey-Nagel, Düren, Germany) as stationary phase. Thin layer chromatography (TLC) was performed on TLC silica gel foils with fluorescent indicator obtained from Sigma-Aldrich. Substances were visualized by UV illumination at 254 nm.

Preparative HPLC was performed on a Varian ProStar 210 device equipped with a Reprosil 100 Å 5 μm C18 (250 × 20 mm) column (Dr. Maisch, Ammerbuch, Germany). Crude products were dissolved in HPLC solvents A (0.1% TFA in water) and B (80% MeCN, 0.1% TFA in water) prior to injection. The applied gradient was 5–95% B in 40 min with a flow rate of 13 mL / min. Absorption was detected at 218 nm. Collected fractions were analyzed by LC-MS and lyophilized.

Liquid chromatography-mass spectrometry (LC-MS) was performed using a Shimadzu LC-MS 2020 device (Kyoto, Japan) equipped with a Kinetex 2.6 μm C18 100 Å (100 × 2.1 mm) column (Phenomenex, Aschaffenburg, Germany). Samples were prepared with LC-MS solvents A (0.1% formic acid in water) and B (80% MeCN, 0.1% formic acid in water). The analytical gradient was 5–95% B in 12.75 min or 40–99% B in 12 min with a flow rate of 0.2 mL / min. Absorption was detected at 218 nm and 340 nm. The ESI-MS was operated in positive mode.

Matrix-assisted laser desorption / ionization (MALDI) mass spectrometry was performed on a Bruker Reflex IV MALDI-TOF device in reflection mode. α-Cyano-4-hydroxycinnamic acid (CHCA, 5 mg / mL in 50% MeCN, 0.1% TFA in water) or 2,5-dihydroxybenzoic acid (DHB, 20 mg / mL in 50% MeCN, 0.1% TFA in water) were used as matrices. Samples and matrix solutions were spotted in equal amounts onto a polished steel target. Spots were measured with at least 200 shots with laser intensity between 20% and 50%. Recorded mass spectra were baseline-corrected and smoothed with three runs of the Savitzky-Golay filter (window size 0.3 *m* / *z*) prior to peak analysis using mMass 5.5.0.^[71]^

NMR spectra were recorded on a Bruker Advance III HD 300 XWB (Billercia, USA) or Bruker Advance III HDX 400 spectrometer. The ^1^H and ^13^C chemical shifts were reported in ppm relative to the tetramethylsilane reference. Further assignments of ^1^H resonances were performed on basis of ^1^H-^1^H-COSY spectra. ^13^C resonances were assigned by ^13^C-DEPT-135, ^1^H-^13^C-HSQC and ^1^H-^13^C-HMBC experiments. ^1^H spectra were referenced to the residual proton signal of deuterated solvents (HDO: *δ* / ppm = 4.75, DMSO-d_5_: *δ* / ppm = 2.50, MeCN-d_2_: *δ* / ppm = 1.94). ^13^C spectra were referenced to the ^13^C signal of the respective solvents (DMSO-d_6_: *δ* / ppm = 39.5; MeCN-d_3_: *δ* / ppm = 1.4, 118.7).

**Building block synthesis**

**Asu-BBN (2)**

Under argon atmosphere dl-2-aminosuberic acid (**1**) (1.89 g, 10 mmol, 1 eq) was suspended in anhydrous MeOH (60 mL) and heated at reflux. Subsequently, 9-BBN (0.5 M in THF, 22 mL, 11 mmol, 1.1 eq) was added dropwise, and the mixture was heated until the suspension had cleared (approximately 2 h). The solvent was removed under reduced pressure and the colorless, viscous residue purified by preparative HPLC. The desired product **2** was obtained as a white powder (2.81 g, 9.09 mmol, 91%).

HPLC (5–95% B in 12.75 min, 0.2 mL / min): *t*_ret._ = 11.58 min.

ESI-MS: [M+H]^+^, *m* / *z* = 310.2 (calculated), 310.1 (found); [M+Na]^+^, *m* / *z* = 332.2 (calculated), 332.1 (found).

^1^H-NMR (400 MHz, DMSO-d_6_): *δ* / ppm = 0.46 (s, 1 H, CH_BBN_), 0.51 (s, 1 H, CH_BBN_), 1.22–1.86 (m, 20 H; 6×CH_2BBN_, 4×CH_2Asu_), 2.21 (t, ^3^*J*_HH_ = 7.3 Hz, 2 H, ζ-CH_2_), 3.41–3.53 (m, 1 H, α-CH), 5.68–5.86 (m, 1 H, NH_2_), 6.30–6.48 (m, 1 H, NH_2_), 11.96 (br s, 1 H, CO_2_H).

^13^C{^1^H}-NMR (101 MHz, DMSO-d_6_): *δ* / ppm = 1.2, 22.3 (CH_BBN_), 23.5 (CH_BBN_), 23.9, 24.3 (ε-CH_2_), 25.3, 28.2, 30.3, 30.7, 30.7, 31.2, 31.3, 33.5 (ζ-CH_2_), 54.4 (α-CH), 173.7 (α-CO_2_), 174.5 (ζ-CO_2_).

**Apm-BBN (7)**

Apm-BBN (**7**) was synthesized as described for Asu-BBN (**2**), but using dl-2-aminopimelic acid (**6**) (1.41 g, 8.07 mmol, 1 eq), anhydrous MeOH (45 mL), and 9-BBN (0.5 M in THF, 18 mL, 8.9 mmol, 1.1 eq). Product **7** was obtained as a white powder (1.93 g, 6.53 mmol, 81%).

HPLC (5–95% B in 12.75 min, 0.2 mL / min): *t*_ret._ = 11.45 min.

ESI-MS: [M+H]^+^, *m* / *z* = 296.2 (calculated), 296.0 (found); [M+Na]^+^, *m* / *z* = 318.2 (calculated), 318.0 (found).

^1^H-NMR (400 MHz, DMSO-d_6_): *δ* / ppm = 0.46 (s, 1 H, CH_BBN_), 0.51 (s, 1 H, CH_BBN_), 1.33–1.84 (m, 18 H, 6×CH_2BBN_, 3×CH_2Apm_), 2.22 (t, ^3^*J*_HH_ = 6.9 Hz, 2 H, ε-CH_2_), 3.40–3.55 (m, 1 H, α-CH), 5.69–5.86 (m, 1 H, NH_2_), 6.31–6.48 (m, 1 H, NH_2_).

^13^C{^1^H}-NMR (101 MHz, DMSO-d_6_): δ / ppm = 22.3 (CH_BBN_), 23.4 (CH_BBN_), 23.9, 24.2, 24.6 (δ-CH_2_), 25.2, 30.2, 30.7, 31.2, 31.3, 33.6 (ε-CH_2_), 54.4 (α-CH), 173.7 (α-CO2), 174.4 (ε-CO_2_).

**AsuHd(OTrt)-BBN (3)**

Crude Asu-BBN (**2**) (corresponding to 5.00 mmol of **1**) was dissolved in anhydrous DCM / THF (1:1, 100 mL) under argon atmosphere. After addition of pyridine (0.41 mL, 5.00 mmol, 1 eq) and SOCl_2_ (0.37 mL, 5.00 mmol, 1 eq) the reaction mixture was stirred at room temperature for 1 h. The mixture was cooled in an ice bath and a solution of Trt-ONH_2_ (1.38 g, 5.00 mmol, 1 eq) and DIPEA (2.62 mL, 15.0 mmol, 3 eq) in anhydrous DCM / THF (1:1, 25 mL) was added dropwise. The mixture was brought to room temperature overnight while stirring, and then concentrated under reduced pressure to yield crude product **3**, which was used in the next step without further purification.

HPLC (40–99% B in 12.00 min, 0.2 mL / min): *t*_ret._ = 15.48 min.

ESI-MS: [M+Na]^+^, *m* / *z* = 589.3 (calculated), 589.5 (found).

**ApmHd(OTrt)-BBN (8)**

ApmHd(OTrt)-BBN (**8**) was synthesized as described for AsuHd(OTrt)-BBN (**3**), but using HPLC-purified Apm-BBN (**7**) (1.48 g, 5.00 mmol, 1 eq) in anhydrous DCM / THF (1:1, 100 mL), pyridine (0.41 mL, 5.00 mmol, 1 eq), and SOCl_2_ (0.37 mL, 5.00 mmol, 1 eq). Trt-ONH_2_ (1.38 g, 5.00 mmol, 1 eq) and DIPEA (2.62 mL, 15.0 mmol, 3 eq) were dissolved in anhydrous DCM / THF (1:1, 25 mL) and crude product **8** was used in the next step without further purification.

HPLC (40–99% B in 12.00 min, 0.2 mL / min): *t*_ret._ = 15.23 min.

ESI-MS: [M+Na]^+^, *m* / *z* = 575.3 (calculated), 575.3 (found).

**H-AsuHd(OTrt)-OH (4)**

Crude AsuHd(OTrt)-BBN (**3**) (corresponding to 5.00 mmol of **1**) was dissolved in THF (50 mL), ethylenediamine (1.67 mL, 25.0 mmol, 5 eq) was added, and the mixture was heated for 1 min below the boiling point. The precipitate was collected by centrifugation and dissolved in MeCN / H_2_O. The pH was adjusted to 4 with aqueous HCl and the crude product purified by preparative HPLC. The fractions were neutralized with aqueous NaOH, lyophilized, and the desired product **4** was obtained as white, powdery solid (1.36 g, 3.04 mmol, 61% with respect to **1**).

HPLC (40–99% B in 12.00 min, 0.2 mL / min): *t*_ret._ = 7.21 min.

ESI-MS: [M+H]^+^, *m* / *z* = 477.3 (calculated), 477.2 (found); [M+Na]^+^, *m* / *z* = 469.2 (calculated), 469.3 (found).

^1^H-NMR (400 MHz, MeCN-d_3_ / D_2_O (3:1)): *δ* / ppm = 1.49–1.64 (m, 2 H, δ-CH_2_), 1.68–1.85 (m, 4 H, γ/ε-CH_2_), 1.98–2.19 (m, 2 H, β-CH_2_), 2.27–2.42 (m, 2 H, ζ-CH_2_), 3.74–3.91 (m, 1 H, α-CH), 7.76–8.06 (m, 15 H, CH_Trt_).

^13^C{^1^H}-NMR (101 MHz, MeCN-d_3_ / D_2_O (3:1)): *δ* / ppm = 25.7 (γ/ε-CH_2_), 25.7 (γ/ε-CH_2_), 29.3 (δ-CH_2_), 33.3 (ζ-CH_2_), 33.5 (β-CH_2_), 56.4 (α-CH_2_), 93.8* (CPh_3_), 119.1 (CH_Trt_), 128.9 (CH_Trt_), 130.0 (CH_Trt_), 143.3 (*C*CH_Trt_), 173.2* (ζ-CO), 178.6* (α-CO_2_).

Values marked with an asterisk were determined from the respective ^1^H-^13^C-HMBC-NMR spectrum.

**H-ApmHd(OTrt)-OH (9)**

H-ApmHd(OTrt)-OH (**9**) was synthesized as described for H-AsuHd(OTrt)-OH (**4**), but using crude ApmHd(OTrt)-BBN (**8**) (corresponding to 5.00 mmol of **7**), THF (50 mL), and ethylenediamine (1.67 mL, 25.0 mmol, 5 eq), resulting in product **8** as white, powdery solid (1.61 g, 3.73 mmol, 75% with respect to **7**).

HPLC (40–99% B in 12.00 min, 0.2 mL / min): *t*_ret._ = 6.51 min.

ESI-MS: [M+H]^+^, *m* / *z* = 433.2 (calculated), 433.1 (found); [M+Na]^+^, *m* / *z* = 455.2 (calculated), 455.0 (found).

^1^H-NMR (400 MHz, MeCN-d_3_ / D_2_O (1:1)): *δ* / ppm = 1.38–1.52 (m, 2 H, γ-CH_2_), 1.52–1.64 (m, 2 H, δ-CH_2_), 1.83–2.02 (m, 2 H, β-CH_2_), 2.19 (t, ^3^J_HH_ = 7.6 Hz, 2 H, ε-CH_2_), 3.69–3.76 (m, 1 H, α-CH), 7.43–7.97 (m, 15 H, CH_Trt_).

^13^C{^1^H}-NMR (101 MHz, MeCN-d_3_ / D_2_O (1:1)): *δ* / ppm = 25.3 (γ-CH_2_), 25.6 (δ-CH_2_), 32.4 (β-CH_2_), 33.0 (ε-CH_2_), 56.0 (α-CH_2_), 94.0 (CPh_3_), 119.7 (CH_Trt_), 128.9 (CH_Trt_), 129.8 (CH_Trt_), 142.8 (*C*CH_Trt_), 173.2 (ε-CO), 177.7 (α-CO_2_).

**Fmoc-AsuHd(OTrt)-OH (5)**

H-AsuHd(OTrt)-OH (**4**) (1.36 g, 3.04 mmol, 1 eq) and NaHCO_3_ (0.74 g, 9.12 mmol, 3 eq) were dissolved in dioxane / H_2_O (60 mL), and Fmoc-OSu (0.51 g, 1.52 mmol, 0.5 eq), dissolved in dioxane (6 mL), was added. The mixture was stirred for 1 h at room temperature and the reaction monitored via LC-MS. Further Fmoc-OSu, dissolved in dioxane, was added in portions of 0.1 eq (to prevent Fmoc-β-alanine formation by excess reagent) until full conversion was achieved. The reaction mixture was extracted with EtOAc (3×30 mL), the pooled organic phases dried over Na_2_SO_4_, and then concentrated under reduced pressure to yield the desired product **5** as colorless solid (1.61 g, 2.41 mmol, 79%).

HPLC (40–99% B in 12.00 min, 0.2 mL / min): *t*_ret._ = 15.23 min.

ESI-MS: [M+H]^+^, *m* / *z* = 669.3 (calculated), 669.3 (found); [M+Na]^+^, *m* / *z* = 691.3 (calculated), 691.4 (found).

^1^H-NMR (400 MHz, DMSO-d_6_ / 200 mM Na_2_HPO_3_ in D_2_O, pH 7 (9:1)): *δ* / ppm = 0.79–0.97 (m, 2 H, δ-CH_2_), 0.99–1.21 (m, 4 H, γ/ε-CH_2_), 1.37–1.61 (m, 2 H, β-CH_2_), 1.64–1.82 (m, 2 H, ζ-CH_2_), 3.64–3.68 (m, 1 H, α-CH), 4.15–4.29 (m, 3 H, C*H*CH_2_ / CHC*H*_2Fmoc_), 7.21–7.35 (m, 17 H, 15×CH_Trt_, 2×CH_Fmoc_), 7.35–7.42 (m, 2 H, CH_Fmoc_), 7.62–7.70 (m, 2 H, CH_Fmoc_), 7.80–7.88 (m, 2 H, CH_Fmoc_).

^13^C{^1^H}-NMR (101 MHz, DMSO-d_6_ / 200 mM Na_2_HPO_3_ in D_2_O, pH 7 (9:1)): *δ* / ppm = 25.2 (γ/ε-CH_2_), 25.2 (γ/ε-CH_2_), 28.7 (δ-CH_2_), 32.3 (β-CH_2_), 32.4 (ζ-CH_2_), 47.1 (*C*HCH_2Fmoc_), 55.5 (α-CH), 65.8 (CH*C*H_2Fmoc_), 92.2 (CPh_3_), 120.5 (CH_Fmoc_), 125.6 (CH_Fmoc_), 127.5 (CH_Fmoc_), 127.9 (2×CH_Trt_), 128.1 (CH_Fmoc_), 129.3 (CH_Trt_), 141.0 (C_qFmoc_), 142.8 (*C*CH_Trt_), 144.1 (C_qFmoc_), 155.9 (CONH_Fmoc_), 171.0 (ζ-CO), 174.6 (α-CO_2_).

**Fmoc-ApmHd(OTrt)-OH (10)**

Fmoc-ApmHd(OTrt)-OH (**10**) was synthesized as described for Fmoc-AsuHd(OTrt)-OH (**5**), but using H-ApmHd(OTrt)-OH (**9**) (1.61 g, 3.73 mmol, 1 eq), NaHCO_3_ (0.78 g, 9.28 mmol, 2.5 eq), dioxane / H_2_O (40 mL), and Fmoc-OSu (0.63 g, 1.86 mmol, 0.5 eq), dissolved in dioxane (4 mL). The desired product **10** was obtained as colorless solid (1.83 g, 2.80 mmol, 75%).

HPLC (40–99% B in 12.00 min, 0.2 mL / min): *t*_ret._ = 14.37 min.

ESI-MS: [M+H]^+^, *m* / *z* = 677.3 (calculated), 677.1 (found).

^1^H-NMR (400 MHz, DMSO-d_6_ / 200 mM Na_2_HPO_3_ in D_2_O, pH 7 (9:1)): *δ* / ppm = 0.88–1.04 (m, 2 H, γ-CH_2_), 1.06–1.18 (m, 2 H, δ-CH_2_), 1.33–1.60 (m, 2 H, β-CH_2_), 1.64–1.80 (m, 2 H, ε-CH_2_), 3.58–3.65 (m, 1 H, α-CH), 4.15–4.28 (m, 3 H, C*H*CH_2_ / CHC*H*_2Fmoc_), 7.23–7.33 (m, 17 H, 15×CH_Trt_, 2×CH_Fmoc_), 7.33–7.41 (m, 2 H, CH_Fmoc_), 7.61–7.69 (m, 2 H, CH_Fmoc_), 7.79–7.88 (m, 2 H, CH_Fmoc_).

^13^C{^1^H}-NMR (101 MHz, DMSO-d_6_ / 200 mM Na_2_HPO_3_ in D_2_O, pH 7 (9:1)): *δ* / ppm = 25.1 (γ-CH_2_), 25.2 (δ -CH_2_), 32.5 (β/ε-CH_2_), 47.1 (*C*HCH_2Fmoc_), 55.7 (α-CH), 65.8 (CH*C*H_2Fmoc_), 92.3 (CPh_3_), 120.5 (CH_Fmoc_), 125.6 (CH_Fmoc_), 127.5 (CH_Fmoc_), 127.9 (2×CH_Trt_), 128.1 (CH_Fmoc_), 129.3 (CH_Trt_), 141.0 (C_qFmoc_), 142.7 (*C*CH_Trt_), 144.1 (C_qFmoc_), 155.8 (CONH_Fmoc_), 171.0 (ε-CO), 174.8 (α-CO_2_).

**Lys-BBN (12)**

Lys-BBN (**12**) was synthesized as described for Asu-BBN (**2**), but using l-lysine·HCl (**11**) (1.83 g, 10 mmol, 1 eq), anhydrous MeOH (60 mL), and 9-BBN (0.5 M in THF, 22 mL, 11 mmol, 1.1 eq). Crude product **12** was obtained as a colorless, viscous residue, which was used in the next step without further purification.

HPLC (5–95% B in 12.75 min, 0.2 mL / min): *t*_ret._ = 7.86 min.

ESI-MS: [M+H]^+^, *m* / *z* = 267.2 (calculated), 267.2 (found); [M+Na]^+^, *m* / *z* = 289.2 (calculated), 289.2 (found).

^1^H-NMR (300 MHz, DMSO-d_6_): *δ* / ppm = 0.46 (s, 1 H, CH_BBN_), 0.51 (s, 1 H, CH_BBN_), 1.16– 1.92 (m, 18 H, 6×CH_2BBN_, 3×CH_2Lys_), 2.68–2.89 (m, 2 H, ε-CH_2_), 3.41–3.59 (m, 1 H, α-CH), 5.69–5.96 (m, 1 H, NH_2_), 6.35–6.64 (m, 1 H, NH_2_), 7.87 (br s, 3 H, ε-NH_3_^+^).

^13^C{^1^H}-NMR (75 MHz, DMSO-d_6_): δ / ppm = 22.4, 23.4 (CH_BBN_), 23.5 (CH_BBN_), 23.9, 24.3, 26.6, 29.7, 29.7, 30.7, 31.2, 31.3, 38.6 (ε-CH_2_), 54.2 (α-CH), 173.7 (α-CO_2_).

**Lys(Dns)-BBN (13)**

Crude Lys-BBN (**12**) (corresponding to 10.0 mmol of **11**) was dissolved in anhydrous THF (100 mL) and DIPEA (4.37 mL, 25.0 mmol, 2.5 eq) was added, followed by Dns-Cl (2.70 g, 10.0 mmol, 1 eq). The mixture was stirred overnight at room temperature and then concentrated under reduced pressure. Crude product **13** was used in the next step without further purification.

HPLC (40–99% B in 12.00 min, 0.2 mL / min): *t*_ret._ = 13.41 min.

ESI-MS: [M+H]^+^, *m* / *z* = 500.3 (calculated), 500.2 (found); [M+Na]^+^, *m* / *z* = 522.3 (calculated), 522.2 (found).

**H-Lys(Dns)-OH (14)**

Crude Lys(Dns)-BBN (**13**) (corresponding to 10.0 mmol of **11**) was dissolved in TFA (50 mL) and stirred at 50 °C for 4 h. The reaction was monitored by LC-MS and further TFA was added until near to full conversion was achieved. The mixture was concentrated under reduced pressure and the residue diluted with MeCN / H_2_O, and lyophilized. Crude product **14** was used in the next step without further purification. A sample of **14** was purified by preparative HPLC in order to obtain analytical data.

HPLC (40–99% B in 12.00 min, 0.2 mL / min): *t*_ret._ = 5.24 min.

ESI-MS: [M+H]^+^, *m* / *z* = 380.2 (calculated), 380.2 (found).

^1^H-NMR (400 MHz, DMSO-d_6_): *δ* / ppm = 1.21–1.43 (m, 4 H, 4/5-H), 1.59–1.72 (m, 2 H, 3-H), 2.70–2.80 (m, 2 H, 6-H), 2.83 (s, 6 H, CH3), 3.76–3.85 (m, 1 H, 2-H), 7.23–7.30 (m, 1 H, 13-H), 7.55–7.67 (m, 2 H, 9/14-H), 7.90 (t, ^3^J_HH_ = 5.7 Hz, 1 H, NH), 8.06–8.12 (m, 1 H, 8-H), 8.23 (br s, 3 H, NH_3_^+^), 8.28–8.34 (m, 1 H, 15-H), 8.43–8.49 (m, 1 H, 10-H).

^13^C{^1^H}-NMR (101 MHz, DMSO-d_6_): δ / ppm = 21.5 (C-4/5), 28.7 (C-4/5), 29.5 (C-3), 42.1 (C-6), 45.1 (C-17), 51.8 (C-2), 115.2 (C-13), 119.2 (C-15), 123.6 (C-14), 127.8 (C-9), 128.2 (C-8), 129.1 (C-11/16), 129.1 (C-11/16), 129.3 (C-10), 136.0 (C-7), 151.2 (C-12), 171.0 (C-1).

**Trt-Mpa-OSu (15)**

Under argon atmosphere *S*-trityl-3-mercaptopropiocic acid (Trt-Mpa-OH) (1.74 g, 5.00 mmol, 1 eq), *N*-hydroxysuccinimide (1.15 g, 10.0 mmol, 2 eq) and 1-ethyl-3-(3-dimethylaminopropyl)carbodiimide hydrochloride (EDC·HCl) (1.15 g, 6.00 mmol, 1.2 eq) were suspended in anhydrous DCM / THF (1:1, 50 mL). DIPEA (2.62 g, 15.0 mmol, 3 eq) was added and the reaction mixture was stirred at room temperature overnight. The mixture was concentrated under reduced pressure and the residue was taken up in EtOAc, and washed with aqueous HCl (0.3 M, 3×). The organic phase was dried over Na_2_SO_4_ and the solvent removed *in vacuo* to yield crude product **15** as white solid, which was used in the next step without further purification.

HPLC (40–99% B in 12.00 min, 0.2 mL / min): *t*_ret._ = 15.29 min.

ESI-MS: [M+Na]^+^, *m* / *z* = 468.1 (calculated), 468.1 (found); [M+K]^+^, *m* / *z* = 484.1 (calculated), 484.0 (found).

**Trt-Mpa-Lys(Dns)-OH (16)**

Crude H-Lys(Dns)-OH (**14**) (corresponding to 10.0 mmol of **11**) was dissolved in anhydrous DCM (50 mL). DIPEA (2.62 mL, 15.0 mmol, 3 eq) was added and the pH adjusted to 9 with additional DIPEA. Trt-Mpa-OSu (**15**) (corresponding to 3.08 mmol, 0.34 eq of Trt-Mpa-OH), dissolved in DCM (20 mL), was added, and the reaction mixture was stirred overnight at room temperature. The solvent was removed under reduced pressure and the residue diluted with H_2_O (100 mL). The pH was adjusted to 2–3 with saturated, aqueous KHSO_4_ and the mixture extracted with Et_2_O (3×50 mL) The pooled organic phases were dried over Na_2_SO_4_ and the solvent removed *in vacuo*. The crude product was purified by flash chromatography with silica gel as stationary and MeOH / DCM (1:20) with formic acid (0.1%) as mobile phase, after which the desired product **16** was obtained as yellow oil (1.79 g, 2.52 mmol, 82% with respect to Trt-Mpa-OH).

TLC (MeOH / DCM (1:20), 0.1% formic acid): *R*_f._ = 0.18.

HPLC (40–99% B in 12.00 min, 0.2 mL / min): *t*_ret._ = 15.07 min.

ESI-MS: [M+H]^+^, *m* / *z* = 710.3 (calculated), 710.3 (found); [M+Na]^+^, *m* / *z* = 732.3 (calculated), 732.2 (found).

^1^H-NMR (400 MHz, DMSO-d_6_): *δ* / ppm = 1.09–1.80 (m, 8 H, 3/4/5/6-H), 2.15–2.25 (m, 4 H, 19/20-H), 2.68–2.76 (m, 2 H, 6-H), 2.81 (s, 6 H, 17-H), 3.97–4.06 (m, 1 H, 2-H), 7.17–7.26 (m, 4 H, 13/25-H), 7.26–7.34 (m, 12 H, 13/24-H), 7.54–7.63 (m, 2 H, 9/14-H), 7.87 (t, ^3^J_HH_ = 5.7 Hz, 1 H, ε-NH), 7.97–8.03 (d, ^3^J_HH_ = 7.8 Hz, 1 H, α-NH) 8.05–8.10 (m, 1 H, 8-H), 8.27–8.33 (m, 1 H, 15-H), 8.41–8.47 (m, 1 H, 10-H).

^13^C{^1^H}-NMR (101 MHz, DMSO-d_6_): δ / ppm = 22.5 (C-4/5), 27.4 (C-19/20), 28.8 (C-4/5), 30.5 (C-3), 33.6 (C-19/20), 42.2 (C-6), 45.0 (C-17), 51.6 (C-2), 65.9 (C-21), 115.1 (C-13), 119.1 (C-15), 123.5 (C-14), 126.6 (C-25), 127.8 (C-9), 128.0 (C-23/24), 128.1 (C-8), 129.0 (C-23/24), 129.1 (C-11/16), 129.1 (C-11/16), 129.3 (C-10), 136.1 (C-7), 144.4 (C-22), 151.3 (C-12), 170.1 (C-18), 173.5 (C-1).

**Solid-phase peptide synthesis (SPPS)**

Peptides were synthesized applying the Fmoc / *t*Bu strategy. Amino acid side chains were protected as follows: Arg(Pbf), Asn(Trt), Asp(O*t*Bu), Cys(Trt), Gln(Trt), Glu(O*t*Bu), His(Trt), Lys(Boc), Ser(O*t*Bu), Thr(*t*Bu), Trp(Boc), Tyr(*t*Bu).

**Manual SPPS**

Peptides mini-Lys (**P19**) and mini-l-AsuHd (**P20**) were synthesized manually in 25 μmol scale on TentaGel HL RAM resin (capacity: 0.37 mmol / g).

Coupling reactions of standard amino acid building blocks (4 eq) were performed with HBTU (2-(1*H*-benzotriazol-1-yl)-1,1,3,3-tetramethyluronium hexafluorophosphate) (3.6 eq) as activator and NMM (*N*-methylmorpholine) (400 mM in DMF) as base for 1 h. Fmoc-l-AsuHd(OTrt)-OH (2 eq) was coupled using PyOxim ((ethylcyano-(hydroxyimino)acetato-*O*^2^)tri-1-pyrrolidinylphosphonium hexafluorophosphate) (2 eq) as activator. The Fmoc group was deprotected with piperidine (20% in DMF, 3×10 min). The resin was washed with DMF (3×), DCM (3×), DMF (3×) between each step.

Peptides were cleaved off the resin with a solution containing TFA, phenol, TIPS (triisopropylsilane) and water (10 mL, 85:5:5:5) under agitation for 3 h. After concentration under reduced pressure, cleaved peptides were precipitated in cold Et_2_O (40 mL), centrifuged (4000 *g*, 10 min, −4 °C), dissolved in water / MeCN, and lyophilized. Peptides were purified by preparative HPLC and analyzed by LC-MS.

**Automated SPPS**

Peptides **P1**–**P18** were synthesized on a MultiSynTech Syro I synthesizer (Witten, Germany) in 25 μmol scale on TentaGel HL RAM resin (capacity: 0.37 mmol / g).

Coupling reactions of the respective amino acids (3 eq) were performed by activation with DIC (*N*,*N*'-diisopropylcarbodiimide) (3 eq) and oxyma (ethyl 2-cyano-2-(hydroxyimino)acetate) (3 eq) in DMF for 40 min. Couplings were repeated once with HATU (*N*,*N*,*N*',*N*'-tetramethyl-*O*-(7-azabenzotriazol-1-yl)uronium hexafluorphosphate) (3 eq) as activator and NMM (6 eq) as base in DMF for 30 min. Fmoc-L-AsuHd(OTrt)-OH (2 eq) and Fmoc-Lys8(Boc)-OH (2 eq) were coupled manually with PyOxim (2 eq) and NMM (400 mM) in DMF for 1 h. Fmoc deprotection was performed by treating the resin twice with piperidine (40% in DMF) for a total of 15 min. Between each step the resin was rinsed with DMF. N-terminal acetylation was performed with Ac_2_O (5%) and 2,6-lutidine (6%) in DMF for 15 min.

Peptides were cleaved, purified and analyzed by LC-MS as described for manual synthesis.

**Automated 96-well SPPS**

Peptides for the 96-well assay were synthesized on an Intavis ResPep SL synthesizer (Cologne, Germany) in 96-well filter plates in 2 μmol scale on TentaGel S RAM resin (capacity: 0.23 mmol / g). Stock solutions of Fmoc-AsuHd(OTrt)-OH (**5**) and Fmoc-ApmHd(OTrt)-OH (**10**) were prepared in NMP and adjusted to pH 7 with formic acid prior to use.

Coupling reactions of the respective amino acids (5.25 eq) were performed with HATU (5 eq) as activator and NMM (10 eq) as base in DMF twice for 20 min. Fmoc-AsuHd(OTrt)-OH (**5**) (2.2 eq), Fmoc-ApmHd(OTrt)-OH (**10**) (2.2 eq) and Trt-Mpa-Lys(Dns)-OH (**16**) (2.2 eq) were coupled with HATU (2 eq) and NMM (10 eq) in DMF twice for 20 min. After each cycle unreacted amino groups were capped by treatment with a solution of Ac_2_O (5%) and 2,6-lutidine (6%) in DMF for 5 min. Fmoc deprotection was performed by treating the resin twice with piperidine (20% in DMF) for a total of 15 min. Between each step the resin was rinsed with DMF.

Peptides were cleaved off the resin with a solution containing TFA, water, phenol, thioanisole, DTT and TIPS (81.5:5:5:5:2.5:1, 600 μL / well) for 3 h in total. The solution was added in portions (1×200 μL and 3×100 μL) to each well and the resin was incubated for 30 min after each addition, except the last, after which it was incubated for 1.5 h. The resin was further rinsed with cleavage solution (100 μL / well) and the solution centrifuged into deep-well collection plates. Cleavage solutions were concentrated under ambient conditions in a fume hood overnight to a volume of approximately 200 μL / well. Cleaved peptides were precipitated in cold Et_2_O (700 μL / well), centrifuged (2500 *g*, 20 min, −4 °C), and washed with additional Et_2_O (2×500 μL / well). Peptides were dissolved in water / MeCN (1:1, 500 μL / well) and lyophilized. Crude peptides were analyzed by LC-MS.

**Peptide immobilization**

SulfoLink resin (300 μL) (Thermo Fisher Scientific, Rockford, USA), provided as 50% suspension, was drained and washed with coupling buffer (50 mM Tris·HCl, 5 mM EDTA-Na, pH 8.5) (5×800 μL) for each peptide. Peptide solution (1 mM in coupling buffer, 300 µL) was added and the resin was mixed for 15 min and then incubated for another 45 min at room temperature without agitation. After washing, the resin was blocked with blocking buffer (50 mM β-mercaptoethanol in coupling buffer, 500 µL). Subsequently, the resin was washed with NaCl solution (1 M, 6×1 mL), water (2×1mL) and MeCN / water (1:1, 4×1 mL). After draining, MeCN / water (1:1, 450 μL) was added to the beads resulting in a 2% suspension, which was stored in aliquots of 40 μL (10 μL resin) at −20 °C until usage.

**96-well peptide immobilization**

SulfoLink resin (200 μL / well) was transferred to a 96-deep-well filter plate, drained using a vacuum manifold, and washed with 96-well coupling buffer (coupling buffer containing 50% MeCN, 5×800 μL). Crude peptides were solved in 96-well coupling buffer at approximately 10 mM by assuming quantitative yield. Concentrations were adjusted to the same value according to fluorescence readout (λ_ex._ = 333 nm, bandwidth = 9 nm; λ_em._ = 568 nm, bandwidth = 20 nm) performed on an Infinite 200 PRO microplate reader (Tecan, Männedorf, Switzerland). Peptide solutions were diluted 10-fold with 96-well coupling buffer, added to the filter plate (200 µL / well), and the resin was mixed for 15 min and then incubated for another 45 min at room temperature without agitation. After washing with 96-well coupling buffer, the resin was blocked with blocking buffer (50 mM β-mercaptoethanol in 96-well coupling buffer, 500 µL / well). Subsequently, the resin was washed with NaCl solution (1 M, 6×1 mL / well), water (2×1mL / well) and MeCN / water (1:1, 4×1 mL / well). After draining, MeCN / water (1:1, 300 µL / well) was added to the beads resulting in a 25% suspension, from which aliquots of 40 μL (10 μL resin) were transferred to 96-well filter plates (MultiScreen_HTS_-HV, hydrophilic PVDF (0.45 μM), Merck Millipore, Darmstadt, Germany) and stored at −20 °C until usage.

**Cell culture**

HeLa S3 cells (DSMZ-No. ACC 161) or HEK293T (DSMZ-No. ACC 635) cells were cultured in Dulbecco’s Modified Eagle’s Medium (DMEM) (high glucose, Sigma-Aldrich) supplemented with 10% fetal calf serum (FCS) (Sigma-Aldrich) and 1% penicillin-streptomycin (Sigma-Aldrich) at 37 °C in a humidified atmosphere with 5% CO_2_. Subcultivation was performed every 2-4 days by treating the cells with 0.05% trypsin containing 0.2 g / L EDTA (Sigma-Aldrich). Afterwards cells were reseeded in fresh medium.

**siRNA transfection**

HeLa S3 cells were seeded in 6-well plates (2×10^5^ cells per well) 48 h prior to transfection with small-interfering RNA (siRNA). 50 pmol ON-TARGET*plus* SMARTpool HDAC6 or HDAC8 siRNA (KD) or ON-TARGET*plus* Non-targeting siRNA (NT) was delivered per well using the transfection reagent Dharmafect I (Thermo Fisher Scientific). After transfection, cells were cultivated for additional 48 h followed by RIPA buffer lysis.

**Preparation of cell extracts for acetylome analysis (RIPA buffer lysis)**

Prior to lysis, the medium was removed from the wells of a 6-well plate and each well was washed twice with 2 mL ice-cold PBS each. All following steps were performed at 4 °C or on ice. Per well, 150 μL RIPA buffer (50 mM Tris∙HCl, pH 8.0, 150 mM NaCl, 0.4% sodium deoxycholate, 0.1% SDS, 1% NP-40) were added and incubated for 15 min while shaking. Afterwards cells were collected using a cell scraper, the suspensions of three wells were combined and cleared by centrifugation at 16060 *g* for 15 min and the supernatant was precipitated in 1.5 mL precooled acetone. After 1 h incubation the mixture was centrifuged at 16060 *g* for 10 min and the supernatant discarded. The protein pellets were overlaid with 200 μL fresh acetone and stored at −80 °C until digestion in solution.

**Preparation of cell extracts for pulldown assays**

Hela S3 cells were harvested from culture dishes in ice-cold phosphate buffered saline (PBS, Sigma-Aldrich) with a cell scraper. Cytosolic (S100) and nuclear extracts (NE) were prepared according to the protocol of Dignam *et al.*^[71 72]^ All buffers were supplemented with 1 mM Na_3_VO_4_, 2 mM NaF and 0.5 mM 4-(2-aminoethyl)-benzensulfonyl fluoride (AEBSF) (instead of phenylmethylsulfonyl fluoride (PMSF)). S100 und NE fractions were combined to whole cell extract (WCE) and stored at -80 °C until usage. Total protein concentration was determined by the BCA assay performed with the Pierce BCA Protein Assay Kit (Thermo Fisher Scientific, Rockford, USA) according to the manufacturer’s manual.

For the analysis of p50 acetylation and the interaction HDAC6 between NF-κB1 and NF-κB2, HEK293T cells were transfected with the corresponding plasmids and harvested 24 h after transfection. Cells were washed in PBS and subsequently lysed using a lysis buffer containing 50 mM Tris·HCl pH 7.4, 150 mM NaCl, 1% NP-40, 50 mM NaF, 10 mM Na_4_P_2_O_7_,10 mM Na_4_V_2_O_7_ and Complete protease inhibitor cocktail (Roche).

**Pulldown assay**

One aliquot of resin-bound peptide probes **P11**–**P20** was transferred to a micro centrifuge filter unit (Ultrafree-MC-HV, Merck Milipore) and washed with pulldown buffer (20 mM HEPES, 100 mM KCl, 20% glycerol, pH 7.9) (3×200 μL) by shaking at 550 rpm for 2 min at room temperature. The resin was drained by centrifugation at 5000 rpm for 2 min after each step.

HeLa whole cell extract was diluted to 0.5 mg / mL of total protein concentration with pulldown buffer and the resin was incubated with 200 μL of this solution by shaking at 550 rpm for 1 h at room temperature. After draining, the resin was rinsed with washing buffer (20 mM HEPES, 300 mM KCl, 20% glycerol, pH 7.9) (6×500 μL) by shaking at 550 rpm for 2 min at room temperature and then dried by centrifugation.

Peptide-bound proteins were eluted by incubation with 20 μL of 3× SDS sample buffer (5× SDS sample buffer: 250mM Tris·HCl, 10% SDS, 30% glycerol, 0.5 M DTT, 0.02% bromophenol blue) for 10 min at 95 °C and 550 rpm, followed by centrifugation at 12000 rpm for 5 min. Input samples were added as reference by mixing 16 μL of HeLa whole cell extract solution (1 mg / mL) with 4 μL of 5× SDS sample buffer and heating for 10 min at 95 °C. Samples were separated by SDS-PAGE.

Pulldown assays of interactome analysis were performed as described above with three different batches of WCE (0.5 mg / mL total protein concentration), using SDS sample buffer without bromophenol blue. Input samples were generated by mixing 20 μL of HeLa whole cell extract solution (0.5 mg / mL) with 30 μL 5× SDS sample buffer without bromophenol blue. Eluted proteins were stored at 4 °C until subjected to filter aided sample preparation (FASP).

Pulldown assays with recombinant HDACs (HDAC6: full length with C-terminal FLAG-tag, catalog # 50056, HDAC1: full length with C-terminal His-tag followed by FLAG-tag, catalog # 50051, HDAC8: full length with C-terminal His-tag, catalog # 50008, all BPS Bioscience, San Diego, USA) were performed as described above by incubation of PPIA probes (**P15**, **P20**) with increasing concentrations of recombinant enzymes (0.5–10 nM in 200 µL pulldown buffer containing 20 mg / mL BSA). Washing was performed with washing buffer (6×200 μL) by shaking at 550 rpm for 5 min and draining by centrifugation. Input samples contained 20 ng recombinant HDAC.

For the analysis of the HDAC6 interaction with NF-κB1 and NF-κB2, HEK293T lysates were incubated with Strep-Tactin sepharose resin (IBA Lifesciences, Göttingen, Germany) for 1 h at 4 °C. Beads were washed four times with 1 ml of lysis buffer and incubated for 10 min at 95 °C with SDS sample buffer.

**96-well pulldown assay**

One 96-well filter plate with resin-bound peptide probes was washed with pulldown buffer (3×200 μL / well) by shaking at 550 rpm for 2 min at room temperature. The resin was drained after each step using a vacuum manifold.

HeLa whole cell extract was diluted to 1 mg / mL (0.5 mg / mL for p53 probes) of total protein concentration with pulldown buffer and the resin was incubated with this solution (200 µL / well) by shaking at 550 rpm for 1 h at room temperature. After draining, the resin was rinsed with washing buffer (6×200 μL / well) by shaking at 550 rpm for 2 min at room temperature and then drained.

Peptide-bound proteins were eluted by incubation with 3× SDS sample buffer (30 µL / well) for 20 min at 70 °C and 550 rpm, followed by centrifugation at 3000 rpm for 10 min into 96-well collection plates. Input samples were added as reference by mixing 16 μL of HeLa whole cell extract solution (1 mg / mL) with 4 μL of 5× SDS sample buffer and heating for 20 min at 70 °C. Samples were separated by SDS-PAGE.

**Western blotting**

Eluted proteins were separated by SDS-PAGE and transferred onto PVDF membranes (Amersham Hybond Low Fluorescence 0.2 μm, GE Healthcare, Freiburg, Germany) by western blotting in a wet tank system.

The membrane was blocked by incubation with blocking buffer (5% low fat powdered milk in TBS (20 mM Tris, 150 mM NaCl, pH 7.3)) for 30 min at room temperature.

After washing with TBST (20 mM Tris, 150 mM NaCl, 0.1% Tween 20, pH 7.3) membranes were incubated with the respective primary antibodies diluted into TBST buffer containing 2.5% low fat powdered milk at 4 °C overnight. The following antibodies and dilutions were used: HDAC1 mouse monoclonal IgG (1:500, 10E2, sc-81598, Santa Cruz Biotechnology, Dallas, USA), HDAC6 rabbit polyclonal IgG (1:500, H-300, sc-11420, Santa Cruz Biotechnology), HDAC6 mouse monoclonal IgG (1:500, D-11, sc-28386, Santa Cruz Biotechnology), HDAC8 (1:200, MA5-32422, Thermo Fisher Scientific), Acetyllysine (1:1000, #9441, Cell Signaling), p105/p50 (1:1000, #13586, Cell Signaling), p100/p52 (1:1000, #4882, Cell Signaling), FLAG OctA-Probe rabbit polyclonal IgG (1:1000, D-8, sc-807, Santa Cruz Biotechnology), Penta-His mouse monoclonal IgG (1:1000, 34660, Quiagen), FLAG M2 mouse monoclonal IgG (1:1000, M2, Merck Millipore), Myc-Tag (1:1000, #2272, Cell Signaling), StrepMab (1:1000, 2-1507-001, IBA)

The membrane was washed afterwards with TBST and incubated with the respective secondary antibody solutions for 1 h at room temperature: Recombinant anti-mouse IgG binding protein (1:5000, sc-516102, Santa Cruz Biotechnology), mouse anti-rabbit IgG-HRP (1:5000, sc-2357, Santa Cruz Biotechnology), donkey anti-rabbit IgG-IRDye 800 CW (1:5000, 926-32213, LI-COR), donkey anti-mouse IgG-IRDye 800 CW (1:5000, 926-32212, LI-COR).

After washing with TBST the membranes were developed with Pierce ECL Western Blotting Substrate Kit (Thermo Fisher Scientific) for HRP conjugates. Chemiluminescence or fluorescence was detected using a ChemiDoc MP imaging system (Bio-Rad Laboratories, Munich, Germany). Full-size blots are shown in supporting Figures S30 to S34.

**Reporter assay of NF-κB target gene activation**

The reporter assay was performed as reported before.^[73]^ In brief, HEK293T cells were transiently transfected with the expression and reporter plasmids by the calcium phosphate precipitation method. The dual luciferase reporter assay was performed as described in Dual-Glo Luciferase Assay System Kit provided by Promega. Experiments were performed in triplicate using Student’s t-test for statistical analysis. The NF-κB and TK-Renilla reporter constructs were kindly provided by Margot Thome (University of Lausanne, Switzerland).

**MALDI-MS-based deacetylation assay**

Deacetylation reactions were performed with recombinant HDAC6 (100 nM, full length with C-terminal FLAG-tag, catalog # 50056, BPS Bioscience, San Diego, USA) and peptide substrates **P1**–**P5** (100 μM) in HDAC buffer (100 mM HEPES, 8 mM KCl, 100 mM NaCl, pH 8.0) at 37 °C. At selected time points (0 to 25 min) samples (2 μL) were taken and mixed with stopping solution (8 μL, 6.25 μM trichostatin A, 0.1% TFA in water). To this mixture a solution of the respective isotopic standard **P6**–**P10** (10 μL, 4 μM in HDAC buffer) was added and the sample was further diluted with 0.1% TFA in water (80 μL). 1 μL of this solution was spotted onto a polished steel target, mixed with 1 μL of matrix solution (CHCA for **P2**–**P5** and **P7**–**P10**, DHB for **P1** / **P6**), and analyzed by MALDI-MS. The amount of formed product was determined from the signal intensity of the monoisotopic [M+H]^+^ (**P2**–**P5**) or [M+Na]^+^ (**P1**) peak of the product in relation to the respective peak of the isotopically labeled references **P5**–**P10**. Initial velocities of the deacetylation reaction were determined from three independent experiments.

**Filter aided sample preparation (FASP)**

Protein samples from pull-down assay were processed for interactome profiling using the FASP method as described previously.^[12]^ In brief, 450 μL urea buffer (8 M urea, 0.1 M Tris∙HCl, pH 8.5) was added to eluted proteins or the input and the samples were incubated at 95 °C for 5 min under agitation, and the mixtures were transferred to centrifuge filter units (Microcon YM-10, Merck Millipore). After centrifugation (13900 *g*, 20 min) the filtrate was discarded. The samples were washed with urea buffer (3 × 450 μL) and drained by centrifugation (13900 *g*, 20 min). Chloroacetamide (55 mM in urea buffer, 100 μL) was added, the samples were incubated in the dark for 20 min without shaking, and centrifuged (13900 *g*, 15 min). The samples were washed with urea buffer (3 × 100 μL) and drained by centrifugation (13900 *g*, 10 min). The collection tubes were replaced and the samples were subjected to enzymatic cleavage. To this end, 1 μg Lysyl endopeptidase (Lys-C, Wako Chemicals, Neuss, Germany) (0.5 μg / μL in 50 mM ammonium bicarbonate (ABC) buffer) in 40 μL urea buffer was added and the samples were incubated at room temperature for 1 h while shaking. Additional Lys-C (1 μg) was added and the mixture was incubated over night at room temperature under agitation. The solution was diluted with ABC buffer (300 µL) and 0.2 μg trypsin (MS approved, SERVA, Heidelberg, Germany) (1 μg / μL in 1 mM HCl) were added. The samples were incubated at 37 °C for 3.5 h under agitation, followed by an additional trypsination step. After centrifugation (13900 *g*, 10 min), ABC buffer (50 μL) was added and the centrifugation step was repeated. The eluates were acidified with TFA to a final concentration of 1% and desalted using C18-StageTips (pulldown samples) or SDB-StageTips (3 fractions, input samples).^[74]^

**In-solution digestion of proteins for acetylome profiling**

Acetone was removed from the precipitated protein pellets, and they were air dried for 10 min. Proteins were then dissolved in 350 μL urea buffer (6 M urea, 2 M thiourea, 10 mM HEPES, pH 7.9) for 20 min at 40 °C while sonicating. The following steps were all performed at room temperature unless stated otherwise. Protein disulfide bonds were reduced by addition of DTT to a final concentration of 10 mM and incubation for 20 min while shaking. Alkylation was carried out with chloroacetamide in a final concentration of 5.5 mM in the dark for 20 min without shaking. Afterwards, proteins were digested by incubation with 25 μg Lys C (1:100 in relation to protein) for 1 h while shaking. Another 25 μg Lys C were added and the mixture was incubated over night while shaking. The solution was then diluted with 2.35 mL ABC buffer and 25 μg trypsin (1:100 in relation to protein) were added. The mixture was incubated for 3 h at 37 °C while shaking. Afterwards another 25 μg trypsin were added, followed by incubation over night at 37 °C while shaking. The resulting peptide solution was acidified with TFA to a final concentration of 1% and subjected to on-column dimethyl labeling.

**On-column dimethyl labeling**^[75]^

Prior to dimethyl labeling two different labeling solutions were prepared: Light (0.5 mL 4% formaldehyde CH_2_O in water, 0.5 mL 600 mM NaBH_3_CN in water, 4 mL 50 mM sodium phosphate buffer, pH 7.5 and 15 mL water) and intermediate (0.5 mL 4% deuterated formaldehyde (CD_2_O) in water, 0.5 mL 600 mM NaBH_3_CN in water, 4 mL 50 mM sodium phosphate buffer, pH 7.5 and 15 mL water).

The labeling was performed on Sep-Pak C18 reverse phase cartridges (Waters Corporation) separately for each replicate of the KD HDAC6 and NT peptide solutions. First, the column material was equilibrated and washed with 3 mL methanol, 3 mL buffer B (80% MeCN, 0.5% formic acid in water, all LCMS grade) and 3 mL buffer A (0.5% formic acid in water). The acidified peptide solutions were then slowly loaded onto the columns by gravity flow. Afterwards two washing steps were performed with 1 mL and 3 mL buffer A. Per sample, 5 mL of the respective labeling solution were added to the columns: KD HDAC6 was labeled “light” and NT was labeled “intermediate”. Each experiment was performed in duplicate and a label swap was included.

After the labeling solutions were run through the columns two washing steps were performed with 1 mL and 3 mL buffer A. The labeled peptides were then eluted from the columns in two steps by treatment with buffer B (2×0.7 mL). The concentrations of the eluates were measured via NanoDrop (ND-1000, Peqlab) and equal amounts of peptides of different samples were combined: (KD replicate 1 (intermediate) and NT replicate 1 (light), KD replicate 2 (intermediate) and NT replicate 2 (light), KD replicate 3 (light) and NT replicate 3 (intermediate), KD replicate 4 (light) and NT replicate 4 (intermediate)). The mixtures were lyophilized and afterwards subjected to acetyllysine antibody agarose enrichment.

**Enrichment on acetyllysine antibody agarose**

The lyophilized peptides were dissolved in 1 mL TBS and the pH was adjusted to 7 with 1 M Tris∙HCl, pH 7.6. The concentration was determined via NanoDrop and 15 μg of each sample were mixed with 100 μL 1% TFA in water generating an input sample for measuring the total proteome. The input solutions were loaded onto and eluted from SDB-StageTips (3 fractions, input samples).^[74]^ All steps involving the acetyllysine antibody were carried out on ice or at 4 °C. 50 μL of the antibody bead slurry (ImmuneChem Pharmaceuticals Inc.) were washed with 1 mL TBS for 5 min on an overhead shaker. The suspension was centrifuged at 1000 *g* for 2 min, the supernatant discarded, and this step was repeated twice. Afterwards the dissolved peptide solutions were incubated overnight on an overhead shaker. The suspension was centrifuged at 1000 *g* for 2 min and the beads were successively washed with TBS (5×1 mL) and water (2×1 mL). Elution of the bound peptides was performed by adding 100 μL 1% TFA in water to the agarose beads, incubating for 5 min on an overhead shaker, centrifuging at 1000 *g* for 2 min and collecting the supernatant. This step was repeated twice, and the combined eluates were loaded onto SCX-StageTips. The elution was performed with increasing pH value yielding six fractions.^[74]^

**Nano-LC-MS/MS**

Peptides from pulldown assays and for HDAC6 and HDAC8 knockdown acetylome analysis were separated using an EASY-nLC1200 UHPLC system (Thermo Fisher Scientific) equipped with either a 16 cm column (pulldowns) or 20 cm column (acetylomes), both packed in-house with ReproSil-Pur C18-AQ 1.9 μm resin (Dr. Maisch). The column temperature was maintained at 50 °C, and the column was coupled to a Q Exactive HF mass spectrometer (Thermo Fisher Scientific) via a nano-electrospray source.

For pulldown experiments total peptides (0.5 μg) were loaded onto the column and separated over a segmented linear gradient from 3-80% buffer B (0.5% formic acid in MeCN) in 120 min. The mass spectrometer was operated in data-dependent mode, survey scans were obtained in a mass range of 300–1759 *m* / *z*, at a resolution of 120000 at 200 *m* / *z* and an AGC target value of 3 × 10^6^. The 12 most intense ions were selected with an isolation width of 1.2 *m* / *z*, fragmented in the HCD cell at a collision energy of 25 and the spectra recorded at a target value of 5 × 10^4^ and a resolution of 15000. Peptides with a charge of +1 or +6 and higher were excluded from fragmentation, the peptide match and exclude isotope features were enabled and selected precursors were dynamically excluded from repeated sampling for 30 s.

For acetylome experiments total peptides (0.5 μg; dissolved in 2% ACN, 0.1% TFA in water) were loaded on the column and separated over a segmented linear gradient from 5% to 98% buffer B. The mass spectrometer was operated in data-dependent mode, the peptide match was set as “preferred”, exclude isotope feature was enabled, and selected precursors were dynamically excluded from repeated sampling.

DIA analysis of HDAC8 input samples was performed by using an easyLC 1200 UHPLC-System coupled to an Orbitrap Exploris 480 Mass Spectrometer (Thermo Fisher). Separation of peptides was performed on 20 cm frit-less silica emitters (CoAnn Technologies, 0.75 µm inner diameter), packed in-house with reversed-phase ReproSil-Pur C18 AQ 1.9 µm resin (Dr. Maisch). The column was constantly kept at 50 °C. Peptides were eluted in 115 min applying a segmented linear gradient of 0 % to 98% solvent B (solvent A: 0% ACN, 0.1% FA; solvent B: 80% ACN, 0.1% FA) at a flow-rate of 300 nL/min.

Mass spectra were acquired in data-independent acquisition (DIA) mode using the respective template of the method editor: MS1 scans were acquired at an Orbitrap Resolution of 60 000 with a Scan Range (m/z) of 380-985, a maximum injection time of 100 ms and a normalized AGC Target of 100%. For MS2 scans the Isolation Window was set to 10 m/z with no offset for precursors from 380 – 980 m/z. The scan range was 145 – 1450 m/z. A normalized collision energy of 28 was used. MS2 scans were taken at an Orbitrap Resolution of 15 000. Maximum injection time was 40 ms and the normalized AGC Target 200%.

**Data processing and quantification**

Raw data were processed using the MaxQuant software package (Version 1.6.3.4 (interactomes, 2.1.3.0 (acetylomes), https://www.maxquant.org/)^[76]^ and searched against the human reference proteome (UP000005640_9606, https://www.uniprot.org/) and an internal database containing standard contaminants. The search was performed with full trypsin specificity and a maximum of two (Kac-enriched samples: four) missed cleavages and a protein false discovery rate of 1% was set. Minimal peptide length was set to seven amino acids. Carbamidomethylation of cysteine residues was set as fixed, oxidation of methionine and N-terminal acetylation (additionally lysine acetylation for the respective samples) were set as variable modifications. For acetylome samples DimethLys0 and DimethNter0 as well as DimethLys4 and DimethNter4 were set as labels. Match between runs and requantify options were enabled. All other search parameters were left at default. For interactomes the MaxLFQ algorithm integral to MaxQuant was used for label-free quantification with match between runs enabled and the LFQ minimum ratio count was set to 1.^[77]^ Input and pull-down samples were searched in two different parameter groups.

Downstream data analysis was carried out in the Perseus software package (Version 1.6.1.1, https://www.maxquant.org/)^[78]^ and R. All hits for contaminants and reversed sequences were removed and the LFQ values or dimethyl ratios log_2_ transformed. For interactomes missing LFQ values were imputed for each sequence context of peptides **P11**–**P20** separately from normal distributions for proteins which were detected at least once on Lys-containing and at least once on AsuHd-containing probes and in at least three replicates in total on either Lys-containing or AsuHd-containing probes. Imputation was carried out for each column separately with a width of 0.5 and a down shift of 1.8. For acetylomes ratios of reverse label experiments were inverted and the mean ratio for forward and reverse replicates calculated. Sites were expanded to allow separate analysis of site ratios for multiply acetylated peptides occurring in different acetylation states. The four replicates were averaged and proteins or sites displaying less than two out of four ratios were removed. For interactomes statistical analysis was performed with the limma package^[79]^ for R (Version 3.5.2, https://www.r-project.org/) using the log_2_ ratios from three independent biological replicates. Volcano plots were generated with Microsoft Excel, plotting p-values from limma analysis against the log_2_ ratio. The input samples of the HDAC8 acetylome were in addition subjected to DIA analysis. Data was analyzed with DIA-NN confirming a log_2_ reduction of HDAC8 abundance of 4.54-fold in the knockdown lysates (Supporting Table S13): Data was processed using DIA-NN 2.0.2.^[80]^ A library was predicted from a fasta file of the latest version of the human reference proteome from UniProt (2025-03-25, 20414 entries)^[81]^ supplemented with 48 common contaminants.^[82]^ This library was used in a search with the following additional options to enable plexDIA: ^[83, 84]^

--fixed-mod Dimethyl, 28.0313, nK

--lib-fixed-mod Dimethyl

--channels Dimethyl,0,nK,0:0; Dimethyl,4,nK,4.0251:4.0251

--original-mods

The DIA-NN report was imported and further processed with R 4.0.0 using the arrow and the diann package (version 1.0.1),^[85]^ quantifying protein groups by using the channel specific intensities. Differential expression analysis was performed using limma.^[79]^

**Data availability**

The proteomics data are deposited at the jPOST repository (https://repository.jpostdb.org) with the data set identifiers:

JPST002258 (DDA analysis of probe interactomes)

<https://repository.jpostdb.org/entry/JPST002258.0>

JPST003724 (DIA-based comparative expression analysis of wt and ko line)

<https://repository.jpostdb.org/entry/JPST003724.0>

**Supporting references**

[12] A. Dose, J. Sindlinger, J. Bierlmeier, A. Bakirbas, K. Schulze-Osthoff, F. Einsele-Scholz, M. Hartl, F. Essmann, I. Finkemeier, D. Schwarzer, *Angew. Chem. Int. Ed.* **2016**, *55*, 1192–1195.

[39] C. Hubbert, A. Guardiola, R. Shao, Y. Kawaguchi, A. Ito, A. Nixon, M. Yoshida, X.-F. Wang, T.P. Yao, *Nature* **2002**, *417*, 455–458.

[46] C. Schölz, B. T. Weinert, S. A. Wagner, P. Beli, Y. Miyake, J. Qi, L. J. Jensen, W. Streicher, A. R. McCarthy, N. J. Westwood, S. Lain, J. Cox, P. Matthias, M. Mann, J. E. Bradner, C. Choudhary, *Nat. Biotechnol.* **2015**, *33*, 415–423.

[63] The UniProt Consortium, *Nucleic Acids Res.* **2023**, *51*, D523–D531.

[64] M. New, H. Olzscha, G. Liu, O. Khan, L. Stimson, J. McGouran, D. Kerr, A. Coutts, B. Kessler, M. Middleton, N. B. La Thangue, *Cell Death Differ.* **2013**, *20*, 1306–1316.

[65] M. Zhang, S. Xiang, H.-Y. Joo, L.Wang, K. A. Williams, W. Liu, C. Hu, D. Tong, J. Haakenson, C. Wang, S. Zhang, R. E. Pavlovicz, A. Jones, K. H. Schmidt, J. Tang, H. Dong, B. Shan, B. Fang, R. Radhakrishnan, P. M. Glazer, P. Matthias, J. Koomen, E. Seto, G. Bepler, S. V. Nicosia, J. Chen, C. Li, L. Gu, G.-M. Li, W. Bai, H. Wang, X. Zhang, *Mol. Cell* **2015**, *55*, 31–46.

[66] C.-J. Yang, Y.-P. Liu, H.-Y. Dai, Y.-L. Shiue, C.-J. Tsai, M.-S. Huang, Y.-T. Yeh, *Oncotarget* **2015**, *6*, 30263–30276.

[67] X. Xu, P. Ding, L. Shi, G. Wu, X. Ma, *BMC Cancer* **2022**, *22*, 630.

[68] D. Kaluza, J. Kroll, S. Gesierich, T.-P. Yao, R. A. Boon, E. Hergenreider, M. Tjwa, L. Rössig, E. Seto, H. G. Augustin, A. M. Zeiher, S. Dimmeler, C. Urbich, EMBO J. **2011**, *30*, 4142–4156.

[69] M. Y. Hein, N. C. Hubner, I. Poser, J. Cox, N. Nagaraj, Y. Toyoda, I. A. Gak, I. Weisswange, J. Mansfeld, F. Buchholz, A. A. Hyman, M. Mann, *Cell* **2015**, *163*, 712–723.

[16] C. Moreno-Yruela, M. Bæk, A.-E. Vrsanova, C. Schulte, H. M. Maric, C. A. Olsen, *Nat. Commun.* **2021**, *12*, 62.

[70] T. Hulsen, J. de Vlieg, W. Alkema, *BMC Genom.* **2008**, *9*, 488.

[51] J. O. Jost, A. Hanswillemenke, D. Schwarzer, *Mol. BioSyst.* **2015**, *11*, 1820–1823.

[71] M. Strohalm, D. Kavan, P. Novák, M. Volný, V. Havlíček, *Anal. Chem.* **2010**, *82*, 4648–4651.

[72] J. D. Dignam, P. L. Martin, B. S. Shastry, R. G. Roeder, *Methods Enzymol.* **1983**, *101*, 582–598.

[73] S. Hailfinger, H. Nogai, C. Pelzer, M. Jaworski, K. Cabalzar, J.-E. Charton, Montserrat Guzzardi, C. Décaillet, M. Grau, B. Dörken, P. Lenz, G. Lenz, M. Thome, *Proc. Natl. Acad. Sci. U. S. A*. **2011**, *108*, 14596–14601.

[74] J. Rappsilber, M. Mann, Y. Ishihama, *Nat. Protoc.* **2007**, *2*, 1896–1906.

[75] P. J. Boersema, R. Raijmakers, S. Lemeer, S. Mohammed, A. J. R. Heck, *Nat. Protoc.* **2009**, *4*, 484–494.

[76] J. Cox, M. Mann, *Nat. Biotechnol.* **2008**, *26*, 1367–1372.

[77] J. Cox, M. Y. Hein, C. A. Luber, I. Paron, N. Nagaraj, M. Mann, *Mol. Cell. Proteomics* **2014**, *13*, 2513–2526.

[78] S. Tyanova, T. Temu, P. Sinitcyn, A. Carlson, M. Y. Hein, T. Geiger, M. Mann, J. Cox, *Nat. Methods* **2016**, *13*, 731–740.

[79] M. E. Ritchie, B. Phipson, D. Wu, Y. Hu, C. W. Law, W. Shi, G. K. Smyth, *Nucleic Acids Res.* **2015**, *43*, e47. 41-44.

[80] V. Demichev, C. B. Messner, S. I. Vernardis, K. S. Lilley, M. Ralser, Nat Methods 2020, 17, 41-44.

[81] UniProt, *Nucleic Acids Res*. **2025**, *53*, D609-D617.

[82] Mellacheruvu, Z. Wright, A. L. Couzens, J. P. Lambert, N. A. St-Denis, T. Li, Y. V. Miteva, S. Hauri, M. E. Sardiu, T. Y. Low, V. A. Halim, R. D. Bagshaw, N. C. Hubner, A. Al-Hakim, A. Bouchard, D. Faubert, D. Fermin, W. H. Dunham, M. Goudreault, Z. Y. Lin, B. G. Badillo, T. Pawson, D. Durocher, B. Coulombe, R. Aebersold, G. Superti-Furga, J. Colinge, A. J. Heck, H. Choi, M. Gstaiger, S. Mohammed, I. M. Cristea, K. L. Bennett, M. P. Washburn, B. Raught, R. M. Ewing, A. C. Gingras, A. I. Nesvizhskii, *Nat. Methods* **2013**, *10*, 730-736.

[83] M. Thielert, E. C. Itang, C. Ammar, F. A. Rosenberger, I. Bludau, L. Schweizer, T. M. Nordmann, P. Skowronek, M. Wahle, W. F. Zeng, X. X. Zhou, A. D. Brunner, S. Richter, M. P. Levesque, F. J. Theis, M. Steger, M. Mann, *Mol. Syst. Biol.* **2023**, *19*, e11503.

[84] J. Derks, A. Leduc, G. Wallmann, R. G. Huffman, M. Willetts, S. Khan, H. Specht, M. Ralser, V. Demichev, N. Slavov, *Nat. Biotechnol.* **2023**, *41*, 50-59.

[85] Software package deposited: https://github.com/vdemichev/diann-rpackage.

**NMR spectra and LC-MS data**


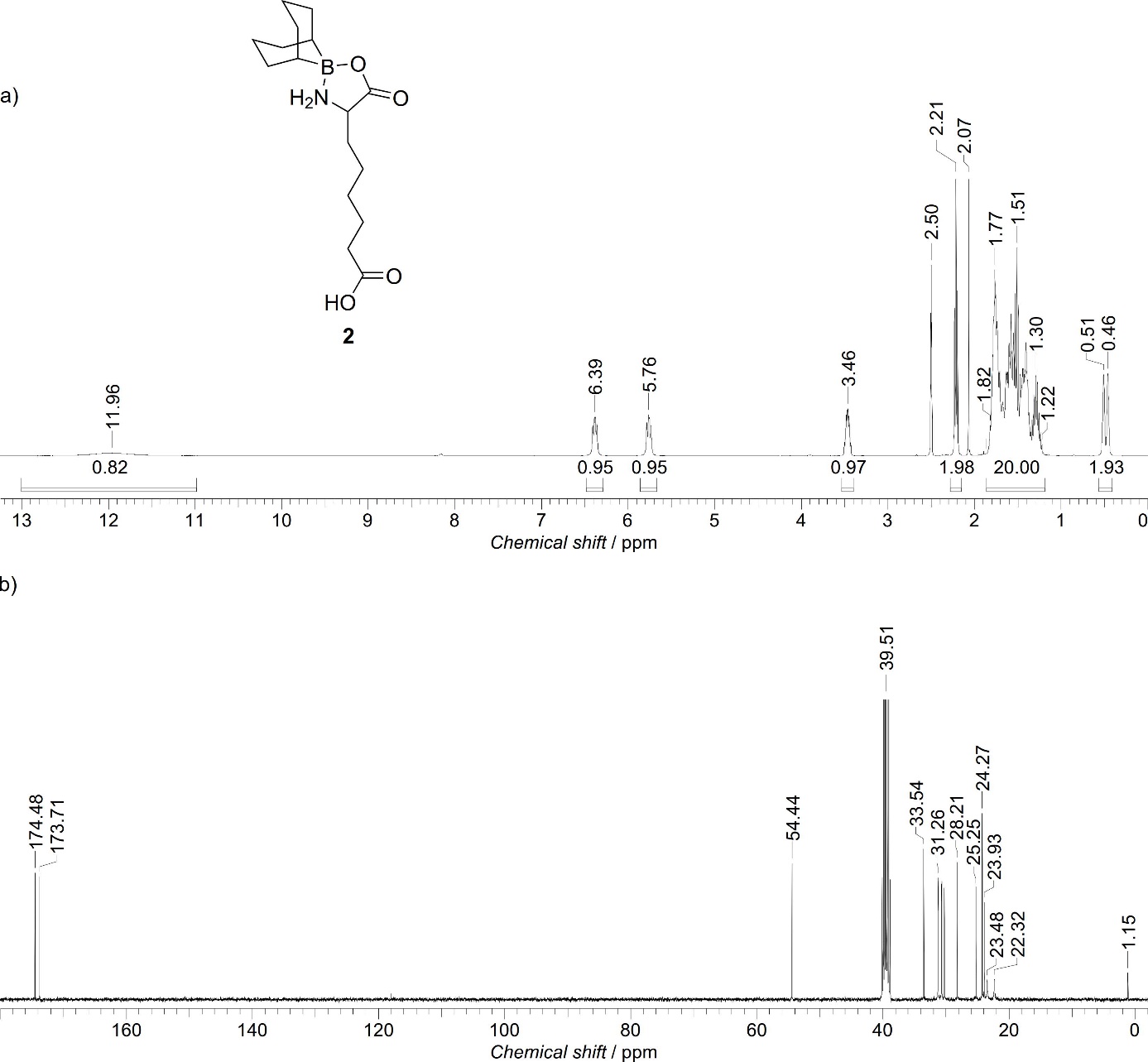


**Supporting Figure S11:** a) ^1^H-NMR spectrum (400 MHz, DMSO-d_6_) and b) ^13^C-NMR spectrum (101 MHz, DMSO-d_6_) of Asu-BBN (**2**).


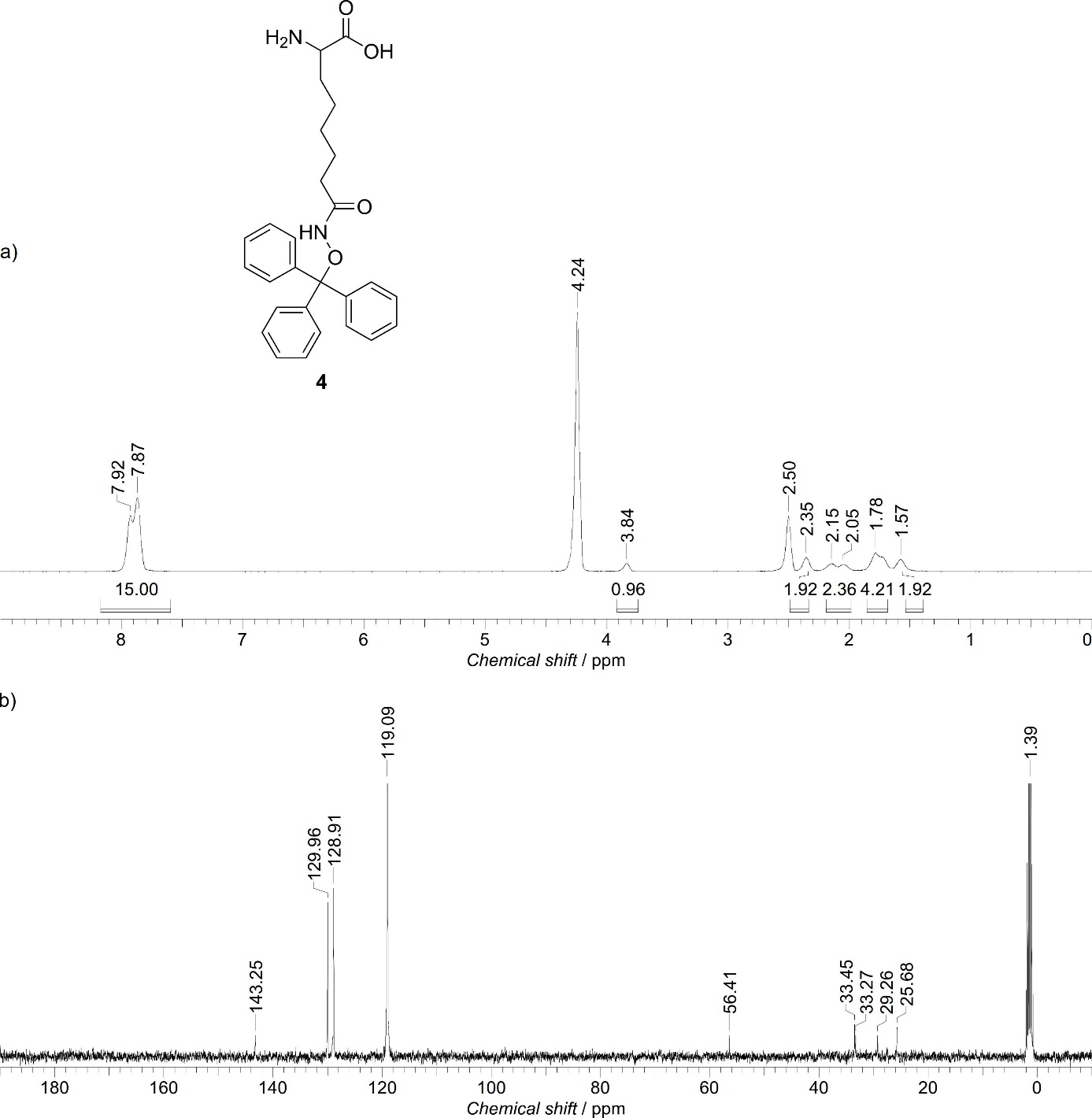


**Supporting Figure S12:** a) ^1^H-NMR spectrum (400 MHz, MeCN-d_3_ / D_2_O (3:1)) and b) ^13^C-NMR spectrum (101 MHz, MeCN-d_3_ / D_2_O (3:1)) of H-AsuHd(OTrt)-OH (**4**).

**
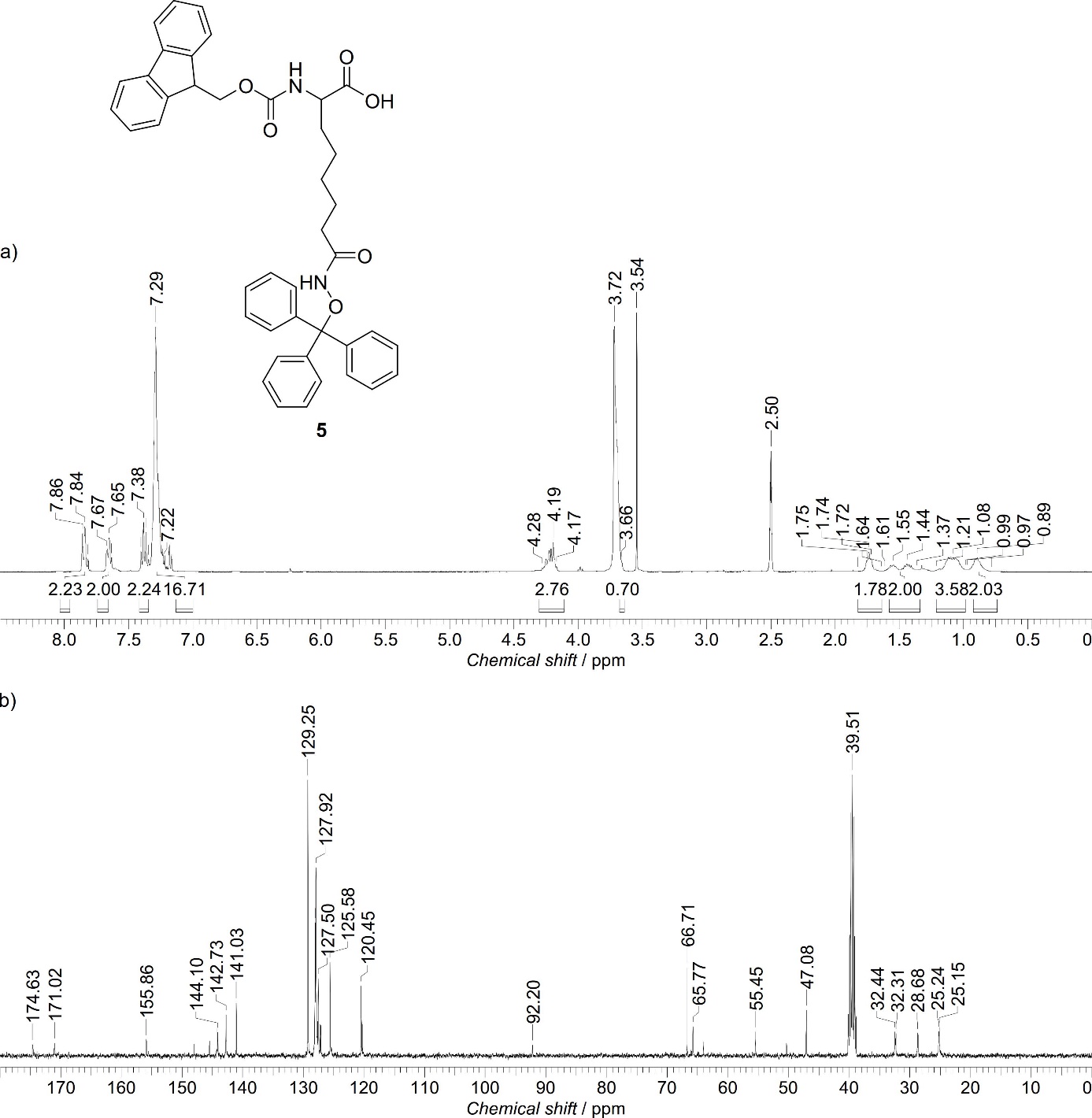
**

**Supporting Figure S13:** a) ^1^H-NMR spectrum (400 MHz, DMSO-d_6_ / 200 mM Na_2_HPO_3_ in D_2_O, pH 7 (9:1)) and b) ^13^C-NMR spectrum (101 MHz, DMSO-d_6_ / 200 mM Na_2_HPO_3_ in D_2_O, pH 7 (9:1)) of Fmoc-AsuHd(OTrt)-OH (**5**).

**
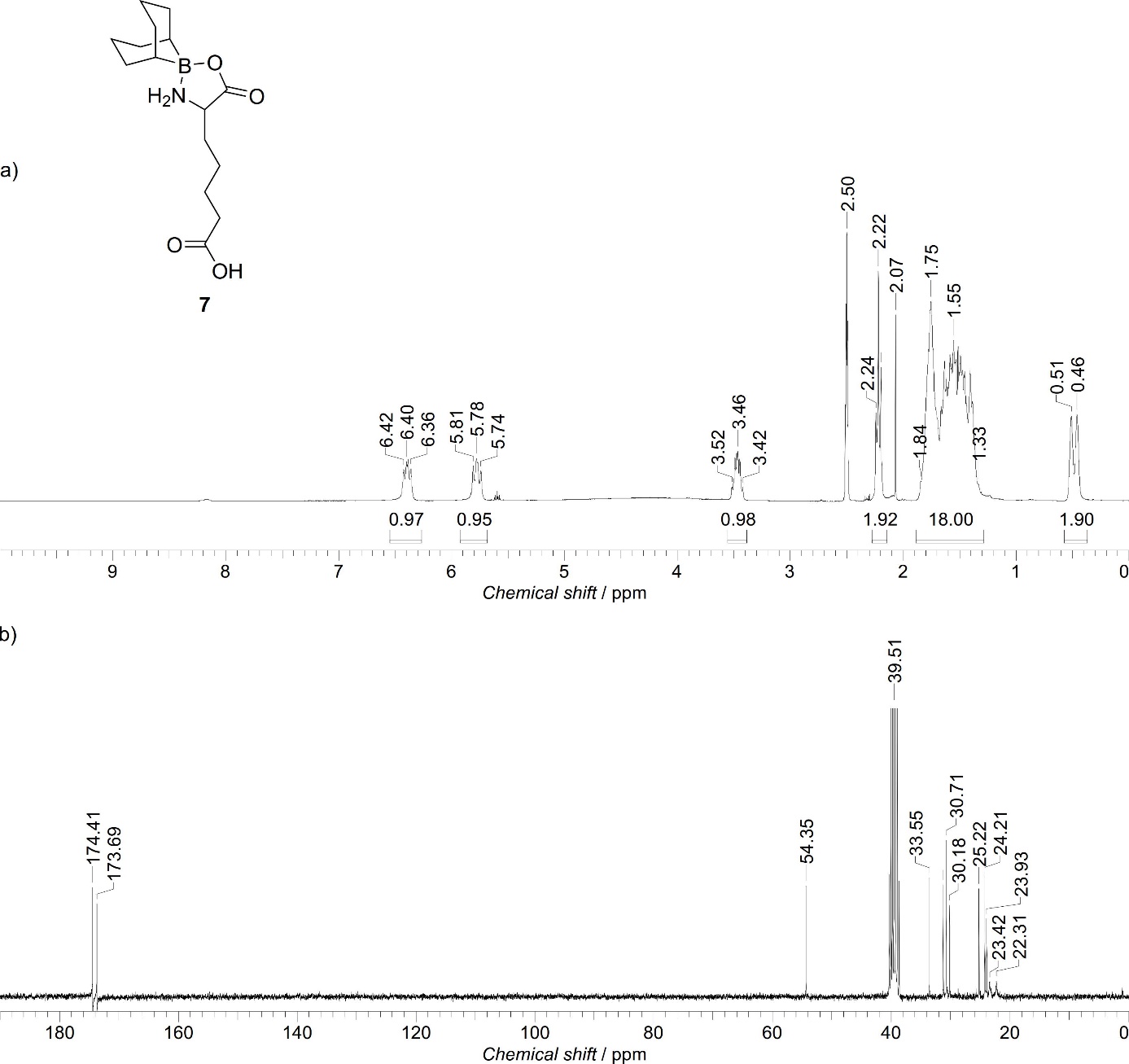
**

**Supporting Figure S14:** a) ^1^H-NMR spectrum (400 MHz, DMSO-d_6_) and b) ^13^C-NMR spectrum (101 MHz, DMSO-d_6_) of Apm-BBN (**7**).


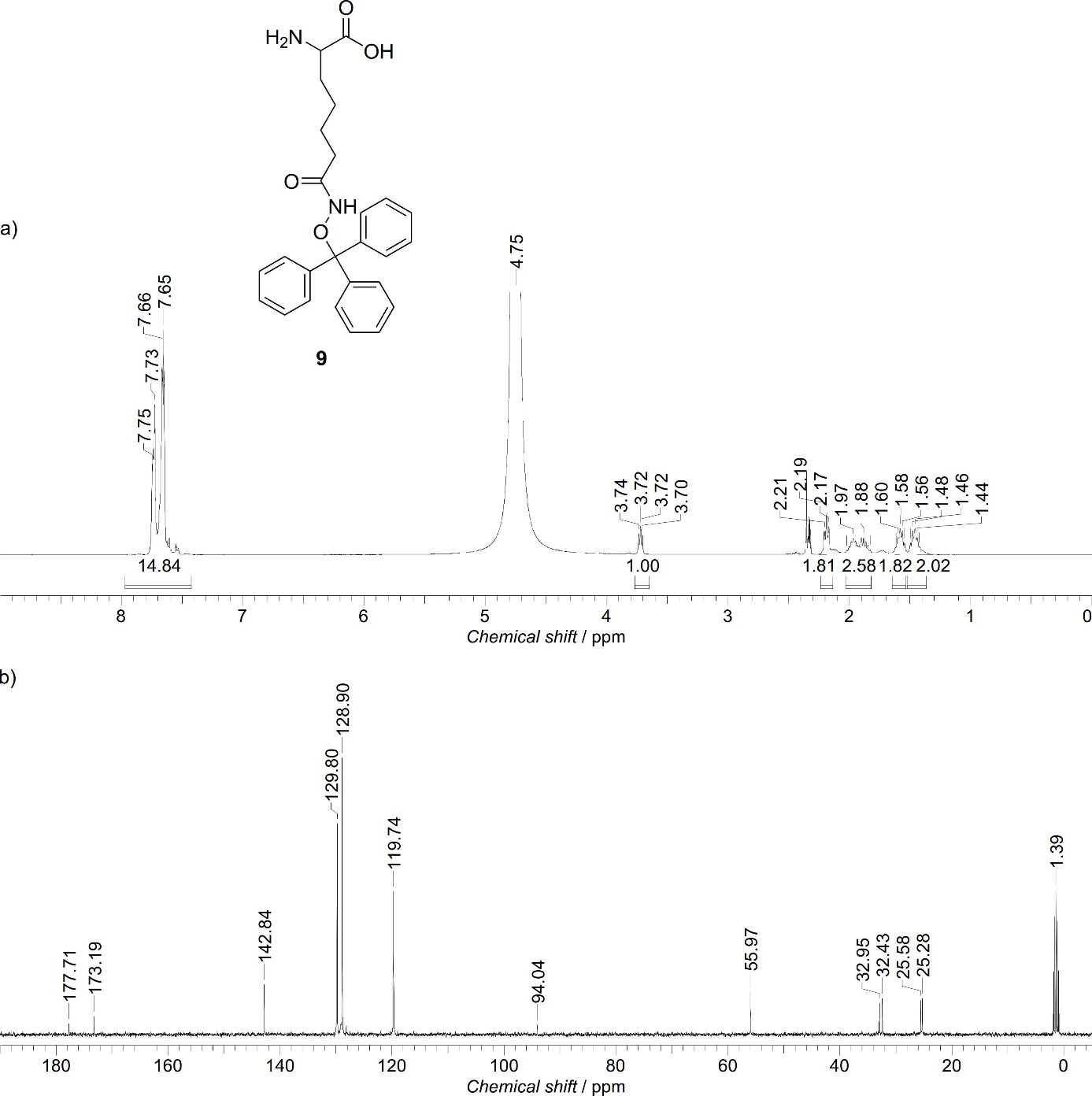


**Supporting Figure S15:** a) ^1^H-NMR spectrum (400 MHz, MeCN-d_3_ / D_2_O (1:1)) and b) ^13^C-NMR spectrum (101 MHz, MeCN-d_3_ / D_2_O (1:1)) of H-ApmHd(OTrt)-OH (**9**).


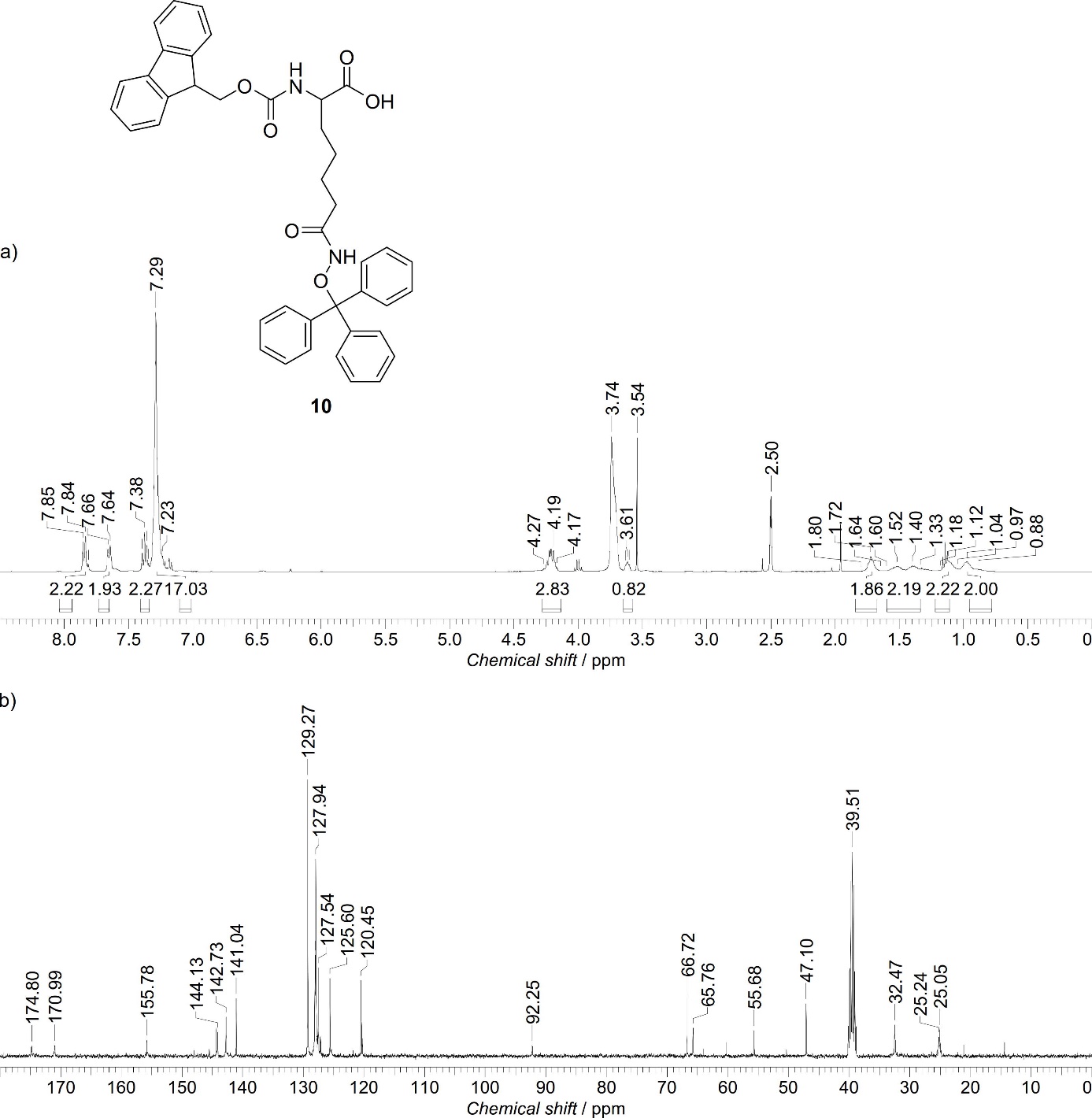


**Supporting Figure S16:** a) ^1^H-NMR spectrum (400 MHz, DMSO-d_6_ / 200 mM Na_2_HPO_3_ in D_2_O, pH 7 (9:1)) and b) ^13^C-NMR spectrum (101 MHz, DMSO-d_6_ / 200 mM Na_2_HPO_3_ in D_2_O, pH 7 (9:1)) of Fmoc-ApmHd(OTrt)-OH (**10**).


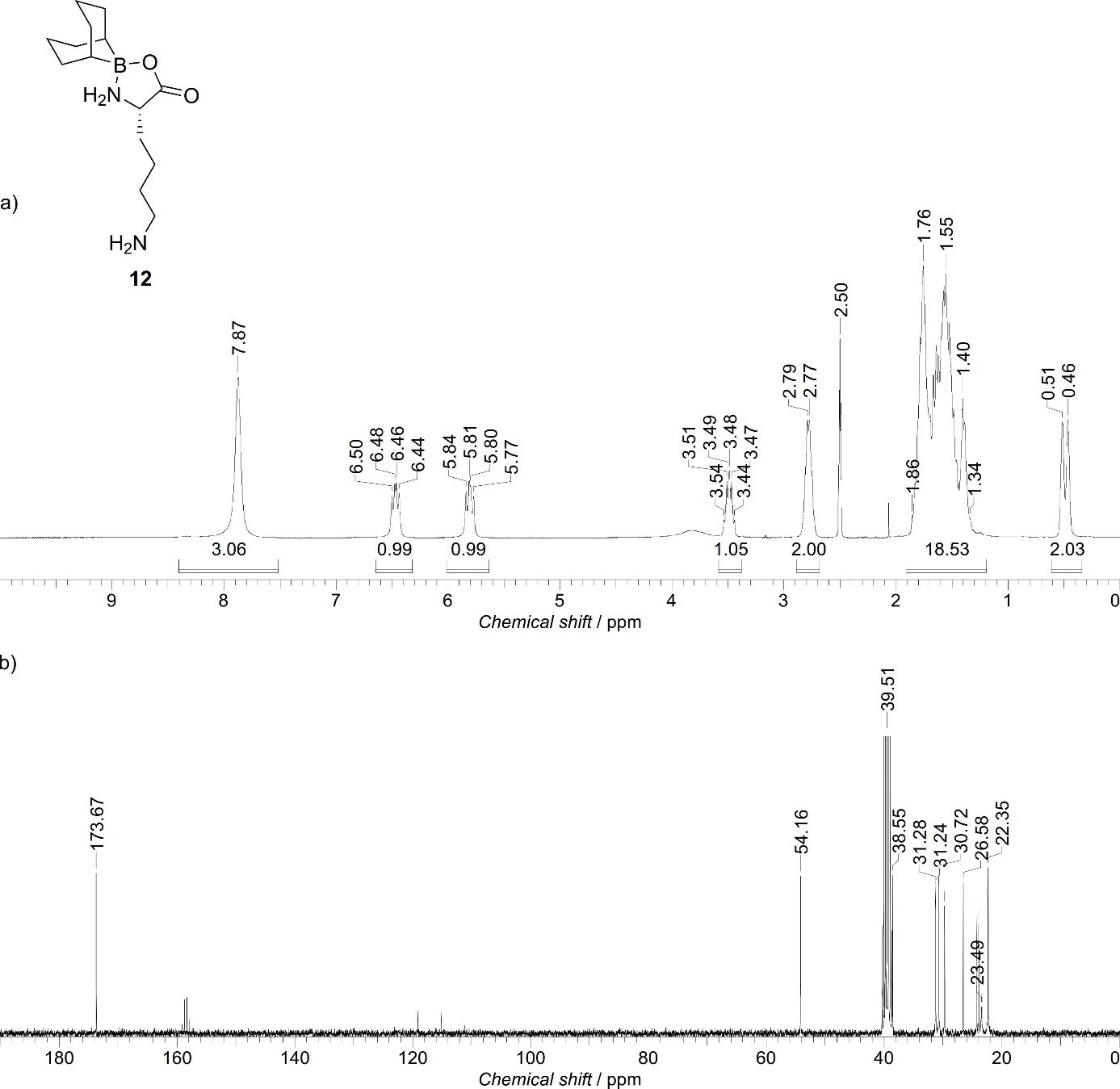


**Supporting Figure S17:** a) ^1^H-NMR spectrum (300 MHz, DMSO-d_6_) and b) ^13^C-NMR spectrum (75 MHz, DMSO-d_6_) of Lys-BBN (**12**).


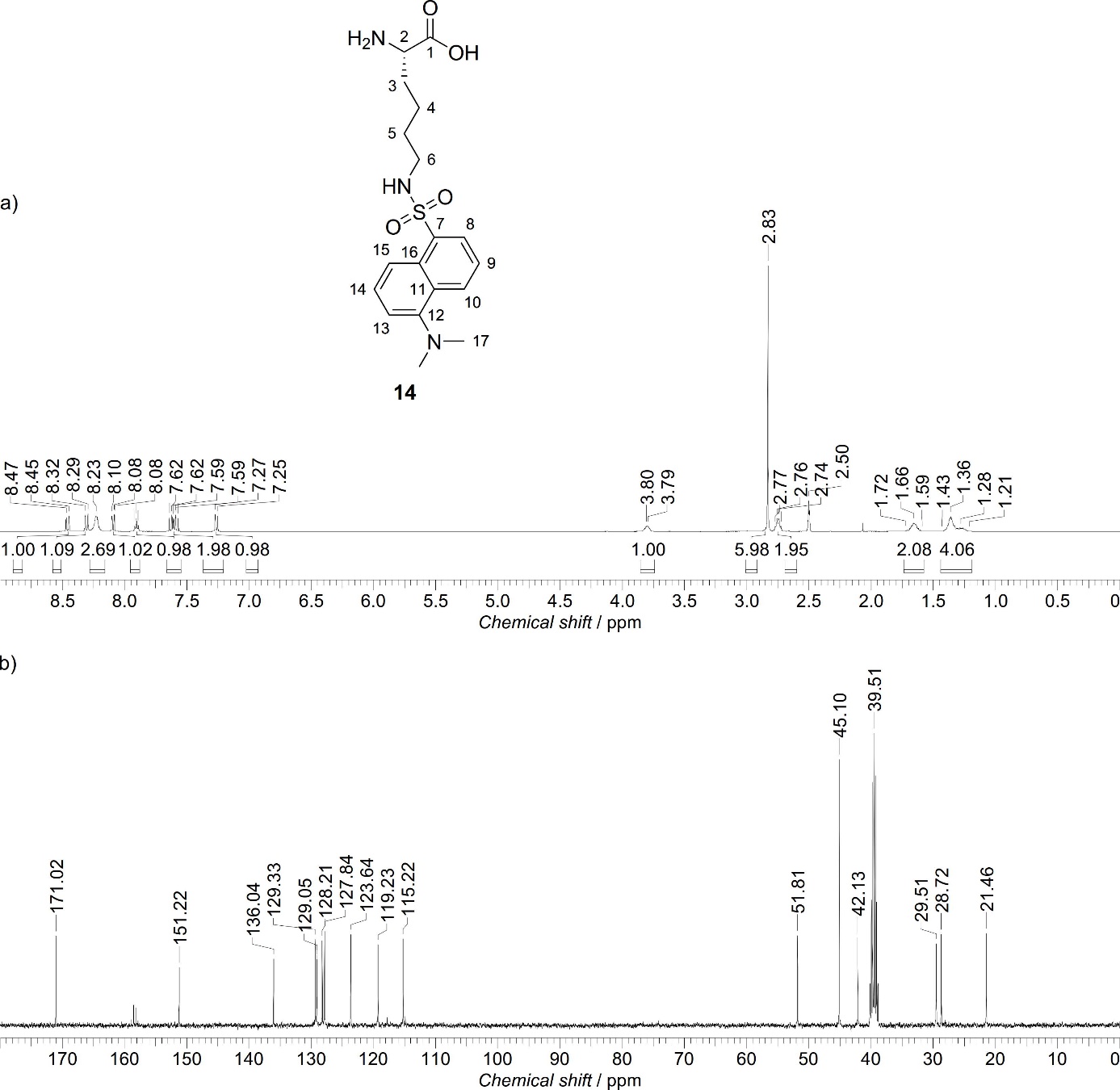


**Supporting Figure S18:** a) ^1^H-NMR spectrum (400 MHz, DMSO-d_6_) and b) ^13^C-NMR spectrum (101 MHz, DMSO-d_6_) of H-Lys(Dns)-OH (**14**).


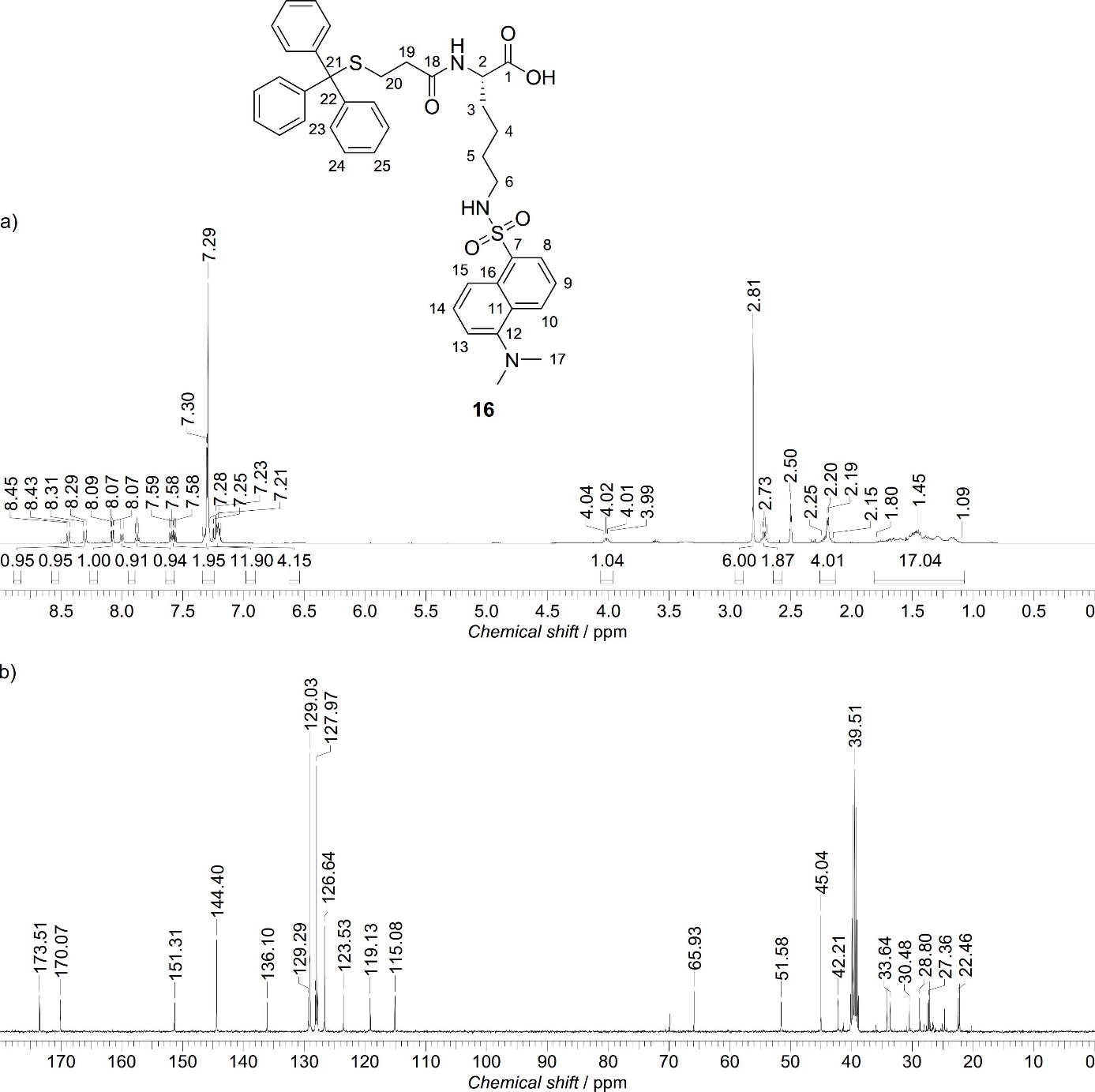


**Supporting Figure S19:** a) ^1^H-NMR spectrum (400 MHz, DMSO-d_6_) and b) ^13^C-NMR spectrum (101 MHz, DMSO-d_6_) of Trt-Mpa-Lys(Dns)-OH (**16**).

**
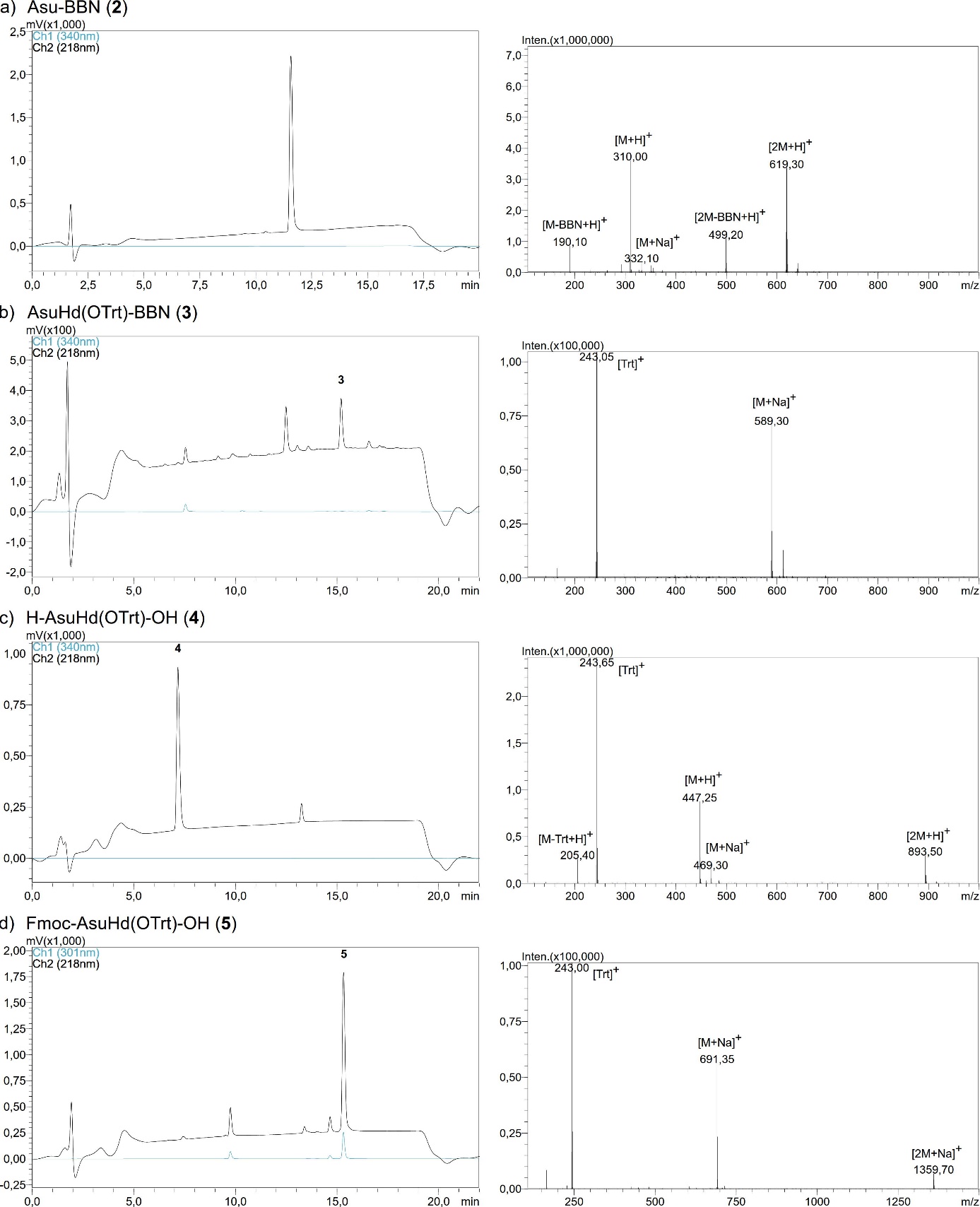
**

**Supporting Figure S20:** LC-MS analysis of a) Asu-BBN (**2**), b) AsuHd(OTrt)-BBN (**3**), c) H-AsuHd(OTrt)-OH (**4**) and d) Fmoc-AsuHd(OTrt)-OH (**5**).


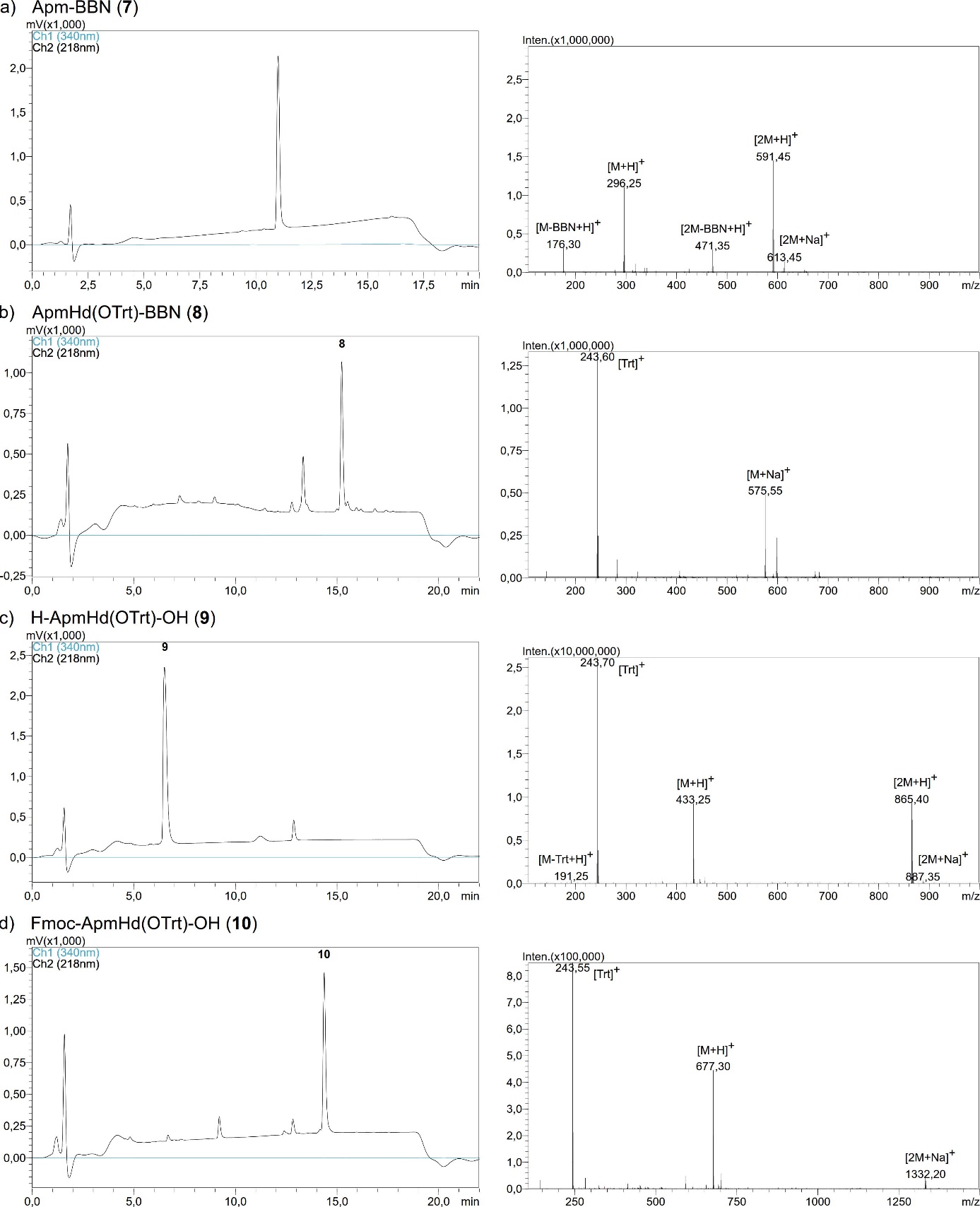


**Supporting Figure S21:** LC-MS analysis of a) Apm-BBN (**7**), b) ApmHd(OTrt)-BBN (**8**), c) H-ApmHd(OTrt)-OH (**9**) and d) Fmoc-ApmHd(OTrt)-OH (**10**).


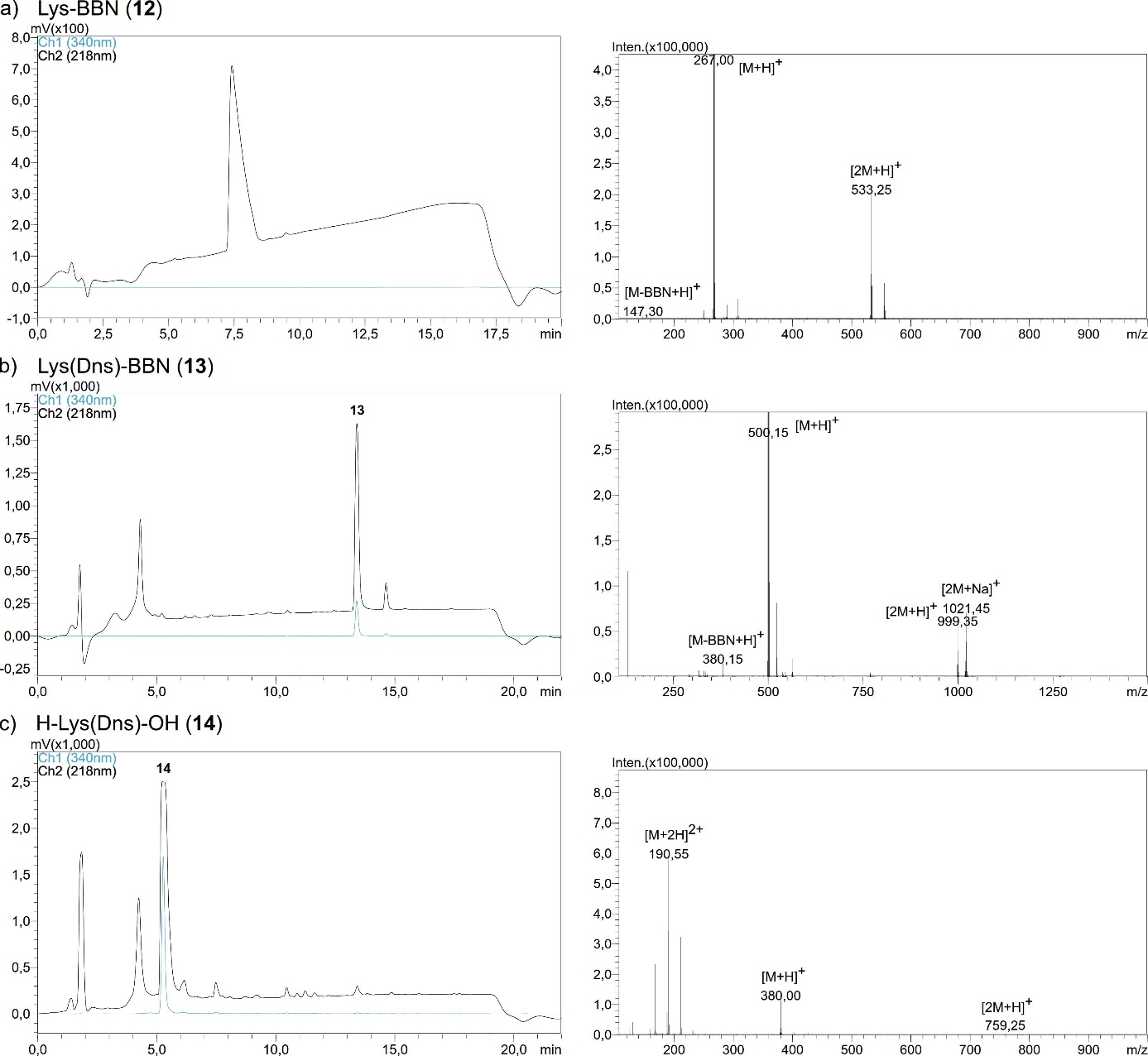


**Supporting Figure S22:** LC-MS analysis of a) Lys-BBN (**12**), b) Lys(Dns)-BBN (**13**) and c) H-Lys(Dns)-OH (**14**).


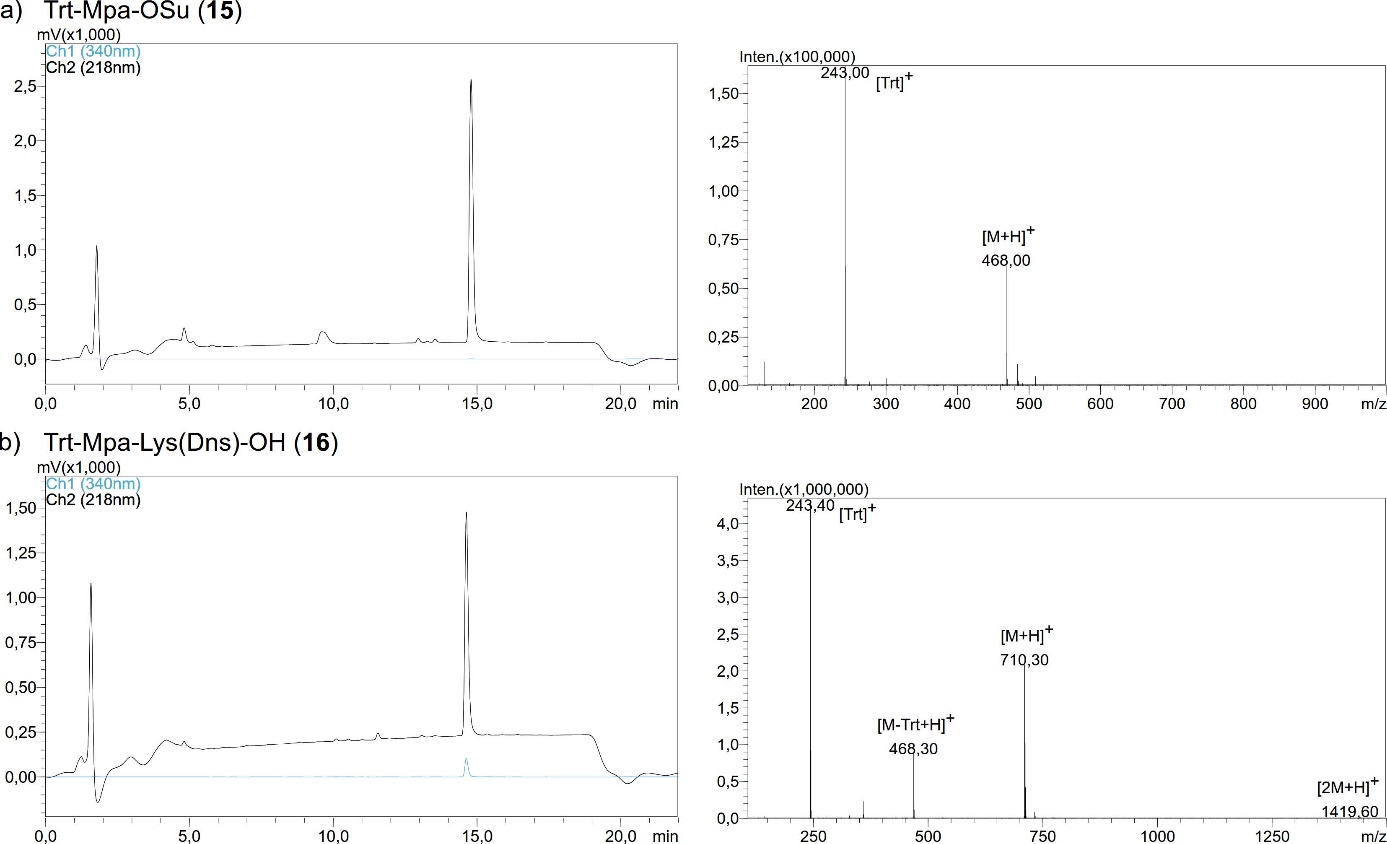


**Supporting Figure S23:** LC-MS analysis of a) Trt-Mpa-OSu (**15**) and b) Trt-Mpa-Lys(Dns)-OH (**16**).


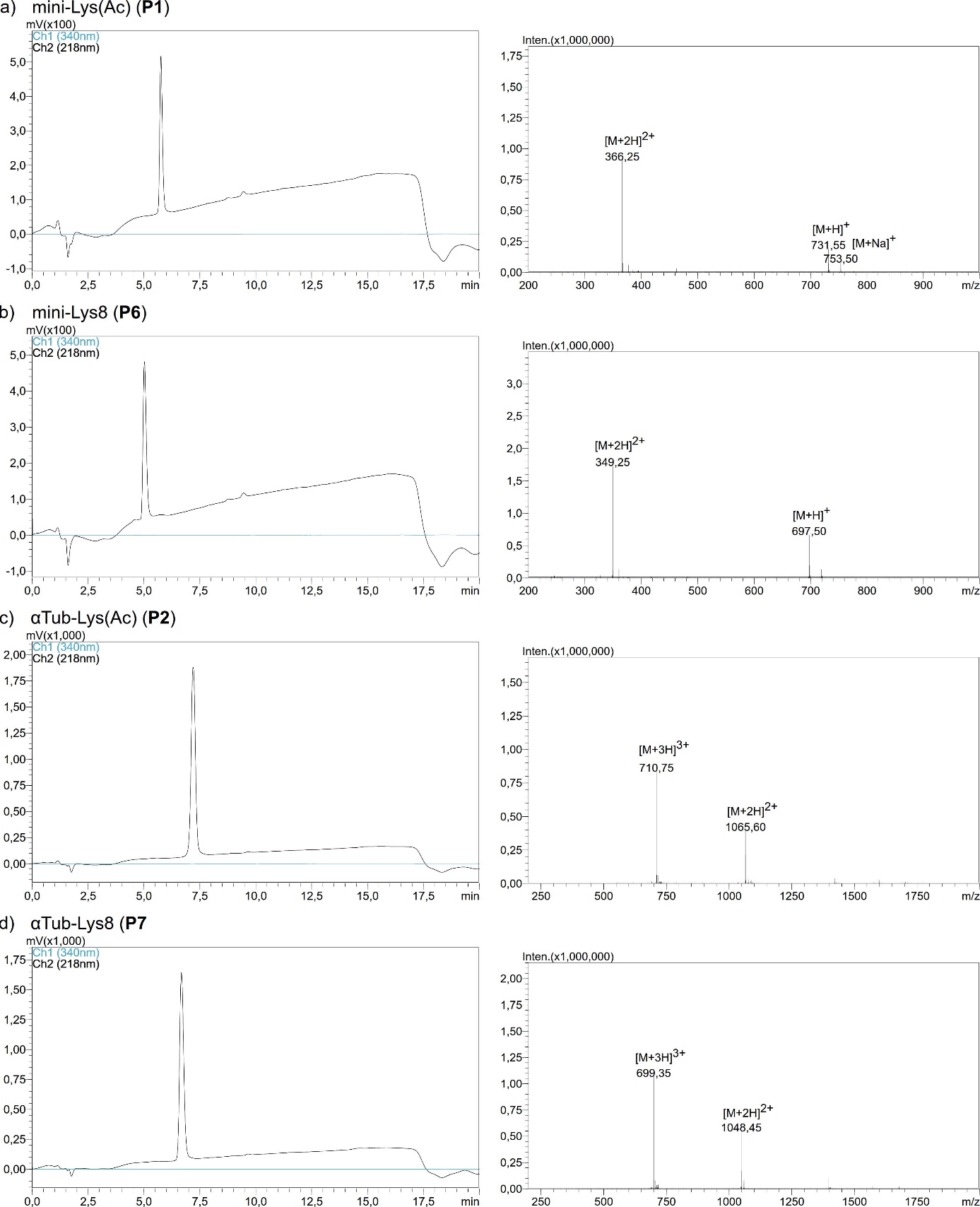


**Supporting Figure S24:** LC-MS analysis of substrate and isotopically labeled standard peptides for the MALDI-MS-based deacetylation assay: a) mini-Lys(Ac) (**P1**), b) mini-Lys8 (**P6**), c) αTub-Lys(Ac) (**P2**) and d) αTub-Lys8 (**P7**).


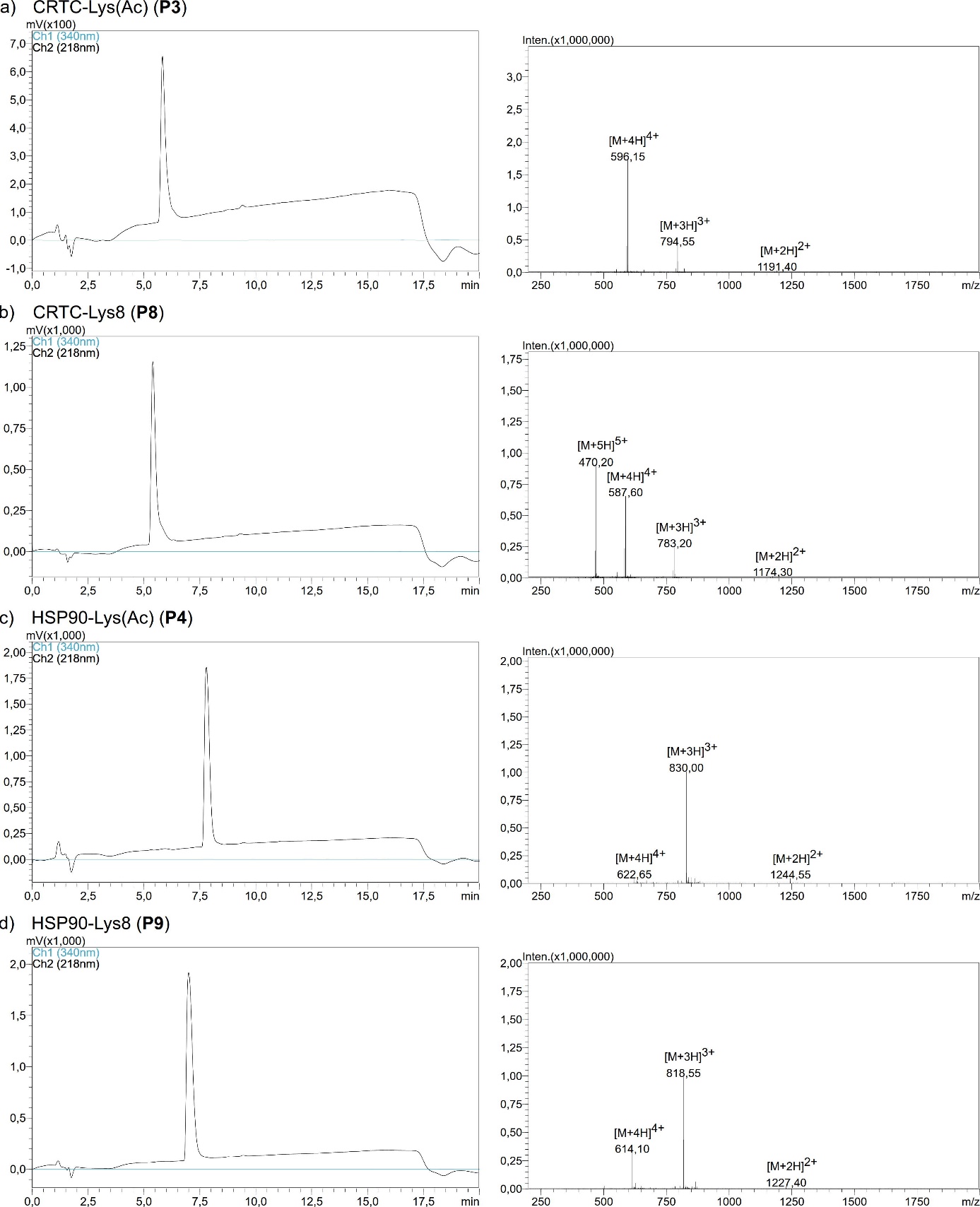


**Supporting Figure S25:** LC-MS analysis of substrate and isotopically labeled standard peptides for the MALDI-MS-based deacetylation assay: a) CRTC-Lys(Ac) (**P3**), b) CRTC-Lys8 (**P8**), c) HSP90-Lys(Ac) (**P4**) and d) HSP90-Lys8 (**P9**).


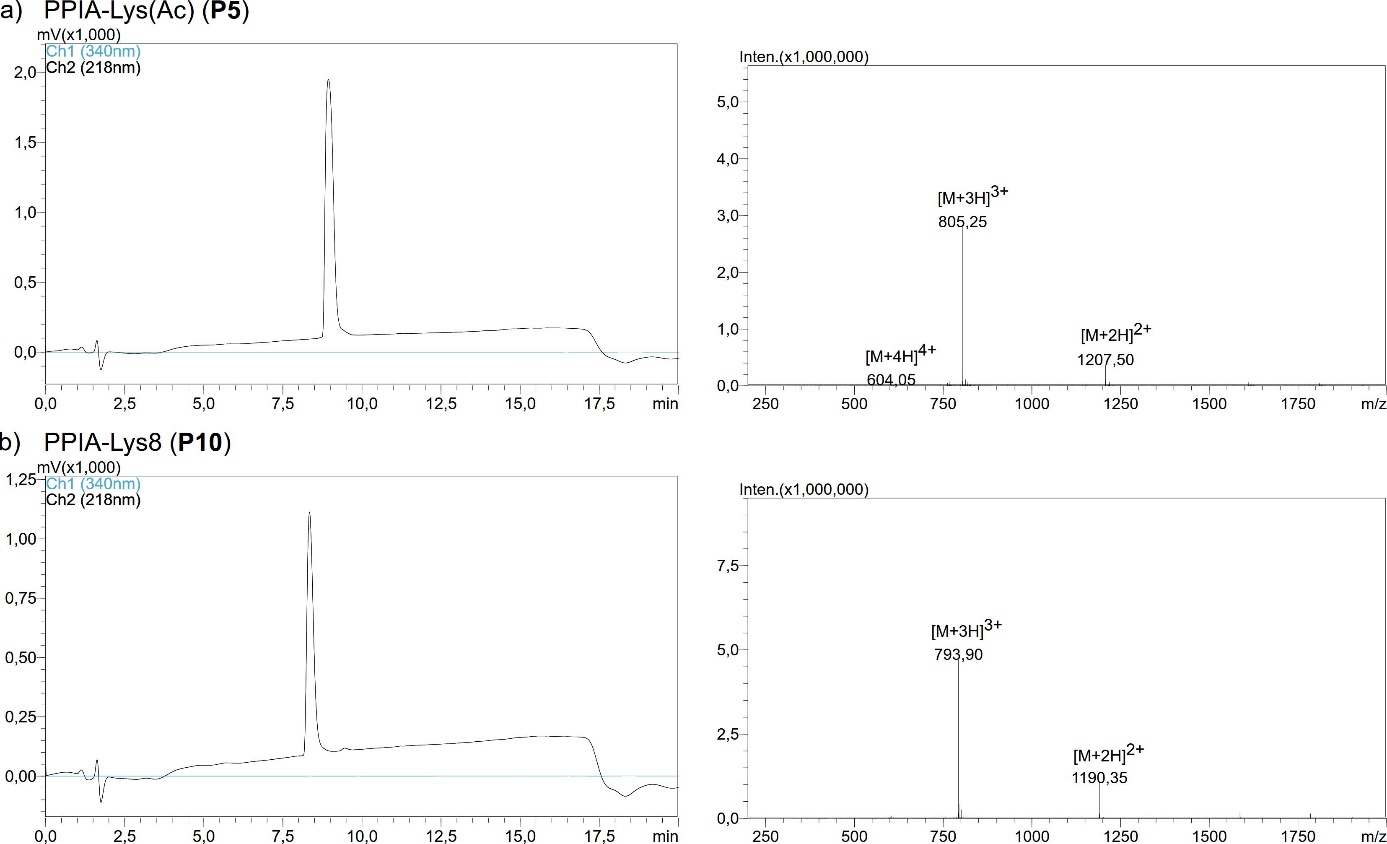


**Supporting Figure S26:** LC-MS analysis of substrate and isotopically labeled standard peptides for the MALDI-MS-based deacetylation assay: a) PPIA-Lys(Ac) (**P5**) and b) PPIA-Lys8 (**P10**).


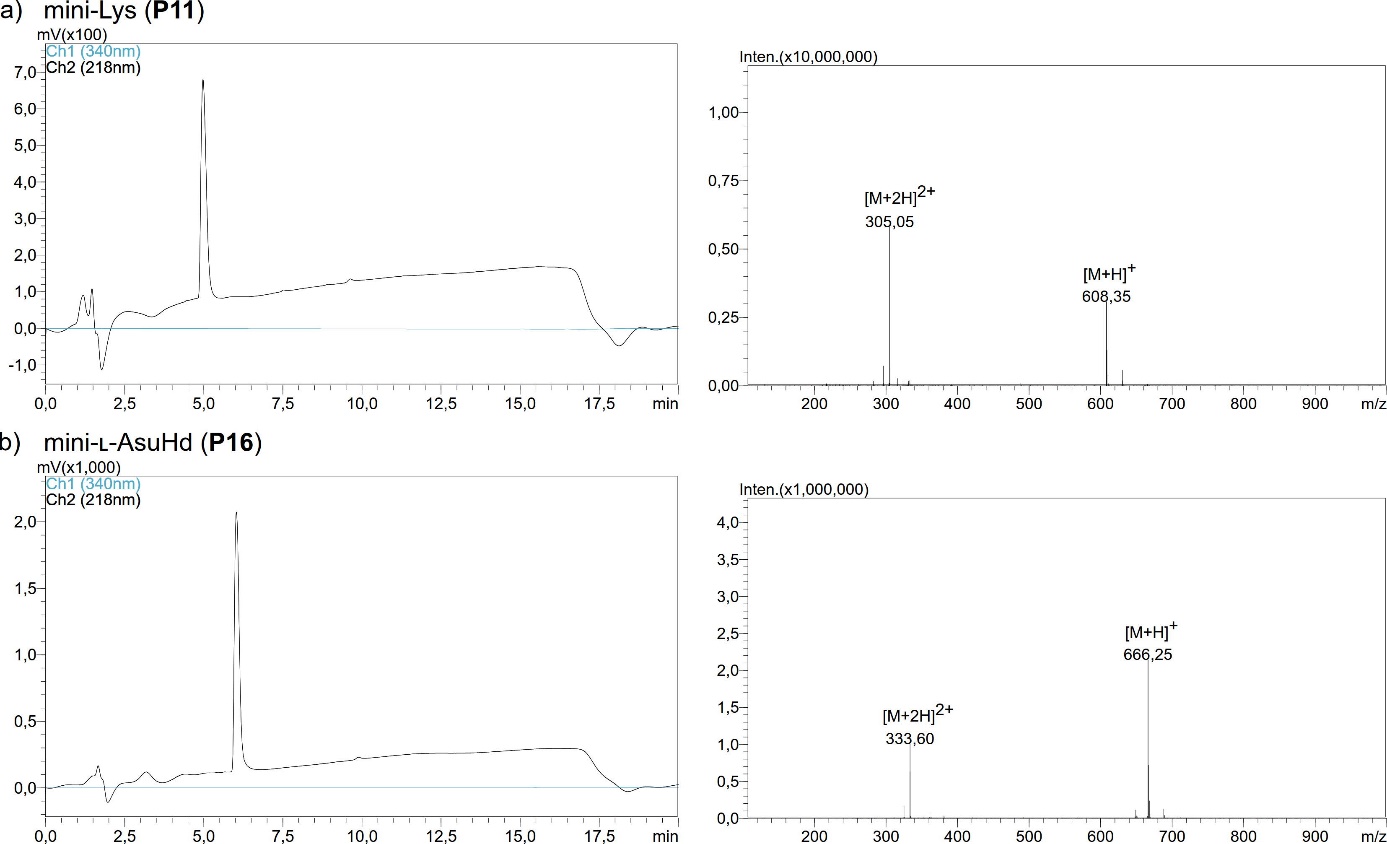


**Supporting Figure S27:** LC-MS analysis of mini-probes a) mini-Lys (**P11**), b) mini-AsuHd (**P16**).


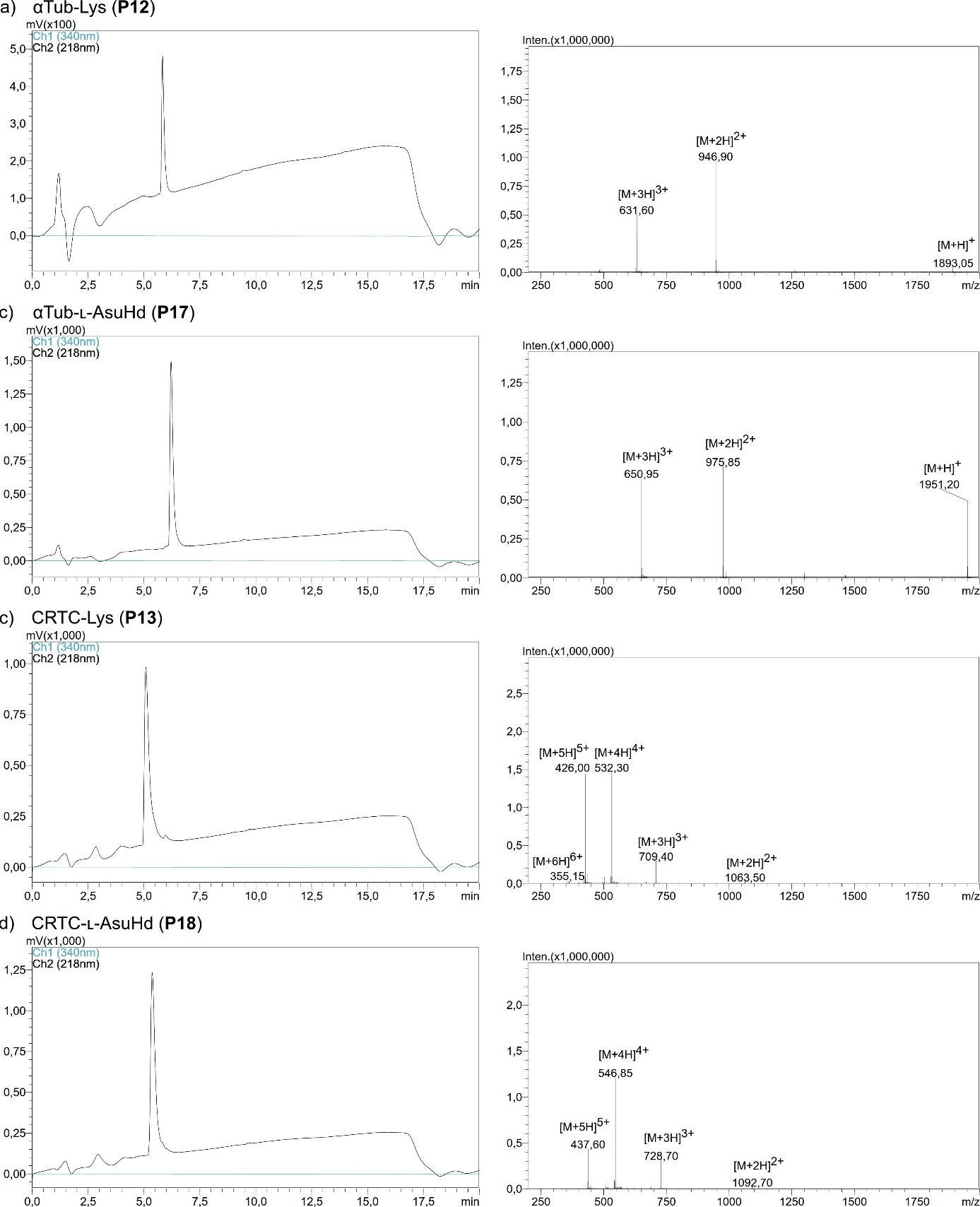


**Supporting Figure S28:** LC-MS analysis of selected HDAC6 substrate peptides a) αTub-Lys (**P12**), b) αTub-l-AsuHd (**P17**), c) CRTC-Lys (**P13**) and d) CRTC-l-AsuHd (**P18**).


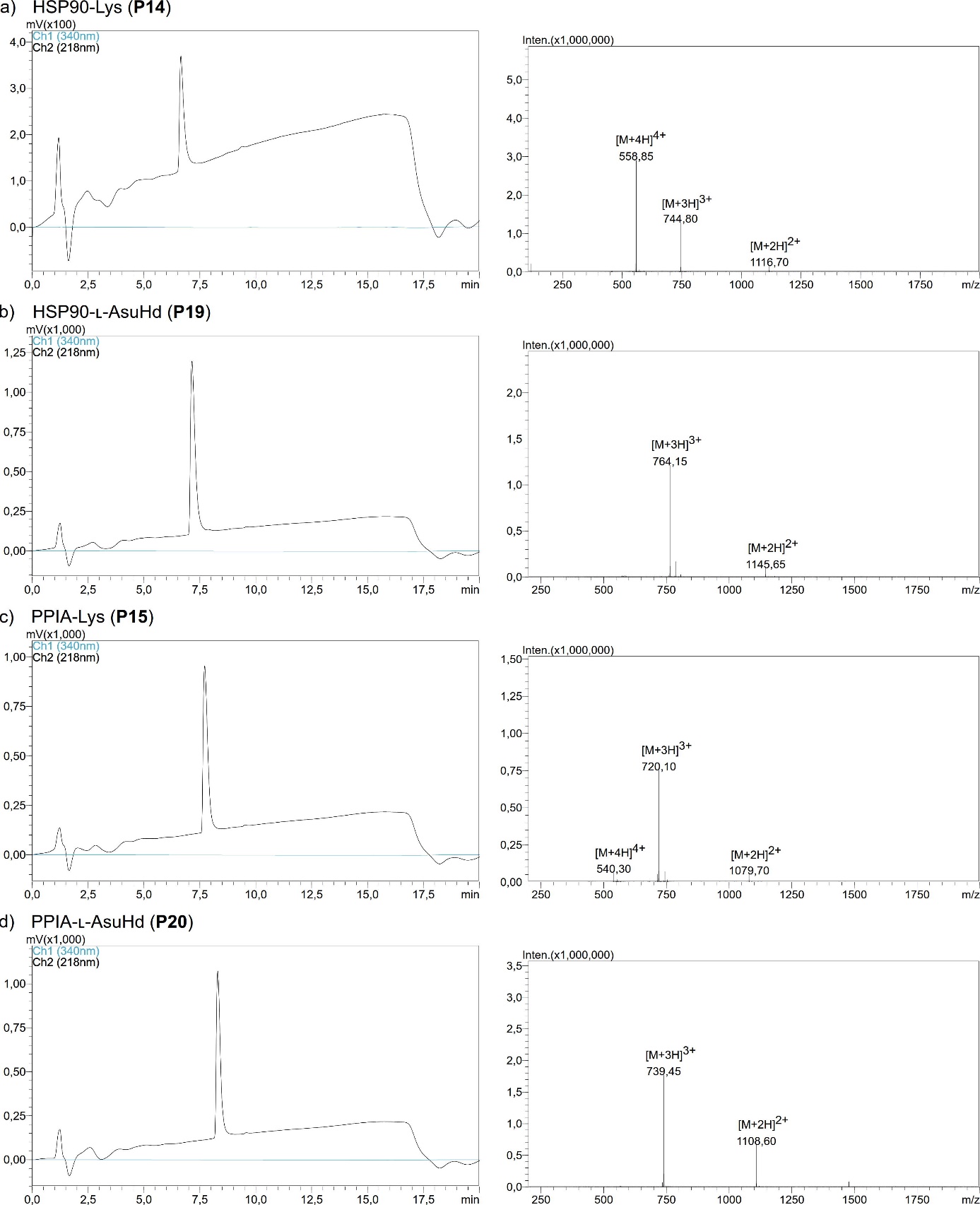


**Supporting Figure S29:** LC-MS analysis of selected HDAC6 substrate peptides a) HSP90-Lys (**P14**), b) HSP90-l-AsuHd (**P19**), c) PPIA-Lys (**P15**) and d) PPIA-l-AsuHd (**P20**).

**Full-size western blot data**

**
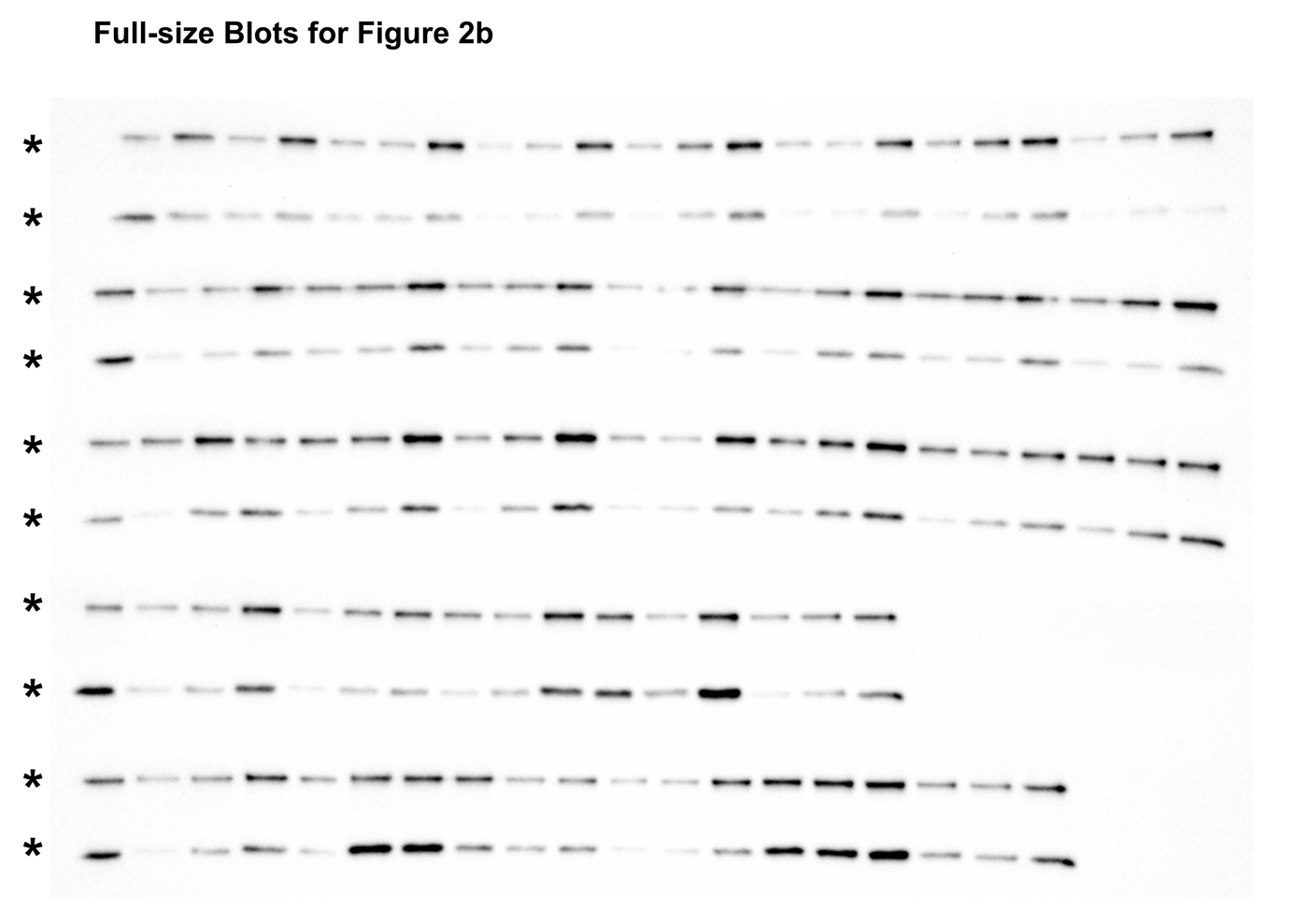
**

**Supporting Figure S30:** Full-size blots for Figure 2b. Membranes were cut into strips after transfer and immunostaining with anti-HDAC6 or anti-HDAC1 antibodies. The membrane strips were analyzed together with the same exposure time and instrument settings. Asterisks indicate positions of cropped bands shown in Figure 2b.


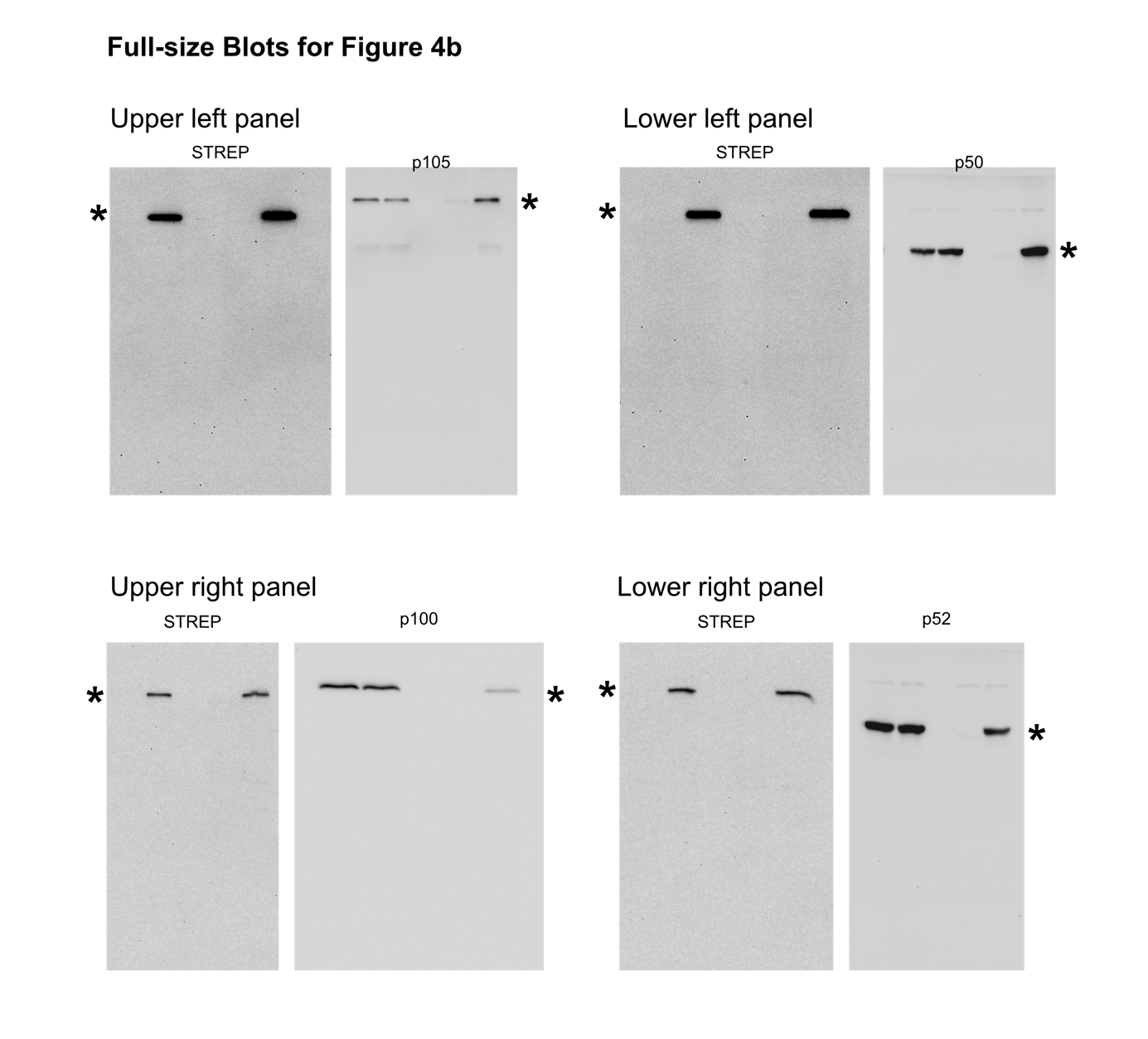


**Supporting Figure S31:** Full-size blots for Figure 4b. Asterisks indicate positions of cropped bands shown in Figure 4b.


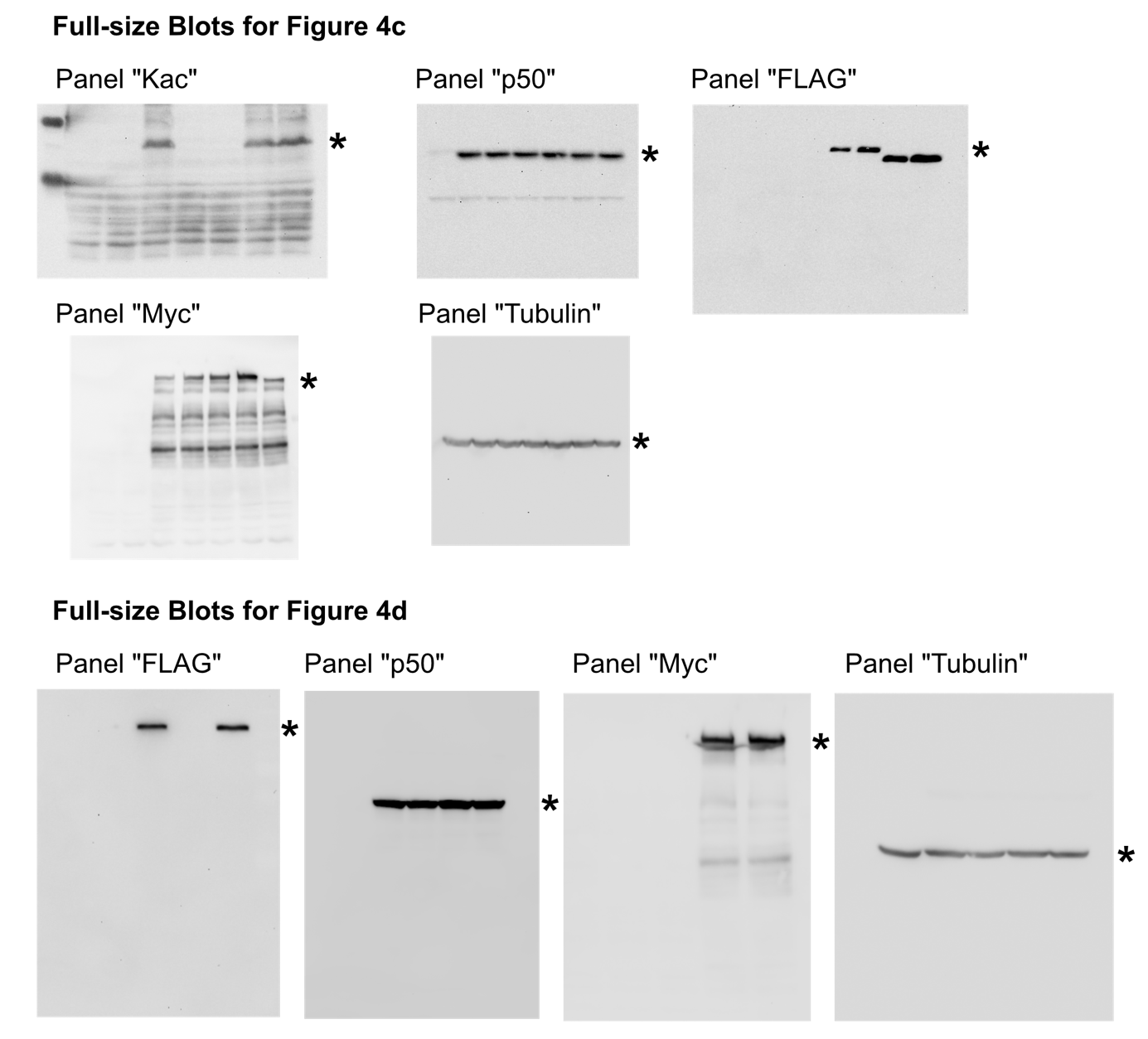


**Supporting Figure S32:** Full-size blots for Figure 4c and 4d. Asterisks indicate positions of cropped bands shown in Figure 4c and Figure 4d.


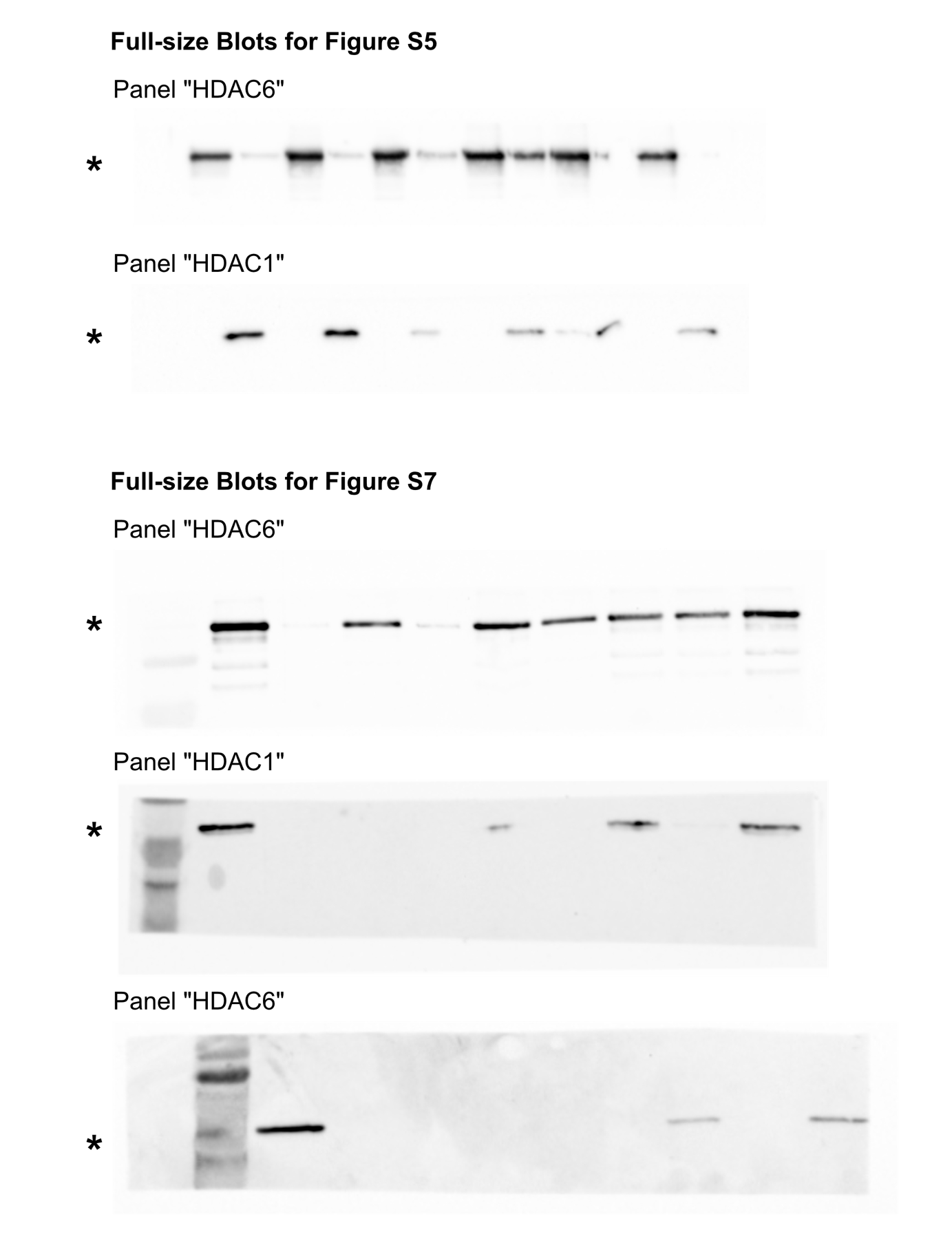


**Supporting Figure S33:** Full-size blots for supporting Figures S5 and S7. Membranes were cut into strips after transfer and individually stained with anti-HDAC6 or anti-HDAC1 antibodies. Asterisks indicate positions of cropped bands shown in supporting Figures S5 and S7.


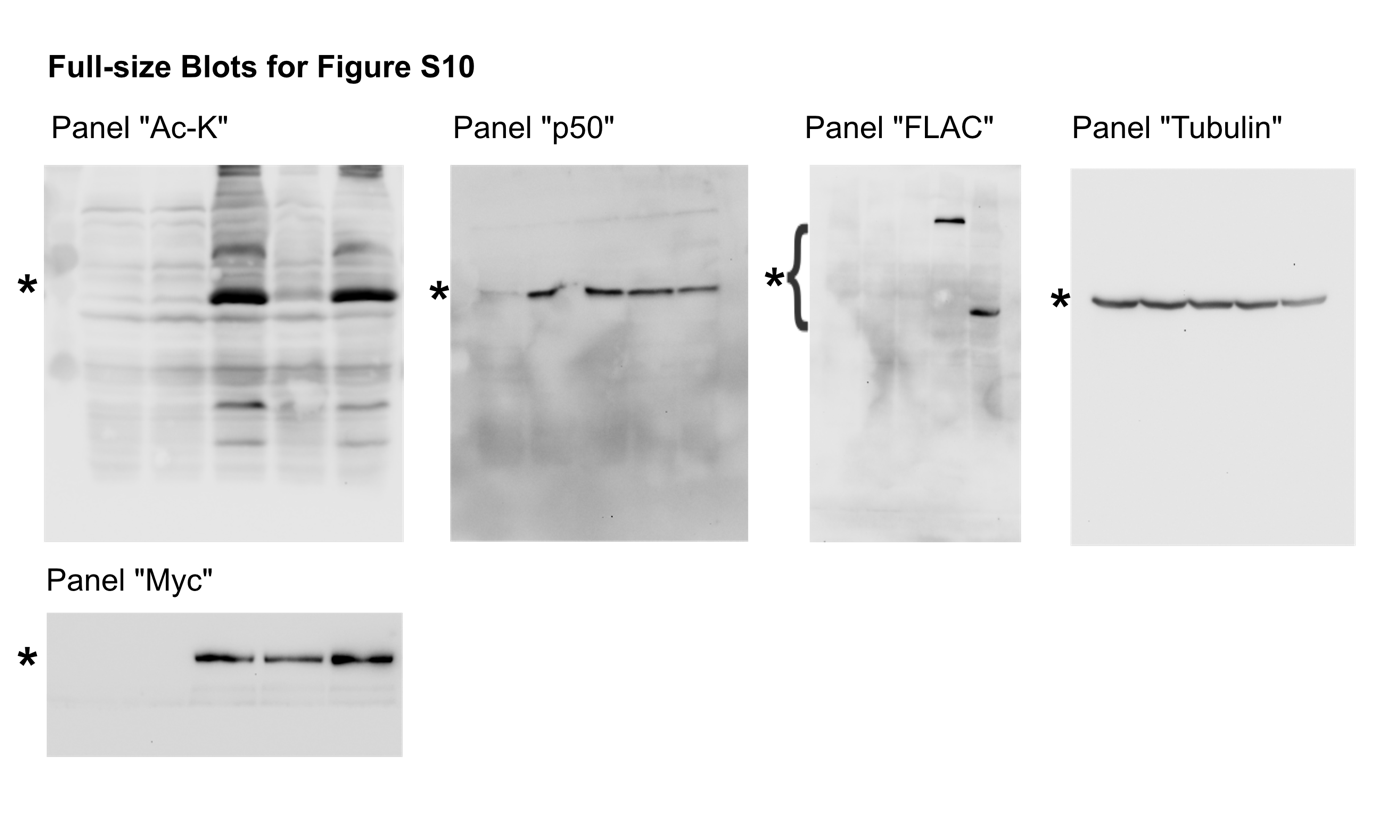


**Supporting Figure S34:** Full-size blots for supporting Figures S10. Asterisks indicate positions of cropped bands shown in supporting Figures S10.
